# Increasing forest disturbance enhances habitat suitability for Europe’s large herbivores

**DOI:** 10.1038/s41559-026-03096-0

**Published:** 2026-06-26

**Authors:** Julian Oeser, Rafał Kowalczyk, Dries Kuijper, Wiebke Neumann, Rudolf Reiner, Rupert Seidl, Cornelius Senf, Hendrik Bluhm, Nadège C. Bonnot, Luca Börger, Tomasz Borowik, Francesca Cagnacci, Marcin Churski, Benedikt Gehr, Marco Heurich, A. J. Mark Hewison, Klemen Jerina, Max Kröschel, Nicolas Morellet, Atle Mysterud, Nives Pagon, Gabriele Retez, Sebastian Seibold, Rita T. Torres, Alba Viana-Soto, Adrian Mihai Aldea, Roksana Baryło, Sophie Baur, Sebastian Catanoiu, Rok Černe, Marcin Grzegorzek, Dário Hipólito, Maciej Januszczak, Anders Jarnemo, Miloš Ježek, Artūras Kibiša, Daniel Klich, Alain Licoppe, Julien Lievens, Matthias-Claudio Loretto, Weronika Maślanko, Erling Meisingset, Rasmus Mohr Mortensen, András Náhlik, Wanda Olech, Astrid Olejarz, Federico Ossi, Algimantas Paulauskas, Maryline Pellerin, Kajetan Perzanowski, Wibke Peters, Mirosław Ratkiewicz, Thomas Rempfler, Sonia Saïd, Călin Constantin Șerban, Kastytis Šimkevičius, Jakub Skorupski, Maria Sobczuk, Nikica Šprem, Peter Sunde, Tamás Tari, Maciej Tracz, Magdalena Tracz, Aleksandra Wołoszyn-Gałęza, Tobias Kuemmerle

**Affiliations:** 1 Geography Department, Humboldt-Universität zu Berlin, Berlin, Germany; 2 Department of Community Ecology, Helmholtz Centre for Environmental Research, Halle, Germany; 3 Mammal Research Institute, Polish Academy of Sciences, Białowieża, Poland; 4 Swedish University of Agricultural Sciences, Umeå, Sweden; 5 Berchtesgaden National Park, Berchtesgaden, Germany; 6 Ecosystem Dynamics and Forest Management Group, School of Life Sciences, Technical University of Munich, Freising, Germany; 7 Earth Observation for Ecosystem Management Group, School of Life Sciences, Technical University of Munich, Freising, Germany; 8 Université de Toulouse, INRAE, CEFS, Castanet-Tolosan, France; 9 Department of Biosciences, Swansea University, Swansea, UK; 10 Animal Ecology Unit, Research and Innovation Centre, Fondazione Edmund Mach, San Michele all’Adige, Italy; 11 Department of Evolutionary Biology and Environmental Studies, University of Zurich, Zurich, Switzerland; 12 Department of National Park Monitoring and Animal Management, Bavarian Forest National Park, Grafenau, Germany; 13 Chair of Wildlife Ecology and Wildlife Management, Faculty of Environment and Natural Resources, University of Freiburg, Freiburg, Germany; 14 Department of Forestry and Wildlife Management, University of Inland Norway, Koppang, Norway; 15 Department of Forestry and Renewable Forest Resources, Biotechnical Faculty, University of Ljubljana, Ljubljana, Slovenia; 16 Forest Research Institute Baden-Württemberg, Freiburg, Germany; 17 Centre for Ecological and Evolutionary Synthesis, Department of Biosciences, University of Oslo, Oslo, Norway; 18 Slovenian Forest Service, Ljubljana, Slovenia; 19 WWF Romania, Bucharest, Romania; 20 Institute of Evolutionary Ecology and Conservation Genomics, University of Ulm, Ulm, Germany; 21 Centre for Environmental and Marine Studies and Department of Biology, University of Aveiro, Aveiro, Portugal; 22 Foundation Conservation Carpathia, Brașov, Romania; 23 West Pomeranian Nature Society, Szczecin, Poland; 24 Bavarian State Institute for Forestry, Freising, Germany; 25 Vanatori Neamt Nature Park, Vanatori Neamț, Romania; 26 Romsilva, Bucharest, Romania; 27 Veterinary Biology Unit, Faculty of Veterinary Medicine, University of Zagreb, Zagreb, Croatia; 28 Museum and Institute of Zoology, Polish Academy of Sciences, Warsaw, Poland; 29 School of Business, Innovation and Sustainability, Halmstad University, Halmstad, Sweden; 30 Department of Game Management and Wildlife Biology, Faculty of Forestry and Wood Sciences, Czech University of Life Sciences, Prague, Czechia; 31 Faculty of Forest Sciences and Ecology, Agriculture Academy, Vytautas Magnus University, Kaunas, Lithuania; 32 Institute of Animal Sciences, Warsaw University of Life Sciences, Warsaw, Poland; 33 Department of Natural and Agricultural Environment Studies, Public Service of Wallonia, Gembloux, Belgium; 34 Research Institute of Wildlife Ecology, Department of Interdisciplinary Life Sciences, University of Veterinary Medicine Vienna, Vienna, Austria; 35 Department of Animal Ethology and Wildlife Management, University of Life Sciences in Lublin, Lublin, Poland; 36 Division of Forest and Forest Resources, Norwegian Institute of Bioeconomy Research, Ås, Norway; 37 Department of Ecoscience, Aarhus University, Aarhus, Denmark; 38 Sapientia Hungarian University of Transylvania, Cluj-Napoca, Romania; 39 University of Sopron, Sopron, Hungary; 40 Faculty of Natural Sciences, Vytautas Magnus University, Kaunas, Lithuania; 41 Department of Research and Scientific Support, French Biodiversity Agency, Châteauvillain, France; 42 Institute of Biological Sciences, Catholic University of Lublin, Lublin, Poland; 43 Faculty of Biology, University of Białystok, Białystok, Poland; 44 Swiss National Park, Zernez, Switzerland; 45 University of Szczecin, Szczecin, Poland; 46 Faculty of Agriculture, University of Zagreb, Zagreb, Croatia; 47 Integrative Research Institute on Transformations of Human-Environment Systems, Humboldt-Universität zu Berlin, Berlin, Germany

**Keywords:** Forest ecology, Ecology

## Abstract

Forest disturbances have increased in many regions, but how they impact habitat suitability for wildlife remains poorly understood. Here, by combining tracking data on 3,069 individuals of four ungulate species (European bison, moose, red deer and roe deer) with satellite-based maps, we perform a continental, multi-decadal assessment of large herbivore responses to forest disturbance. Despite strong intraspecific variation, all species show an increased selection of disturbed areas for ≥35 years after disturbance. Although the patterns closely reflect species-specific foraging strategies, all species selected more strongly for smaller disturbance patches, depending on the availability of alternative foraging habitats (grasslands and croplands). Model projections across the species’ range extents show positive but regionally varying effects of forest disturbances on habitat suitability between 2000 and 2023. Our findings indicate that forest disturbances can attract large herbivores and that the recent increase in forest disturbances improved habitat suitability for our study species across Europe, highlighting the importance of considering long-term disturbance-related dynamics for wildlife and forest management. Given expected future increases in disturbance, resulting habitat improvements could amplify conflicts with forestry, but also contribute to restoring large herbivores and their ecological functions.

## Abstract (web summary)

Forest disturbances may impact wildlife habitat suitability in complex ways. Here the authors provide evidence that disturbance enhances forest habitat suitability for Europe’s large ungulates over time, mediated by species-specific behavioural preferences.

## Main

Forest disturbances, caused by natural agents (for example, storms, fire or insects) or forest management (for example, clear cutting or salvage logging) drive forest dynamics and have long-lasting effects on carbon cycling, biodiversity and forest structure^1–3^. Over recent decades, intensifying forest use and higher rates of natural disturbances—attributed primarily to climatic extremes^4,5^—have caused a drastic increase in the extent of forest disturbances in some regions^6,7^. For example, canopy mortality doubled in Europe’s temperate forests between 1984 and 2016 (ref. ^8^) and the forest area disturbed by fires globally was 2.2 times greater in 2023–2024 than the long-term average since 2001 (ref. ^9^). In addition, forest disturbances are expected to further increase under climate change^10^.

Large herbivores play key roles in shaping forest ecosystems and are also of considerable cultural and economic importance^11–13^. Forest disturbances may strongly impact large herbivore habitat suitability, since they directly affect the fundamental trade-off faced by many large herbivores between accessing forage and cover^14^. By killing canopy trees, forest disturbances increase light availability in the understory, encouraging the growth of early-successional plants and resulting in higher forage availability for many species^15,16^. Due to the seasonal variation in foraging preferences^17^, the suitability of disturbed areas for large herbivores may vary considerably throughout the year^18^. Disturbances can intermittently reduce the availability of vegetation cover and create open habitats in which animals are exposed to predation, human activities, thermal extremes and snow^19–21^. Yet, depending on disturbance characteristics (for example, disturbance patch size and remaining deadwood) and vegetation recovery, disturbed areas can provide habitats offering both forage and cover^22^. Thus, large herbivore responses to forest disturbance can be expected to strongly depend on species’ feeding ecology and disturbance characteristics and further show considerable seasonal variation.

Understanding large herbivore responses to forest disturbance is critical due to the strong impact of herbivory in disturbed areas, as browsing can inhibit tree regeneration and alter species composition^23^. High browsing intensities often create conflicts with forestry^24^, but herbivory may also contribute to maintaining open habitats, with potential positive effects on biodiversity and ecosystem resilience^25,26^. A better understanding of how forest disturbances shape large herbivore habitat suitability is thus important to inform debates about how large herbivore management may contribute to forestry and forest restoration goals.

Although several studies have examined the effects of forest disturbance on large herbivore species at local or regional scales^18,22,24^, our understanding remains limited due to a lack of systematic assessments covering broad spatial extents, multiple species and long time periods. Thus, it also remains unclear how large herbivore habitat suitability has changed in regions that have experienced marked increases in forest disturbances over recent decades. Increasing disturbance rates may benefit large herbivores that capitalize on early-successional vegetation^18^, but extensive forest disturbance has also been identified as a threat to some large herbivore populations^27,28^. The growing availability of animal tracking data collected from a wide range of large herbivore populations^29^, along with improved characterizations of forest disturbance dynamics from satellite time series^30,31^, provides tremendous but so far untapped opportunities to fill these knowledge gaps^18^.

In this Article, we describe a continental-scale, multi-species and long-term assessment of forest disturbance impacts on large herbivore habitat selection and suitability across Europe. We focused on four native large herbivore species with wide potential natural ranges (that is, the European bison (*Bison bonasus*), moose (*Alces alces*), red deer (*Cervus elaphus*) and roe deer (*Capreolus capreolus*)), reflecting gradients in terms of foraging strategies^17^. European forests are heavily managed, with few intact old-growth forests^32^, and a majority of disturbances are caused by human forest use^33^. Forest disturbances in Europe have increased markedly over recent decades^7,8,30^, with increasing natural disturbances and intensifying forest use interacting as drivers^33^. Over the same period, the populations of red deer and roe deer have grown considerably^34^, intensifying human–wildlife conflicts and raising concerns about potential negative impacts on forest regeneration^35,36^. Europe’s largest herbivores, European bison and moose, remain absent from large parts of their former ranges, but have been recovering and expanding their distributions recently^37^, providing opportunities for restoring their ecological roles^38,39^. In our analysis, we addressed the following research questions:

1. How do large herbivores respond to forest disturbances over time and how do responses vary across seasons, disturbance characteristics and environmental gradients?
2. How did recent forest disturbance dynamics alter habitat suitability for large herbivores across species’ current and potential range extents?

## Results

### Temporal responses to forest disturbance

**Extended Data Fig. 1:**
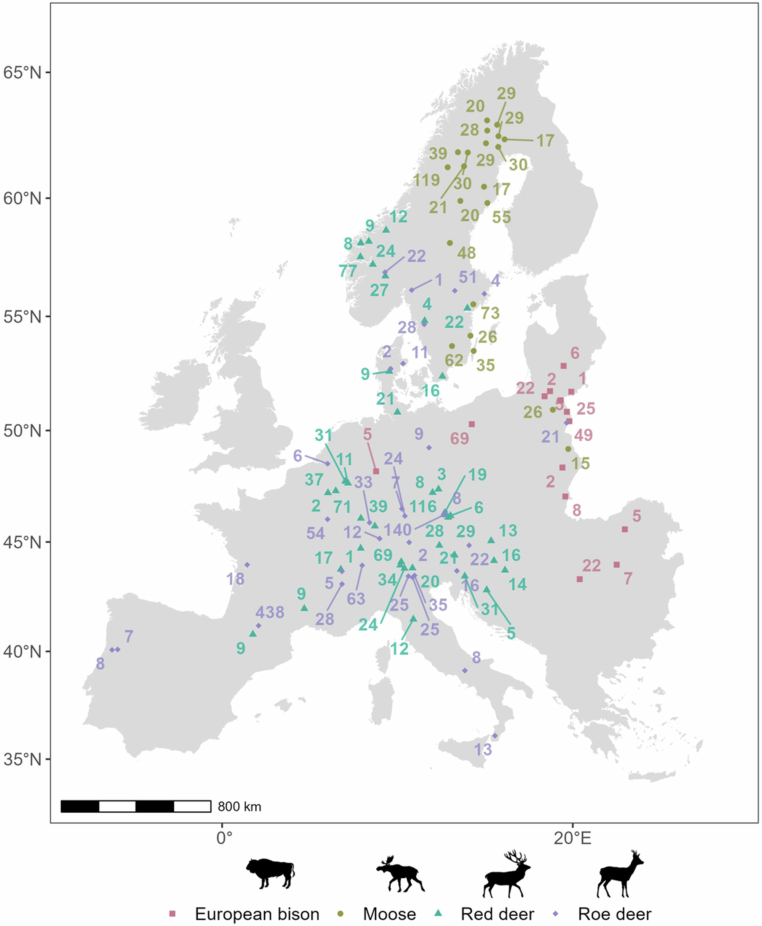
Number of tracked animals per species and study site. Coloured shapes indicate the location of study sites, and numbers indicate the number of individuals tracked per site. Country shapefile data from GADM under a Creative Commons license CC BY-SA 2.0. Animal silhouettes from PhyloPic under a Creative Commons license: *C. elaphus* and *C. capreolus*, Ferran Sayol (CC0 1.0); *A. alces*, xgirouxb (PDM 1.0); *B. bonasus*, Steven Traver (PDM 1.0).

**Extended Data Fig. 2:**
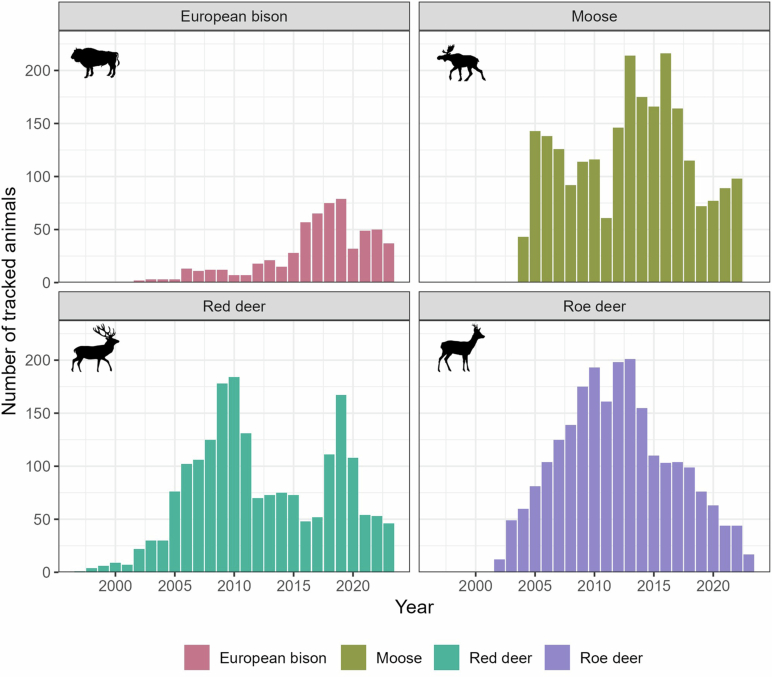
Number of tracked animals per species and year. Coloured bars indicate the number of tracked individuals. Animal silhouettes from PhyloPic under a Creative Commons license: *C. elaphus* and *C. capreolus*, Ferran Sayol (CC0 1.0); *A. alces*, xgirousxb (PDM 1.0); *B. bonasus*, Steven Traver (PDM 1.0).

We used one of the largest animal-tracking datasets hitherto compiled for large herbivores (3,069 tracked animals and 19.3 million locations; Extended Data Figs. 1 and 2, and Table 1) to build habitat selection models for European bison, moose, red deer and roe deer based on the random forest algorithm. We characterized habitat dynamics based on datasets derived from satellite image time series, including annual, high-resolution maps of forest disturbances^31^, spectral vegetation indices^18^ and other environmental predictors (Extended Data Table 1). The forest disturbance maps captured stand-replacing forest disturbance events at 30 m spatial resolution annually between 1986 and 2023. In the context of our analysis, we considered all areas with a detected disturbance event as disturbed forest; all other areas were considered undisturbed forest. Using selection strength as an indicator of relative habitat suitability^40^, we created monthly predictions of habitat suitability between 2000 and 2023 across representative samples of disturbed and undisturbed forest areas within the species’ current and potential range extents (that is, estimates of where species would occur without human influence) in Europe. To assess the species-specific responses to forest disturbances over time, we summarized habitat suitability dynamics after disturbances, focusing on the current range extents of species. To aid comparability across species, we quantified suitability changes as relative changes after disturbance and expressed effects in standard deviations of forest habitat suitability.

**Table 1:** Overview of the global positioning system tracking data

Species	Study sites	Animals (female/male)	Monitoring period	Mean sampling rate (h)	Raw locations	Used locations
European bison	14	228 (175/53)	2005–2023	7.86	2,389,076	113,531
Moose	21	768 (592/176)	2004–2022	12.1	1,858,537	397,920
Red deer	39	918 (685/233)	1997–2023	2.28	8,203,505	427,658
Roe deer	31	1143 (713/430)	2002–2023	2.88	6,854,404	709,553

The number of animals per species and used locations correspond to the filtered datasets used for training habitat selection models, whereas the raw locations correspond to the full, unfiltered data.

**Fig. 1:**
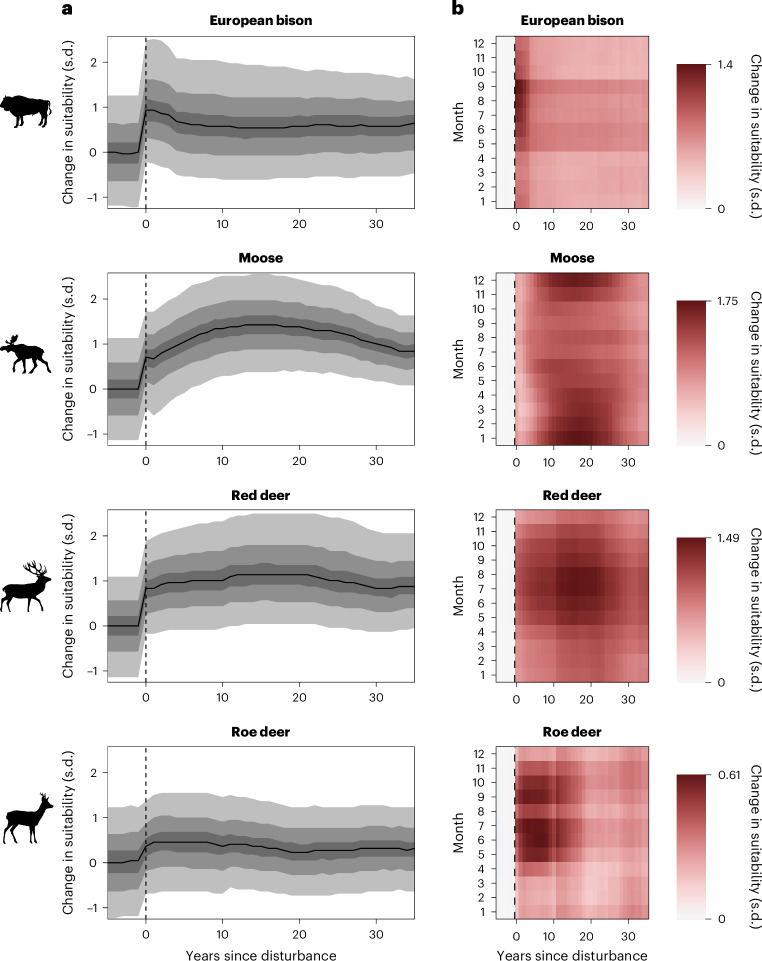
Responses of four large herbivore species to forest disturbances over time in Europe. To facilitate comparisons across species, disturbance responses are shown as relative changes in the standardized habitat suitability compared with the 5-year average before a disturbance event, where standardization is based on the distribution of forest habitat suitability in the year 2000. **a**, Long-term disturbance responses of habitat suitability, with the black line indicating median suitability and the grey ribbons the 10–90th, 25–75th and 40–60th percentile ranges, respectively. **b**, Seasonal variation in disturbance responses, calculated as medians by month (note that the colour scales vary by species to better highlight seasonal variation).

**Extended Data Fig. 3:**
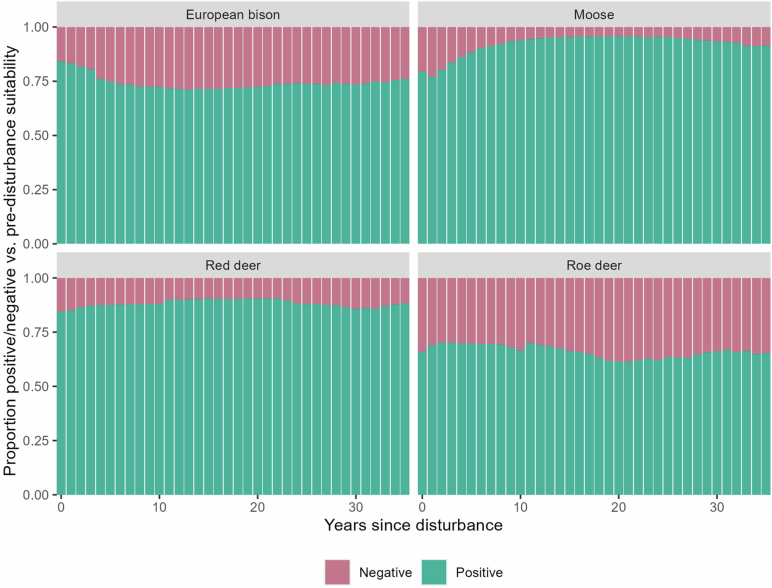
Direction of disturbance responses. Coloured bars indicate the proportion of positive/negative suitability values compared to pre-disturbance conditions across time since disturbance.

**Extended Data Fig. 4:**
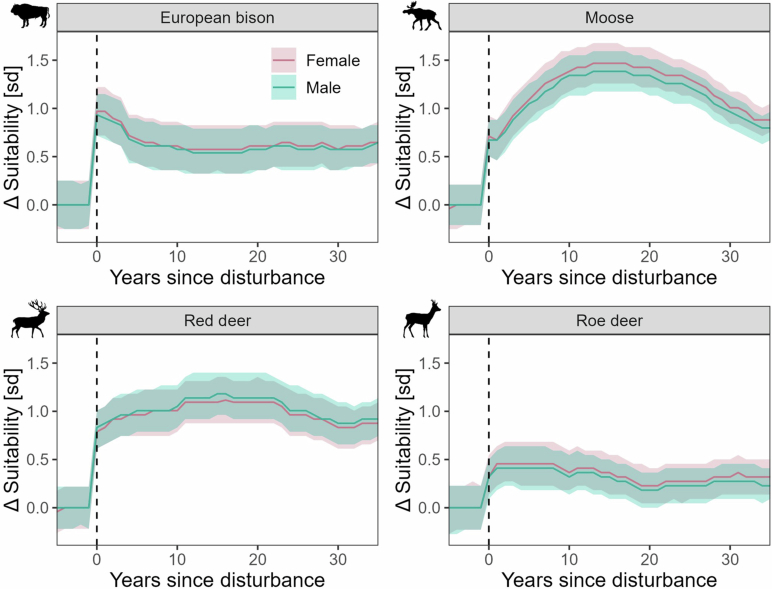
Disturbance responses by sex. Disturbance responses are shown as relative changes in standardized habitat suitability compared to the five-year average prior to a disturbance event, where standardization is based on the distribution of forest habitat suitability in the year 2000. Coloured lines indicate median suitability, and the coloured ribbons the 40th to 60th percentile ranges per sex. Animal silhouettes from PhyloPic under a Creative Commons license: *C. elaphus* and *C. capreolus*, Ferran Sayol (CC0 1.0); *A. alces*, xgirousxb (PDM 1.0); *B. bonasus*, Steven Traver (PDM 1.0).

All four species showed strong intraspecific variation, but with overall positive responses to forest disturbance (Fig. 1). On average, the increase in habitat suitability following disturbance was highest for moose (up to 1.43 standard deviations in median annual suitability), followed by red deer (1.14), European bison (0.93) and roe deer (0.46). For European bison and roe deer, the increase in habitat suitability was highest immediately after a disturbance event, with a relatively rapid decrease for European bison and a plateau for ~5–7 years after disturbance for roe deer. In contrast, there was a delay in the response of red deer and moose, for which the suitability of disturbed areas was highest ~10–20 years after the disturbance event. For all species except moose, the habitat suitability of disturbed areas was highest during the growing season (around May to November). Despite overall positive responses, our models predicted decreased habitat suitability compared with pre-disturbance conditions in 36, 29, 17 and 13% of cases for roe deer, European bison, red deer and moose, respectively (Extended Data Fig. 3). Responses to forest disturbance were highly similar for males and females across all species (Extended Data Fig. 4).

### Influence of disturbance characteristics and environmental gradients

We calculated partial dependence plots (PDPs) for our models to assess how disturbance characteristics and environmental gradients influenced the habitat selection of disturbed and undisturbed forest areas.

**Fig. 2:**
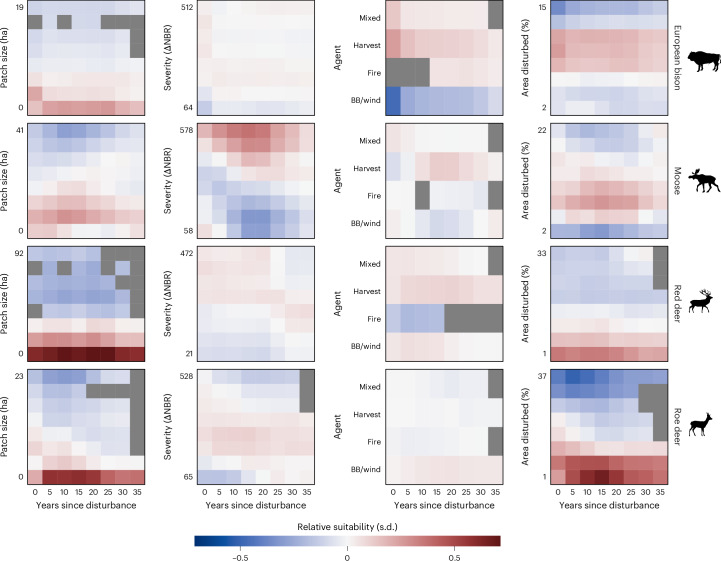
Influence of disturbance characteristics on relative habitat suitability across time since disturbance. The effects were estimated based on PDPs of habitat selection models. Disturbance severity reflects the difference in the normalized burn ratio (NBR), averaged at the scale of disturbance patches. PDPs were centred by subtracting the average suitability per year since disturbance to focus on the mediating effects of disturbance characteristics. Suitability was standardized based on forest habitat suitability in the year 2000 to facilitate comparisons across species. The *y* axis ranges vary across species due to the different environmental extents of tracking datasets. Value combinations not represented in our dataset are masked to avoid extrapolating models to empty data space and are displayed in grey colour. BB, bark beetle. Animal silhouettes from PhyloPic under a Creative Commons license: *C. elaphus* and *C. capreolus*, Ferran Sayol (CC0 1.0); *A. alces*, xgirousxb (PDM 1.0); *B. bonasus*, Steven Traver (PDM 1.0).

All species showed a stronger selection of smaller disturbance patches (with a relative effect on suitability of up to 0.3–0.7 standard deviations across species), although roe deer and European bison selected considerably smaller disturbance patches than moose and red deer (Fig. 2). In addition, all species selected disturbed areas more strongly when the disturbance severity was high (that is, more of the forest canopy was removed; the relative effect on suitability was up to 0.2–0.7 standard deviations). The occurrence of disturbance agents varied strongly in our dataset, with harvests being the most common agent and fire disturbances being particularly rare (37–79 versus 0.1–1.4% of all disturbances within the tracking data extents per species, respectively; Supplementary Fig. 1). PDPs mostly did not indicate clear differences among disturbance agents. Even so, European bison showed a weaker selection for areas disturbed by bark beetles and/or wind compared with other agents, whereas red deer showed a weaker selection for fire disturbances (Fig. 2). The selection for disturbed areas was also influenced by their availability (with a relative effect on suitability of up to 0.5–1.0 standard deviations): red deer and roe deer selected disturbed areas most strongly when they were rare within their home range, whereas moose and European bison showed the strongest selection given an intermediate availability.

**Extended Data Fig. 5:**
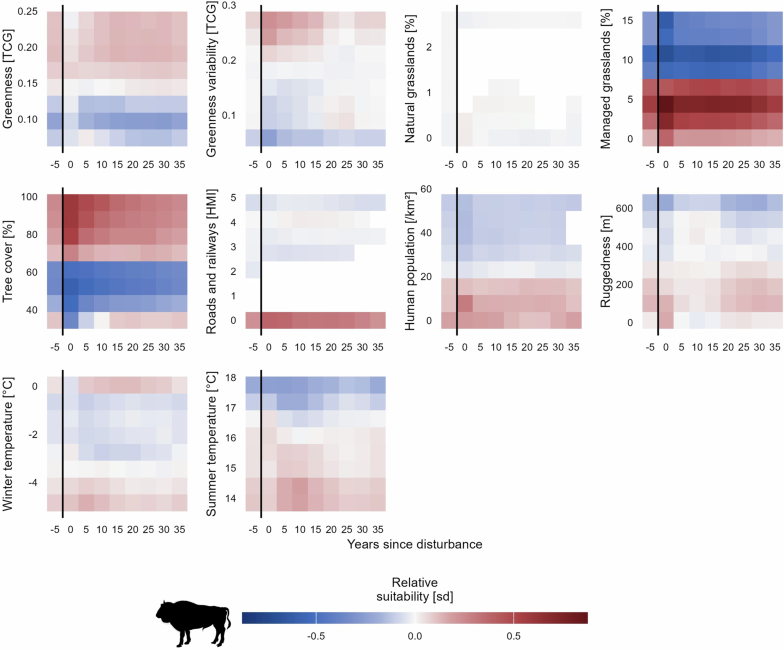
Influence of environmental variables on relative habitat suitability for European bison across time since disturbance. Effects are estimated based on PDPs of habitat selection models. PDPs were centred by subtracting the average suitability per year since disturbance value to focus on the mediation effect of environmental variables. Values of -5 on the X-axis correspond to undisturbed forest areas. Suitability was standardized based on forest habitat suitability in the year 2000 to facilitate comparisons across species. Value combinations not represented in our dataset are masked to avoid extrapolating models. TCG = tasseled cap greenness (vegetation index). HMI = human modification index (range: 1-100). Animal silhouettes from PhyloPic under a Creative Commons license: *C. elaphus* and *C. capreolus*, Ferran Sayol (CC0 1.0); *A. alces*, xgirousxb (PDM 1.0); *B. bonasus*, Steven Traver (PDM 1.0).

**Extended Data Fig. 6:**
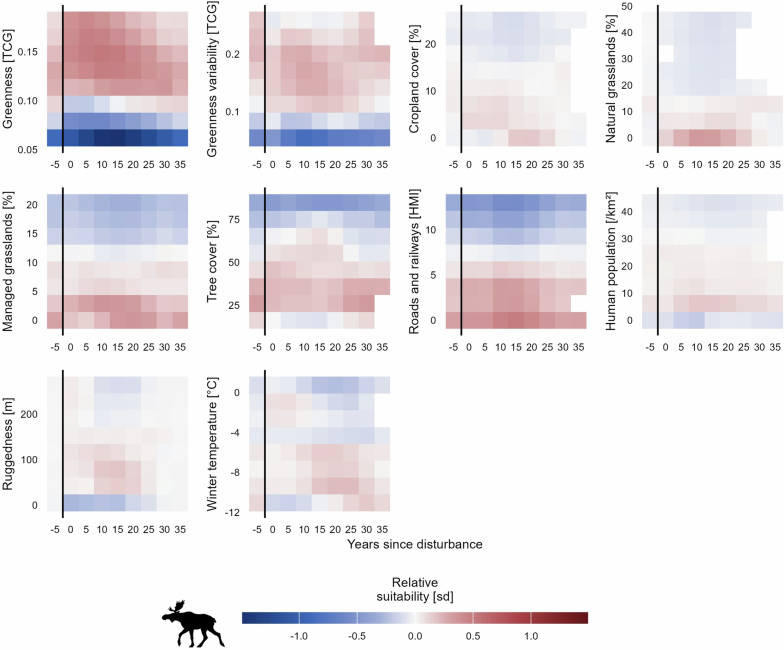
Influence of environmental variables on relative habitat suitability for moose across time since disturbance. Effects are estimated based on PDPs of habitat selection models. PDPs were centred by subtracting the average suitability per year since disturbance value to focus on the mediation effect of environmental variables. Values of -5 on the X-axis correspond to undisturbed forest areas. Suitability was standardized based on forest habitat suitability in the year 2000 to facilitate comparisons across species. Value combinations not represented in our dataset are masked to avoid extrapolating models. TCG = tasseled cap greenness (vegetation index). HMI = human modification index (range: 1-100). Animal silhouettes from PhyloPic under a Creative Commons license: *C. elaphus* and *C. capreolus,* Ferran Sayol (CC0 1.0); *A. alces*, xgirousxb (PDM 1.0); *B. bonasus*, Steven Traver (PDM 1.0).

**Extended Data Fig. 7:**
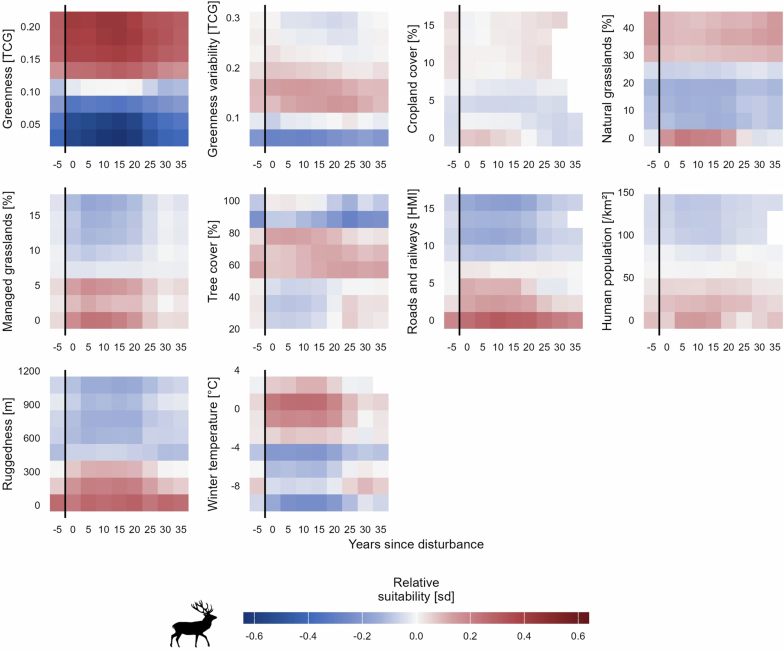
Influence of environmental variables on relative habitat suitability for red deer across time since disturbance. Effects are estimated based on PDPs of habitat selection models. PDPs were centred by subtracting the average suitability per year since disturbance value to focus on the mediation effect of environmental variables. Values of -5 on the X-axis correspond to undisturbed forest areas. Suitability was standardized based on forest habitat suitability in the year 2000 to facilitate comparisons across species. Value combinations not represented in our dataset are masked to avoid extrapolating models. TCG = tasseled cap greenness (vegetation index). HMI = human modification index (range: 1-100). Animal silhouettes from PhyloPic under a Creative Commons license: *C. elaphus* and *C. capreolus,* Ferran Sayol (CC0 1.0); *A. alces*, xgirousxb (PDM 1.0); *B. bonasus*, Steven Traver (PDM 1.0).

**Extended Data Fig. 8:**
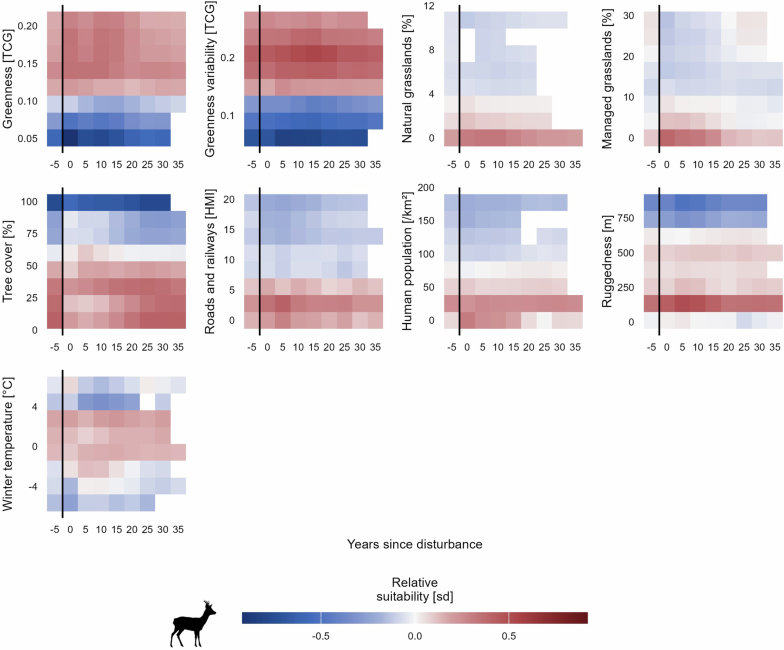
Influence of environmental variables on relative habitat suitability for roe deer across time since disturbance. Effects are estimated based on PDPs of habitat selection models. PDPs were centred by subtracting the average suitability per year since disturbance value to focus on the mediation effect of environmental variables. Values of -5 on the X-axis correspond to undisturbed forest areas. Suitability was standardized based on forest habitat suitability in the year 2000 to facilitate comparisons across species. Value combinations not represented in our dataset are masked to avoid extrapolating models. TCG = tasseled cap greenness (vegetation index). HMI = human modification index (range: 1-100). Animal silhouettes from PhyloPic under a Creative Commons license: *C. elaphus* and *C. capreolus,* Ferran Sayol (CC0 1.0); *A. alces*, xgirousxb (PDM 1.0); *B. bonasus*, Steven Traver (PDM 1.0).

In most cases, the responses to environmental variables were similar in recently disturbed versus undisturbed forest areas (Extended Data Figs. 5–8). The suitability of both disturbed and undisturbed forest areas strongly depended on local environmental characteristics and landscape composition (with relative effects on suitability of up to 1.1–2.0 standard deviations), with consistently negative responses to low vegetation greenness, high levels of human pressure and highly rugged terrain (Supplementary Note 1 provides a detailed summary of environmental effects). Red deer, moose and roe deer selected recently disturbed areas more strongly when the availability of other open habitat types (that is, croplands and/or grasslands) within their home range was very low. European bison showed the strongest selection for recently disturbed areas in landscapes with high tree cover and a low availability of grasslands.

**Extended Data Fig. 9:**
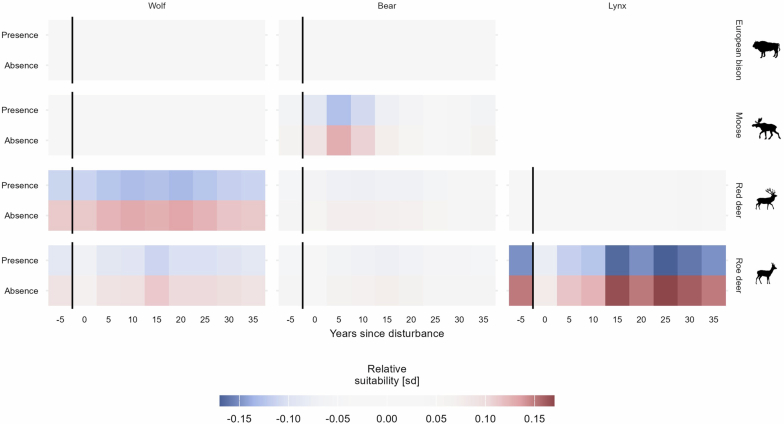
Effects of carnivore presence on relative habitat suitability across time since disturbance. Effects are estimated based on PDPs of habitat selection models. PDPs were centred by subtracting the average suitability per year since disturbance value to focus on the mediation effect of carnivore presence. Carnivore presence was derived from continent-wide surveys conducted at 10 km resolution. Values of -5 on the X-axis correspond to undisturbed forest areas. Suitability was standardized based on forest habitat suitability in the year 2000 to facilitate comparisons across species. Lynx presence was not used in the models for European bison and moose since lynx do not prey upon these species. Animal silhouettes from PhyloPic under a Creative Commons license: *C. elaphus* and *C. capreolus,* Ferran Sayol (CC0 1.0); *A. alces*, xgirousxb (PDM 1.0); *B. bonasus*, Steven Traver (PDM 1.0).

Broad-scale indicators of carnivore presence derived from distribution surveys of wolf (*Canis lupus*), brown bear (*Ursus arctos*) and Eurasian lynx (*Lynx lynx*) generally had weak influence on the selection of recently disturbed areas (Extended Data Fig. 9). Carnivore presence was associated with lower suitability for all species except European bison (with relative effects on suitability of <0.1 standard deviations), with the strongest negative effects of bear presence on moose, wolf presence on red deer and lynx presence on roe deer (with relative effects of up to 0.3 standard deviations in all cases). According to PDPs, the relative effect of carnivore presence tended to be weakest in recently disturbed areas and highest 5–25 years after the disturbance.

### Habitat suitability dynamics due to forest disturbances

We carried out a second, counterfactual prediction to assess how recent forest disturbance dynamics have contributed to habitat suitability dynamics for each study species. Specifically, we projected models across the species’ current and potential range extents for the period 2000–2023 and kept all variables unrelated to forest disturbance dynamics (for example, climate, land cover, human pressure and carnivore presence) at 2023 conditions. We used this approach to isolate and quantify the contribution of forest disturbance to habitat suitability dynamics. Models used for these projections were built without information on time since disturbance to avoid truncation bias due to a lack of information on forest disturbances before 1986.

**Fig. 3:**
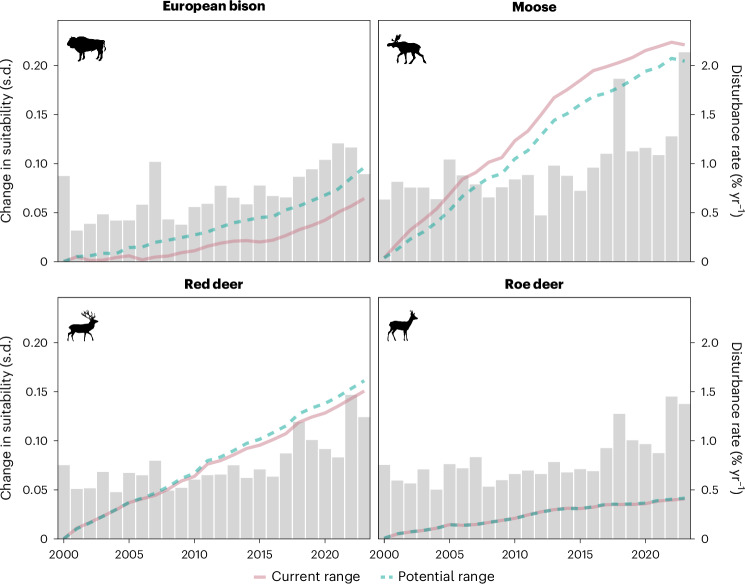
Forest disturbance effects on large herbivore habitat suitability across Europe between 2000 and 2023. Solid and dashed lines indicate habitat suitability changes across the species’ current and potential range extents, respectively. Predictions of fine-scale habitat suitability were derived for a stratified sample of disturbed and undisturbed forest pixels covering the current and potential range extents of species. Environmental variables unrelated to forest disturbances were kept constant to isolate the effects of disturbance dynamics on habitat suitability. To aid comparability, habitat suitability changes are expressed as relative changes and suitability values are standardized based on forest habitat suitability in the year 2000. The bar plots linked to the right plot axis show annual forest disturbance rates within species’ current ranges. For roe deer, the trend lines are overlapping due to the current and potential range extents being almost identical. Animal silhouettes from PhyloPic under a Creative Commons license: *C. elaphus* and *C. capreolus*, Ferran Sayol (CC0 1.0); *A. alces*, xgirousxb (PDM 1.0); *B. bonasus*, Steven Traver (PDM 1.0).

According to model projections, forest disturbances improved habitat suitability for all four large herbivore species between 2000 and 2023 across their current and potential ranges (Fig. 3). Disturbance-induced improvements in habitat suitability across the species’ current ranges were highest for moose (0.75 standard deviations within disturbed areas and 0.22 standard deviations across all forest areas), followed by red deer (0.88 and 0.15), European bison (0.49 and 0.06) and roe deer (0.24 and 0.04). Disturbance rates (that is, the percentage of forest area disturbed per year) across the current ranges were highest for moose (mean = 0.97% yr^−1^), followed by roe deer (0.81% yr^−1^), red deer (0.76% yr^−1^) and European bison (0.70% yr^−1^) (Fig. 3). Habitat suitability dynamics for all species showed similar patterns whether considering current or potential range extents.

**Fig. 4:**
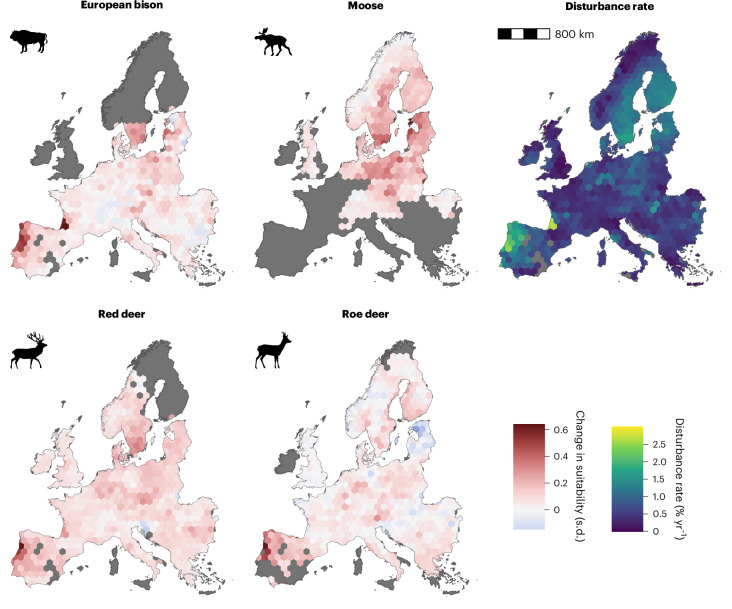
Spatial variation in habitat suitability changes caused by forest disturbance for four large herbivores in Europe between 2000 and 2023. Predictions for fine-scale habitat suitability changes are summarized at a scale of 8,860 km^2^ hexagons to visualize regional variation. Predictions were limited to potential species ranges based on the PHYLACINE database^112^. *C. elaphus corsicanus* was not included in the prediction. The top right panel shows spatial variation in disturbance rates (that is, the average percentage of forest annually disturbed per hexagon). Hexagons with <1,000 km^2^ of land area or <5% of forest are masked to ensure robust interpretation of suitability trends. Country shapefile data from GADM under a Creative Commons license CC BY-SA 2.0. Animal silhouettes from PhyloPic under a Creative Commons license: *C. elaphus* and *C. capreolus*, Ferran Sayol (CC0 1.0); *A. alces*, xgirouxb (PDM 1.0); *B. bonasus*, Steven Traver (PDM 1.0).

Model projections revealed considerable regional variation in disturbance-related habitat suitability dynamics (Fig. 4). Increases in habitat suitability for all species were most pronounced across Fennoscandia, the western Iberian Peninsula and parts of Central Europe, and generally corresponded to regions experiencing the highest forest disturbance rates between 2000 and 2023. Among grid cells covering potential ranges, 98, 94, 85 and 75% experienced habitat suitability increases due to forest disturbance for red deer, moose, European bison and roe deer, respectively. Among grid cells covering current ranges, the strongest increases in habitat suitability (calculated as changes across all forest areas) were 0.80, 0.57, 0.45 and 0.36 standard deviations for moose, red deer, roe deer and European bison, respectively.

## Discussion

Despite the strong links between forest disturbances, vegetation dynamics and large herbivores, it remains poorly understood how forest disturbances impact large herbivore habitats across species, seasons and over long time periods. Providing a broad-scale and long-term assessment of forest disturbance effects on large herbivore habitat selection, we reveal overall positive and long-lasting effects on habitat suitability for the four study species. Yet, disturbance responses showed considerable variation with local environmental conditions, landscape composition and disturbance characteristics. Our findings suggest that habitat selection of disturbed forest areas is species specific and primarily governed by differences in feeding ecology, highlighting the importance of disturbed areas as key foraging habitats for large herbivores. Our model projections across current and potential range extents indicate that the recent increase in forest disturbances considerably improved habitat conditions for large herbivores in Europe. This highlights the underappreciated role of forest disturbance as a key driver of wildlife habitat dynamics. Considering these impacts in both wildlife and forest management will be important given the widespread intensification of forest disturbance regimes.

The large herbivore responses to forest disturbances we found align well with differences in feeding ecology among species. The marked selection of recently disturbed areas during the growing season by red deer, roe deer and European bison is in line with higher consumption of non-browse vegetation during summer by these species^17,41^. In contrast, moose selected recently disturbed areas most intensively during winter, matching the species’ strong preference for browsing in young forests during winter^42^. Differences in the duration of habitat suitability improvements among browsing species (that is, moose, red deer and roe deer) could be explained by relative differences in browsing height, making older and thus larger trees available to moose but not to other species^43^. However, the more persistent use of disturbed areas may partly also be attributable to slower forest regeneration in areas inhabited by moose (that is, higher-latitude forests). The response of European bison, with a clear peak in habitat suitability in the first few years after a disturbance event, matches well with its preference for grazing and browsing on pioneer woody species such as *Rubus* species^41^, whereas the continued selection beyond this initial peak may reflect a preference for relatively low and open forest stands^44,45^. Thus, our results suggest that foraging preferences are the main factor driving habitat selection of disturbed forest areas and highlight their importance as important foraging habitats for large herbivores.

Our analysis covered the largest native strict herbivores with potential natural ranges spanning much of Europe, reflecting a large gradient in dietary niches^17^. Despite considerable intraspecific variation and decreases in habitat suitability after disturbance in some cases, we found overall positive responses by all species, in line with previous local assessments^15,46^. Yet, we caution that some large herbivores with a strong dependence on mature forests may respond negatively to forest disturbances (for example, reindeer (*Rangifer tarandus*), depending on undisturbed forest for lichens as key food items during winter^47^). Local studies from North America found varying responses across species, disturbance types and seasons^48–50^, whereas forest disturbance impacts on large herbivores in other forested biomes remain understudied^51,52^. Thus, more broad-scale and multi-species assessments are needed to better understand how large herbivores respond to forest disturbances in other regions.

Our dataset covering large parts of the species’ distributions in Europe allowed us to uncover important sources of variation in disturbance responses, including local environmental conditions, landscape composition and disturbance characteristics. All species preferred smaller disturbance patches. This is in line with findings for other large herbivores (for example, white-tailed deer (*Odocoileus virginianus*) in North America)^53^. In addition to a more direct accessibility of vegetation cover, smaller disturbance patches also typically offer better foraging conditions due to slower maturation and improved forage quality (lower carbon:nitrogen ratio)^53^. Thus, small disturbance patches create easily accessible edge habitats^46^, which may be the best solution to the forage–cover trade-off for many large herbivores^22^. Disturbances in our dataset were dominated by harvests and bark beetle or wind disturbances, accounting for more than 90% of disturbance events across species. We observed relatively little variation in habitat selection across disturbance agents, such as the relative avoidance of wildfire-related disturbances by red deer, matching previous findings^54^, and bark beetle and wind disturbances by European bison. However, we caution that our dataset was not well suited for characterizing varying impacts of disturbance agents due to frequent post-disturbance management in European forests^33^. Since standing and lying deadwood resulting from natural disturbances is commonly removed, agent-specific impacts may be masked by management and thus underestimated by our analysis. Post-disturbance management interventions such as salvage logging or tree planting can influence forage availability, vegetation cover and movement barriers created by deadwood^55^. To better understand the effects of post-disturbance management and disentangle the impacts of disturbance agents and associated characteristics, studies focusing on unmanaged disturbances (for example, in protected areas) or integrating fine-scale datasets on post-disturbance management measures are needed^18^.

Our models indicated strong variation across environmental gradients and overall similar responses by species inside disturbed and undisturbed forest areas. This suggests that large herbivore selection of disturbed forest areas is strongly influenced by more broad-scale selection patterns determining the suitability of forest areas more generally. We found that responses to environmental variables in our study matched well with previous findings on habitat selection by our study species (for example, the avoidance of habitats characterized by low vegetation productivity, high human pressure and highly rugged terrain; Supplementary Note 1). Variables capturing landscape composition were also important predictors in our models. For example, all species selected recently disturbed areas more strongly when the availability of other open habitat types (that is, grasslands and/or croplands) was very low. These patterns highlight plasticity in large herbivore use of disturbed forest areas in relation to the landscape context, with selection depending on the availability of alternative foraging habitats.

Due to the continental and multi-decadal extent of our analysis, we could not assess the fine-scale effects of some drivers that are probably important in mediating the suitability of disturbed areas for large herbivores. We found only weak associations between broad-scale distribution maps for large carnivores and habitat selection by our study species (that is, relative effects on habitat suitability of up to 0.3 versus up to 2.0 standard deviations for other variables). Our models indicated negative effects of carnivore presence on large herbivore habitat suitability, with the strongest relative effects being bear presence on moose, wolf presence on red deer and lynx presence on roe deer. These are plausible patterns given the expected dominant predator–prey relationships between these pairs of species^56–58^. According to our models, carnivore effects were weaker in recently disturbed areas and strongest ~5–25 years after disturbance, which may point towards increased predation risk and decreased selection associated with densely vegetated re-growing stands^59^. Available studies suggest variable impacts of carnivore presence on large herbivore use of disturbed forest areas^59–61^, which may further interact with effects of hunting^62^. Thus, studies integrating fine-scale datasets from carnivores and herbivores across different ecological contexts are needed to gain more robust insights into the effects of predation risk. Human pressure is an important stressor and source of mortality for many large herbivores, often having larger effects on large herbivore space use than predation risk by natural predators^63^. Our models indicated consistently negative responses to high levels of human pressure in disturbed and undisturbed forest areas by all species (Extended Data Figs. 5–8). However, due to the lack of suitable reference datasets, our analysis could not provide insights into specific human pressures acting within forests, such as hunting, logging or recreational activities, which can all influence the selection of disturbed forest areas by large herbivores^22,64,65^.

Our model projections across the species’ range extents indicate that recent forest disturbance dynamics across Europe had positive effects on habitat suitability for large herbivores, with considerably stronger improvements in habitat suitability for moose and red deer than for European bison and roe deer. Projected improvements were regionally variable, generally coinciding with regions that experienced the highest forest disturbance rates (that is, Fennoscandia, Western Iberia and parts of Central Europe). Therefore, the particularly strong habitat suitability improvements observed for moose not only reflect a strong selection of disturbed areas by this species, but also particularly high disturbance rates across its current and potential range. The disturbance-driven improvements in habitat suitability documented by our work are important to consider in the management and conservation of European large herbivores. Previous studies have established links between forest disturbance dynamics and large herbivore performance or population trends^46,66^. Habitat suitability improvements caused by forest disturbances across Europe may result in higher potential carrying capacities and even population increases, although demographic responses will further depend on density-dependent processes, management and mortality risks. Recently published data on country-level trends in hunting bags for European large herbivores (including moose, red deer and roe deer) suggest overall increasing population trends over recengt decades, albeit with considerable variation across regions and species^34^. More research integrating information on potential drivers such as climate change, land use dynamics and forest disturbance dynamics is needed to understand their relative importance for large herbivore population dynamics.

In addition, our findings underscore that at smaller scales disturbed areas have a long-lasting attraction effect on large herbivores. This can trigger increased top-down effects of herbivory on forest vegetation in disturbed areas compared with closed-canopy forests^23^ and can amplify conflicts with forestry due to increased browsing damages^15^. However, at the same time, habitat improvements can contribute to restoration goals aiming to boost large herbivore effects on forest vegetation, which can help to maintain greater openness inside European forests, with potential benefits for biodiversity and ecosystem resilience^25,26^. In this context, the largest European herbivore, the European bison, may play an important role due to its strong effects on forest regeneration^39^. Although management strategies focused on timber production versus ecosystem restoration are often in conflict in this regard, forest management strategies that mimic natural gap dynamics may help to balance different management goals, as they restore forest heterogeneity, promote less concentrated browsing and improve habitat conditions for large herbivores and many other species^15,67^.

Although our results indicate that current levels of forest disturbance in Europe are beneficial for all four study species, it is important to consider potential negative effects if disturbance rates continue to increase due to climate change (for example, due to increased insect outbreaks, fires or windthrows) or intensified forest use. All species responded negatively to increasing disturbance patch size and high levels of disturbed areas inside their home ranges. These patterns suggest that further increases in disturbance levels, particularly in the form of large disturbance patches, could eventually result in net-negative impacts on habitat suitability for large herbivores. Our findings highlight a strongly attraction effect of disturbed areas, and the impacts of large herbivores on vegetation are often particularly strong inside forest gaps^23^. However, we caution that the relationship between disturbance dynamics and browsing pressure on vegetation may be complex. For example, increases in the availability of disturbed forest due to higher disturbance rates may result in less concentrated foraging patterns and thus reduced average browsing pressure inside disturbed areas.

Here, we focused on predicting habitat dynamics within forest habitats, but understanding the effects of landscape dynamics outside forests would be important for obtaining a complete picture of large herbivore habitat dynamics. Land use dynamics in non-forest habitats, such as agricultural abandonment^68^, also resulted in considerable habitat changes in Europe over recent decades^69^. Although we used a comprehensive dataset on forest disturbances, it came with some limitations. Since forest disturbance maps were only available at an annual temporal resolution, we could not eliminate uncertainty from cases in which disturbances happened in the months after a tracking location was recorded. In addition, due to the limited temporal extent of the dataset, we were only able to characterize habitat dynamics up to 35 years after disturbance. Analyses that integrate regional datasets on forest age structure, which were not available for the fine spatial resolution and continental extent we worked with here, may provide further insights into habitat suitability dynamics inside older forest stands. Finally, projecting our models across the potential range extents of species required considerable extrapolation in some cases, particularly for European bison. Although we found highly similar trends in habitat suitability between current and potential range extents and cross-validations suggested very good transferability of our models, we caution that such model extrapolations are associated with higher levels of uncertainty^70^. Our study demonstrates the unique potential of combining big animal tracking and satellite time series data for understanding wildlife habitat dynamics and underscores the central role of forest disturbances as a driver of large herbivore habitat suitability. Despite strong recent increases in forest disturbances across many regions of the globe, forest disturbance impacts on wildlife often remain overlooked in the management and conservation of wildlife and forests. Here we show that large herbivores in Europe have benefitted from recent increases in disturbance rates. Importantly, natural forest disturbances are expected to further increase as a result of climate change^10^, whereas increased wood demand (for example, for energy production) may further increase anthropogenic disturbances^71^. Given the important role of large herbivores in shaping forest ecosystems, a deeper understanding of the interactions between forest disturbance dynamics and large herbivores is needed to inform adaptive management and conservation strategies.

## Methods

### Overview of animal tracking data

We collected global positioning system tracking data from 228 European bison across 14 study sites, 768 moose across 21 study sites, 924 red deer across 39 study sites and 1,149 roe deer across 31 study sites (Extended Data Fig. 1). Our datasets comprise large parts of all four species’ current ranges in Europe and were collected between 1997 and 2023 (Extended Data Fig. 2). In total, our data contained 19,305,552 raw locations.

### Pre-processing of animal tracking data

In a first processing step, we grouped animals into species-wise environmental clusters. This was done to allow the balancing of datasets during model training and validation and thereby account for environmental sampling bias present in our dataset. We used size-constraint clustering to avoid highly uneven cluster sizes^72,73^, defining the minimum cluster size as one-eighth of the tracked animals per species. As clustering variables, we used forest cover, ruggedness, winter temperature and human population density (Supplementary Note 2 provides details).

We removed potentially erroneous locations following the automated approach by ref. ^74^. We filtered the remaining tracking data in two steps. First, to focus on habitat selection within stationary home ranges, we removed periods during which animals travelled unusually large distances, probably corresponding to non-stationary movement behaviours (for example, exploratory movements, dispersal or seasonal migration). We calculated weekly net squared displacement (NSD) values^75^ per animal, aggregated weekly NSD values per environmental cluster and then removed weeks corresponding to the highest 10% of NSD values per cluster. Second, we sub-sampled the tracking data to reduce autocorrelation between locations^76^. We tested the correlation of step lengths for different time lags based on animals with high sampling rates (that is, <2 h). To ensure independence of observations and avoid biases associated with 24-h behavioural rhythms^77^, we chose a conservative 7-h minimum sampling interval, exceeding the ~2-h decay in autocorrelation observed in our data (Supplementary Fig. 2 provides details). This step removed 87, 57, 88 and 86% of the remaining tracking locations for European bison, moose, red deer and roe deer, respectively.

### Use-availability design

We created a use-availability design for building habitat selection models^76^. Our goal was to characterize the selection of habitats within home ranges (that is, third-order habitat selection^78^) while also assuming nearby, probably reachable habitats to be available to animals. To characterize varying habitat availability for animals shifting their home ranges throughout the year (for example, due to seasonal migration^79,80^), we split locations per animal into tracks and calculated approximate home ranges at the track level. Specifically, we split locations into separate tracks if there were gaps of 7 or more days between locations (for example, due to weeks with migration activity that had been removed earlier on in the analysis). We removed tracks shorter than 30 d or with fewer than 30 locations to ensure robust home range estimates. This led to the removal of 12 animals from our dataset, leaving 3,057 for modelling. Then, we used 95% minimum convex polygons (MCPs) to calculate approximate home ranges per track. We used MCPs as a home range estimator since our goal was not to identify exact home ranges but to define areas for background sampling that liberally characterize habitat availability^81,82^. The median sizes of combined home ranges per animal (that is, the spatial union of MCPs across all tracking periods) were 68.2, 39.6 , 6.7 and 6.7 km^2^ for European bison, moose, red deer and roe deer, respectivly. To define areas available to an animal, we buffered each home range by one-quarter of the diameter of a hypothetical circular home range with the same size (average buffer sizes = 1.7 km for European bison, 1.2 km for moose, 0.6 km for red deer and 0.3 km for roe deer). We used this approach to account for varying home range sizes and movement behaviours across individuals and tracking periods. Within these buffered home ranges, we sampled five random locations per tracking location as background points, assigning the recording time of tracking locations to the background points^18^.

### Balancing tracking locations across environmental clusters

We balanced the presence–background datasets across environmental clusters to avoid biasing models towards environmental conditions with more available data. Since our dataset comprised large numbers of individuals per species, but the number of individuals and tracking locations varied strongly across environmental clusters, we considered this environmental bias the primary source of bias for our models. Therefore, we downsampled data at the animal level so that the number of locations was the same across clusters. This allowed us to keep data from as many individuals as possible, thus enhancing species-level representativeness^83^ while balancing the contribution of different environmental clusters and individual animals within them^84,85^. The final datasets for training habitat selection models contained 113,531 locations for European bison, 397,920 locations for moose, 427,658 locations for red deer and 709,553 locations for roe deer (Table 1).

### Predictors of habitat selection

We used a time-calibrated modelling approach in which time series of environmental variables are temporally matched with animal occurrence records to capture wildlife responses to landscape dynamics^18^. We generated predictor variables at two spatial scales: (1) a local scale of 30 m × 30 m (the pixel size of our satellite-based variables); and (2) a home-range scale, which varied by species. We derived home-range scale predictors by applying moving window averaging to the raster datasets^85^, using window sizes reflecting our median home-range sizes per species. Adding home-range scale predictor variables allowed our models to learn how habitat selection varied with habitat availability, reflecting broader-scale selection processes (that is, home-range placement). Considering the variation of habitat selection due to varying resource availability across different landscape contexts (so-called functional responses in habitat selection^86^) is particularly important in analyses covering large environmental gradients^85,87^. We used four types of predictors for training our habitat selection models (Extended Data Table 1). First, we added month and sex to account for potential variation in habitat selection throughout the year and between sexes. Second, we included variables characterizing forest disturbances. Third, we added variables describing other habitat conditions (forest mask, topography, climate, human pressure and land cover). Fourth, we used broad-scale indicators of large carnivore presence.

We used forest disturbance maps derived from Landsat satellite time series to characterize disturbance dynamics^31^. The maps indicate stand-replacing disturbances between 1986 and 2023 at an annual level and 30 m spatial resolution and further contain information on disturbance characteristics. We derived five variables from these maps: years since disturbance; probable disturbance agent (classified as bark beetle/windthrow, fire, harvest or mixed; derived from an attribution algorithm^31^); the size of disturbance patches; disturbance severity (calculated as the average decrease in the normalized burn ratio index per disturbance patch^88^); and the area of recently disturbed forest (≤10 years since disturbance) at the scale of animal home ranges. Since the disturbance maps covered the period of 1986–2023, the maximum potential value for the years-since-disturbance variable was 37 years. We assigned a constant value of −100 to non-forest areas to help our models discriminate between varying habitat selection in disturbed versus undisturbed forest areas and forest versus non-forest areas.

As additional indicators of post-disturbance vegetation dynamics, we used spectral–temporal metrics derived from tasselled cap transformations of Landsat reflectance^18,89^: median greenness and the seasonality of greenness (inter-decile range). Satellite-based greenness indicators can be useful proxies for forage availability in open habitats^90^, whereas the seasonality of greenness is a useful indicator for deciduous vegetation^91^—a potentially important driver of forage site selection by large herbivores^42,92^. Landsat-based spectral–temporal metrics are further useful to capture variation in forest structure in mature forest stands^93^. To minimize the influence of year-to-year variability in satellite data availability, we used 3-year moving windows to calculate metrics and applied the temporal segmentation algorithm LandTrendr^94^ to smooth the time series (ref. ^18^ provides details on the approach).

We included nine variables describing other environmental habitat conditions. To further discriminate habitat selection between non-forest, undisturbed forest and disturbed forest areas, we added a categorical predictor containing these classes derived from our forest disturbance maps^31^. We used the topographic ruggedness index to capture movement constraints created by rugged terrain^95^. As climatic variables, we included the mean temperatures during the coldest and warmest quarters to indicate the exposure to seasonal thermal extremes, which can imply a higher energy demand for thermoregulation^19^. As proxies of human pressure, we used human population density and transportation infrastructure (that is, roads and railways). Finally, we included tree cover, cropland cover and the cover of natural and managed grasslands derived from global land cover products^96,97^. We calculated the availability of land cover types at the scale of animal home ranges to characterize landscape composition (that is, the relative proportion of land cover types in neighbourhoods corresponding to the species’ home range sizes), including the availability of alternative foraging habitats outside forests (that is, croplands and grasslands).

We included distribution maps indicating the (permanent) presence of grey wolf, Eurasian lynx and brown bear to capture potential effects of predator presence on habitat selection. Since disturbed forest areas may be associated with higher predation risk^20^, large herbivores could avoid them in regions occupied by predators. Carnivore distribution maps were based on European-wide surveys carried out for three periods (2006–2011, 2012–2016 and 2017–2022) at 10-km spatial resolution^98–100^ (Supplementary Fig. 3 provides an overview of the carnivore distribution maps used in our study). We did not use lynx presence in the models for European bison and moose since lynx do not typically prey on these species. Our habitat selection models included 21 predictors for red deer and roe deer and 20 predictors for European bison and moose (Extended Data Table 1 provides a full overview and data sources). We removed predictors with pairwise correlations of >0.7 (ref. ^101^), retaining variables that we deemed more important for our analysis. This led to removal of the cropland cover variable for bison and roe deer (highly correlated with forest cover) and the mean summer temperature variable for moose, red deer and roe deer (highly correlated with mean winter temperature). We used the Google Earth Engine^102^ to process predictor variables.

### Parametrization of habitat selection models

We built habitat selection models using random forests^103^, which are well suited for capturing nonlinear responses and predictor interactions in large animal tracking datasets^104,105^. Since class imbalances can negatively affect random forest model performance, we used downsampled random forests^106^. In this approach, a subsample of background locations is used to balance both classes (presence and background) within each tree of the model, whereas the full set of background points is used during model training. As random forest parameters, we used 300 trees and tuned two key hyperparameters (the number of variables selected per split and the maximum depth of trees; Supplementary Note 3, Supplementary Fig. 4 and Supplementary Table 1). After hyperparameter tuning, we assessed the transferability of our models based on a cross-validation with environmental clusters as folds^107^, which indicated excellent predictive performance and transferability of our models (mean continuous Boyce indices = 0.99, 0.98, 0.91 and 0.89 for European bison, moose, red deer and roe deer, respectively). We used the R package randomForest^108^ to build habitat selection models.

### Interpretation of model outputs

Given the use-availability design underlying our models, the predictions by our models indicate the relative intensities of habitat selection^109^. Furthermore, assuming that habitat selection reflects informed decisions of animals to maximize their fitness, model predictions can be interpreted as expected fitness payoffs^40^. We thus follow the common interpretation of model predictions as indicators of relative habitat suitability, but acknowledge that our models do not account for changes in habitat selection due to varying population densities^40,110^. To facilitate comparisons across species, we standardized habitat suitability predictions across species, since the absolute values of model predictions typically depend on the definition of habitat availability^40^. Specifically, we used the mean and standard deviation of suitability values across forest areas within species’ current range extents in the year 2000. Changes are therefore expressed in units of standard deviation relative to forest habitat suitability in the year 2000. We applied the same standardization for all model predictions (that is, for calculating PDPs, disturbance responses and suitability changes across Europe).

### PDPs

To assess how disturbance characteristics, carnivore presence and environmental conditions influenced habitat selection across different stages of forest development, we calculated PDPs. PDPs visualize the marginal effect of a predictor variable by showing how model predictions change as one or more variables vary while averaging out other predictors^111^. We built two-dimensional PDPs of years since disturbance and all other predictors. We used 5-year bins of years since disturbance and eight equally spaced bins for numeric predictors. For factor variables, one prediction was made per factor level. The PDPs allowed us to investigate interactive effects between post-disturbance forest development and other variables. To focus on the mediation effect of variables, we applied a group-wise centring of PDPs by subtracting the average predicted suitability per bin of years-since-disturbance values. The resulting dependence plots therefore show the relative influence of predictors on habitat suitability at different times since disturbance, whereas the amplitude of these mediation effects can be interpreted as the relative effect sizes. We masked bin combinations with <50 samples in our training data to avoid extrapolating models to environmental conditions not well supported by our data.

### Samples of disturbed and undisturbed forest areas

We created a stratified sample of disturbed and undisturbed forest areas to infer temporal patterns of habitat suitability changes after disturbance events and assess habitat suitability dynamics caused by recent disturbance dynamics across the species’ current and potential range extents. Potential range extents correspond to counterfactual natural ranges (that is, areas in which species would occur without human influence). For this, we created a grid of 100-km-wide hexagons (~8,660 km^2^ area) across our study area and sampled pixels from disturbed and undisturbed forest areas per grid cell. To avoid extrapolating our models to regions outside the potential ranges of species (for example, areas with unsuitable climate conditions), we limited the predictions of our models to grid cells that were covered at least 50% by potential range extents derived from the PHYLACINE database^112^. Furthermore, we combined distribution maps from ref. ^13^ with animal home ranges from our tracking dataset to delineate areas representing the current range extents of species (Supplementary Fig. 5 provides an overview of the distribution maps used for defining current and potential range extents). We considered grid cells covered at least 20% by a species’ distribution as covering the current range of a species. Due to its limited and fragmented current range, we did not apply this filtering step for the European bison.

To calculate the required sample sizes per stratum (that is, disturbed and undisturbed forest areas per hexagonal grid cell), we used the following formula:

$$n=\,\frac{N\times {Z}^{2}\times {\sigma }^{2}}{\left({(E\times {\rm{\mu }})}^{2}\times N\right)+({Z}^{\,2}\times {\sigma }^{2})}$$

where *n* is the designated sample size per stratum, *N* is the stratum size (that is, the number of cells belonging to the stratum), *Z* is the *Z* value corresponding to the desired confidence level, *σ*^2^ is the variance within the stratum (that is, the variance of suitability values), *E* is the allowed relative margin of error and *µ* is the stratum mean. We used a *Z* value of 1.96, corresponding to 95% confidence.

To estimate ${\mu }$ and $\sigma$ for the sampling strata, we used model predictions on our training datasets per class (that is, disturbed and undisturbed forest areas). To account for the fact that disturbances accumulate over time (that is, the stratum of disturbed areas is largely undisturbed at the beginning of the time series), we estimated ${\mu }$ and $\sigma$ for the stratum of disturbed forest areas as a weighted mean of class-wise model predictions, defining weights based on the distribution of disturbance years. In total, our samples (covering current and potential ranges) comprised 1,419,991 pixels for European bison, 558,656 pixels for moose, 1,055,612 pixels for red deer and 1,141,148 pixels for roe deer.

### Temporal responses to forest disturbance

To assess disturbance responses, we predicted our models across all samples from grid cells that overlapped with the current range extents of species. We made predictions for all months between 2000 and 2023, including predictions for both sexes, which we averaged to infer species-level responses. We did not back-cast habitat suitability further in time because we had little tracking data available from before 2000 (Table 1) and several predictors did not cover the period before the year 2000 (Extended Data Table 1). To summarize large herbivore responses to forest disturbances over time, we grouped predicted habitat suitability values chronologically based on the years since disturbances (hereafter, disturbance responses)^18^. We assessed seasonal variation by computing separate disturbance responses for each month. To focus on relative changes caused by disturbance, we calculated disturbance responses as the difference in predicted habitat suitability compared with the 5-year average before a disturbance.

### Changes in habitat suitability across current and potential range extents

To assess changes in habitat suitability caused by recent disturbance dynamics in Europe, we carried out a second model prediction across the current and potential range extents of species. For this prediction, we used separately trained models not containing information on the years since disturbance as a predictor variable. We excluded this variable to avoid a truncation bias due to a lack of information on disturbances happening before 1986. Instead, we added four additional predictors based on spectral–temporal metrics derived from tasselled cap transformations of Landsat reflectance (median wetness and brightness and inter-decile ranges of wetness and brightness). Thus, these models primarily leverage satellite-based metrics to characterize habitat suitability dynamics associated with forest disturbance, recovery and maturation^18^. We applied the same tuning and cross-validation approach described above, which again indicated excellent model transferability across environmental clusters (mean continuous Boyce indices from cross-validation = 0.98, 0.95, 0.94 and 0.88 for European bison, moose, red deer and roe deer, respectively). Disturbance responses obtained from models built without the years-since-disturbance variable were similar to those obtained from our original models (Supplementary Fig. 6).

To isolate the effect of forest disturbances on habitat suitability, we used a counterfactual prediction approach. Specifically, we kept all predictor variables unrelated to the forest disturbances static (with a target year of 2023) and predicted suitability trends across all sample pixels between 2000 and 2023. This meant that in undisturbed areas (that is, with no detected disturbance between 1986 and 2023), the only variable that varied across time was the area of recently disturbed forest, describing the number of pixels that were disturbed ≤10 years ago within neighbourhoods reflecting the species’ home range sizes. In areas with detected disturbances, all variables describing forest disturbance dynamics varied annually across the prediction time frame (patch size, disturbance agent, disturbance severity and the Landsat-based spectral–temporal metrics), whereas all others were kept static. To summarize trends across space and time, we calculated average habitat suitability values for each year and grid cell and across the current and potential range extents for all species. We used weighted means to calculate average values per grid cell and across range extents, weighting values by the number of disturbed or undisturbed pixels per grid cell. To visualize habitat suitability trends across space, we excluded all grid cells covering <1,000 km^2^ of land area (for example, cells located at the boundaries of our study area) or with less than five percent forest cover, to eliminate cells without a robust data basis for calculating habitat suitability trends.

### Reporting summary

Further information on research design is available in the Nature Portfolio Reporting Summary linked to this article.

## Supplementary information

Supplementary Information Supplementary Figs. 1–6, Notes 1–3, and Table 1.

Reporting Summary

Peer Review File

## Figures and Tables

**Extended Data Table 1:**
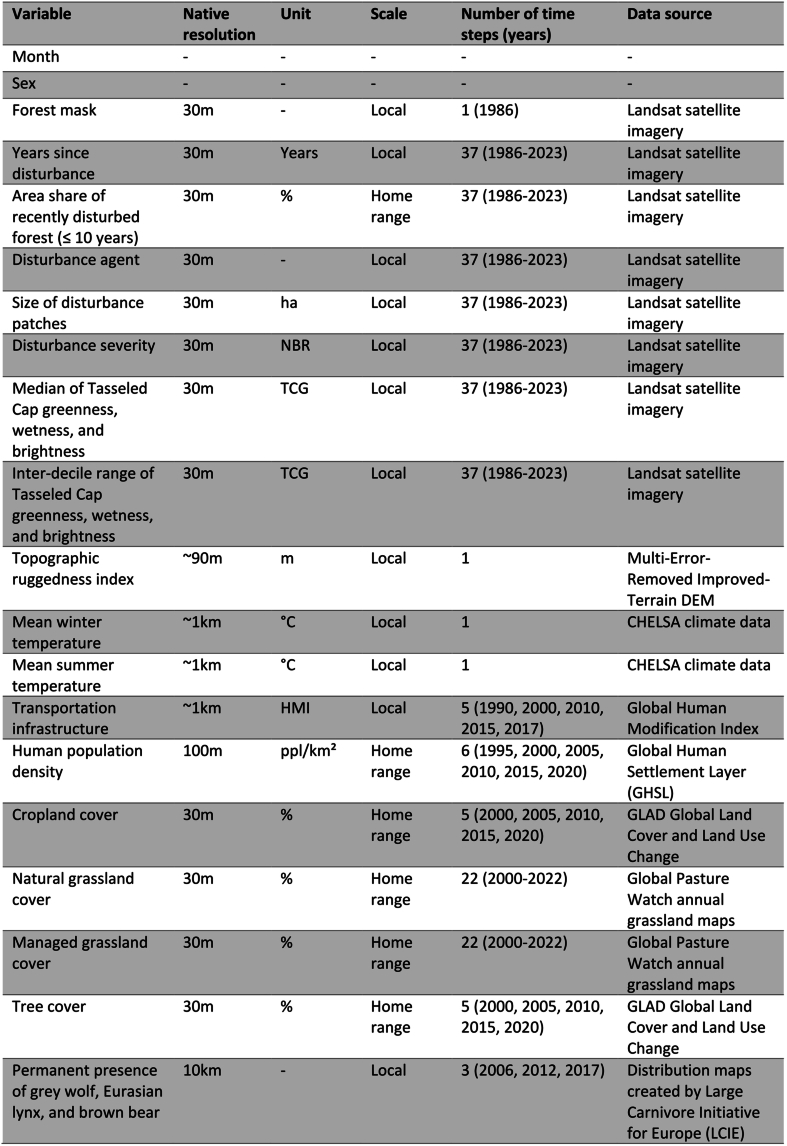
Overview of predictor variables used in habitat selection models

Scale refers to the spatial scale at which variables were included in the models. Local corresponds to a 30 m scale, and home range corresponds to the average home range size per species in our dataset.

## Data Availability

Training data for building habitat selection models are available from figshare at 10.6084/m9.figshare.30296536 (ref. ^113^).
