# Supplementary material for: Increasing forest disturbance enhances habitat suitability for Europe’s large herbivores
Source: Nat Ecol Evol. 2026 Jun 26;10(7):1273–86. doi: 10.1038/s41559-026-03096-0 (PMC13345911; doi:10.1038/s41559-026-03096-0)
Supplement: Supplementary file 1 — Supplementary Figs. 1–6, Notes 1–3, and Table 1. [file 41559_2026_3096_MOESM1_ESM.pdf]

nature ecology & evolution

Supplementary information https://doi.org/10.1038/s41559-026-03096-0

Increasing forest disturbance enhances habitat suitability for Europe's large herbivores

In the format provided by the authors and unedited

Supplementary Figure 1

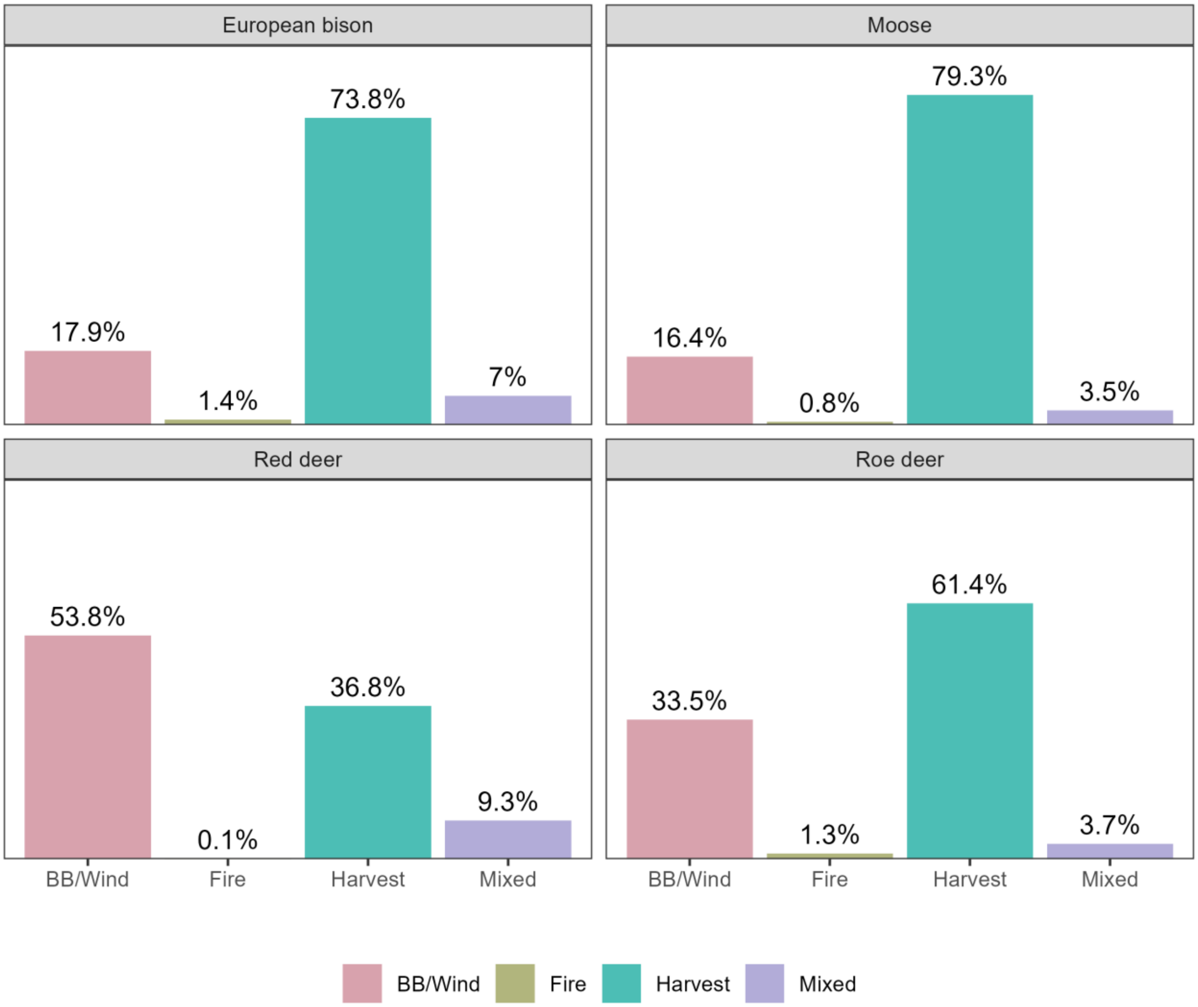

Supplementary Fig. 1: *Relative frequency of disturbance agents within areas covered by the tracking datasets per species. Agent classification was derived from the forest disturbance maps underlying our analysis.*

Supplementary Figure 2

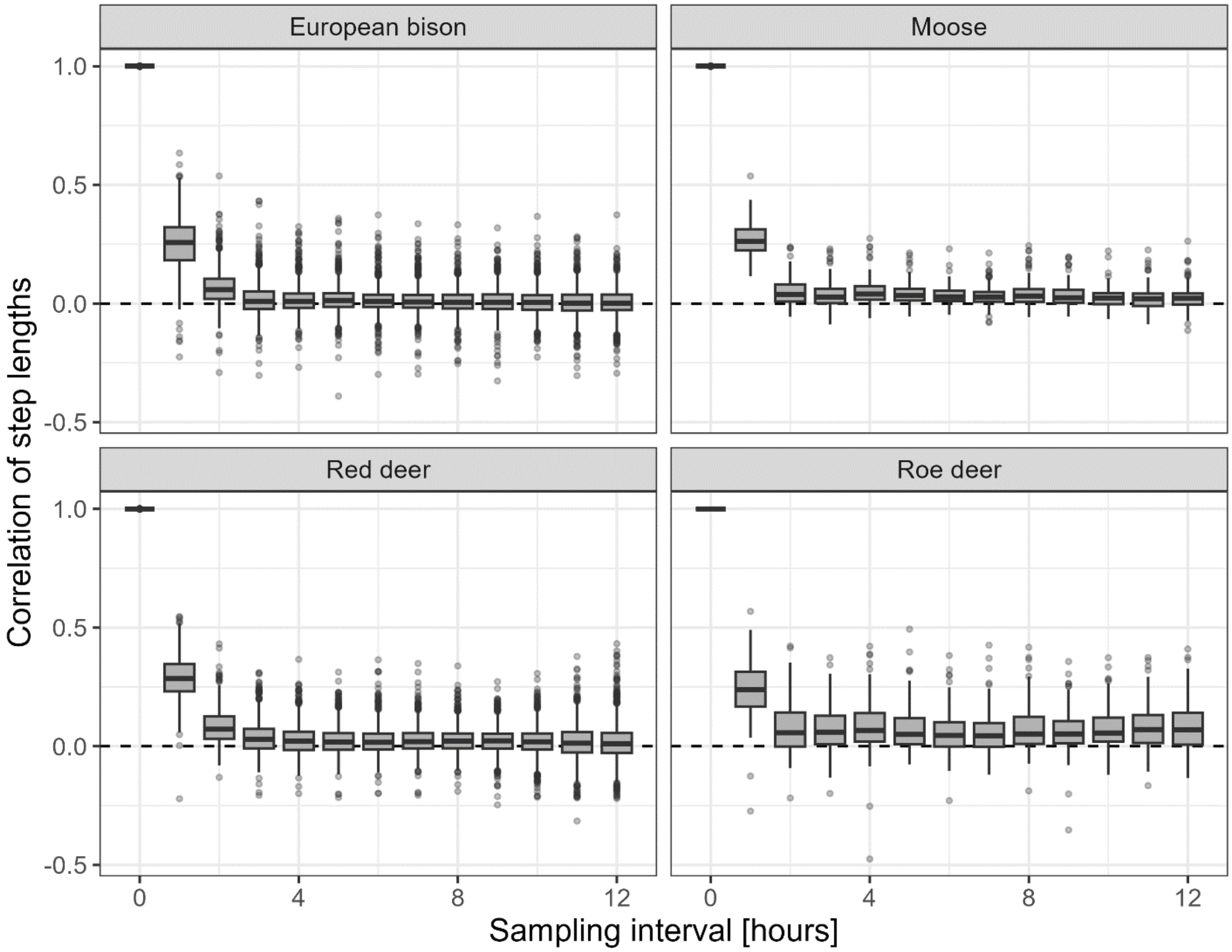

***Supplementary Fig. 2**: Relationship between sampling intervals and the correlation of step lengths for the four large herbivores. Based on animals with high available sampling intervals (i.e., <2 hours between fixes), we tested how sampling intervals impacted the spatiotemporal autocorrelation of consecutive tracking locations by calculating correlations of step lengths (i.e., Euclidean distances between consecutive locations). Results indicated strongly decreasing correlation beyond two hours for all species.*

Supplementary Figure 3

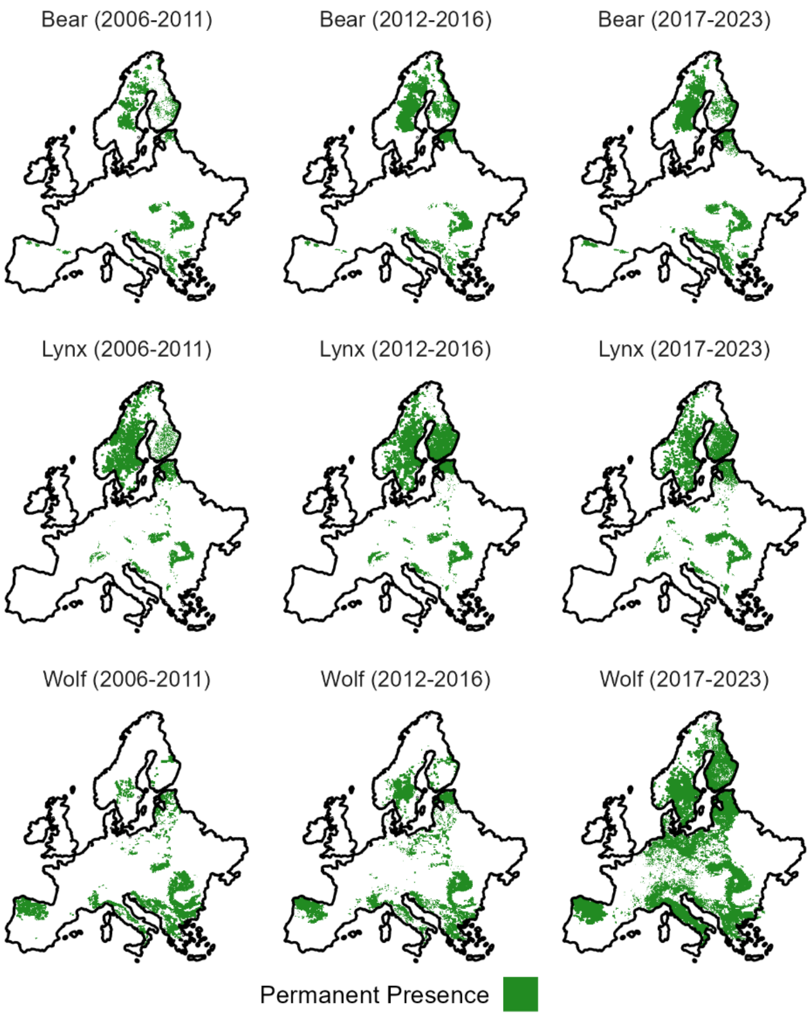

Supplementary Fig. 3*: Carnivore distribution maps used as predictors in habitat selection models. The underlying data was obtained from three cycles of continent-wide surveys conducted at 10km resolution (https://doi.org/10.5061/dryad.3xsj3txrc, https://doi.org/10.5061/dryad.pc866t1p3, https://doi.org/10.5061/dryad.986mp) and limited to grid cells identified as "permanent presence".*

Supplementary Figure 4

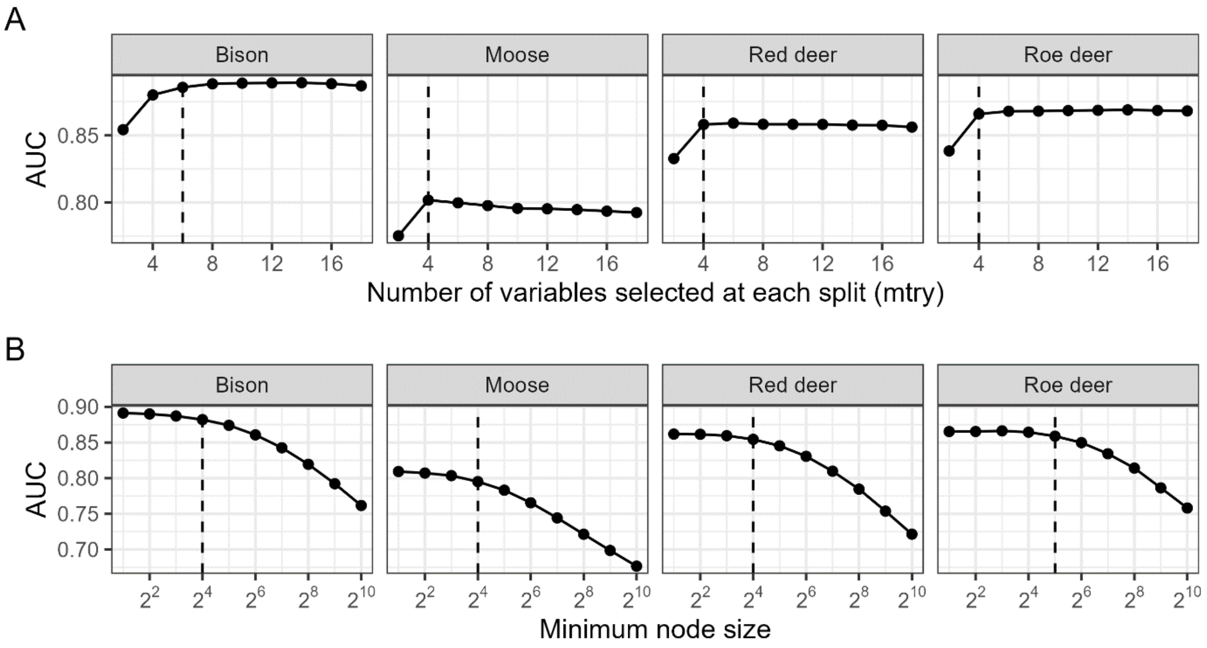

Supplementary Fig. 4*: Performance curves for two random forest hyperparameters. A: number of variables selected at each split (mtry). B: minimum node size. Dotted lines indicate selected parameter settings at which performance was within one standard error of the highest performance recorded for the parameter. We evaluated models based on out-of-bag AUC values, balancing datasets across disturbed/undisturbed forest areas and environmental clusters for validation.*

Supplementary Figure 5

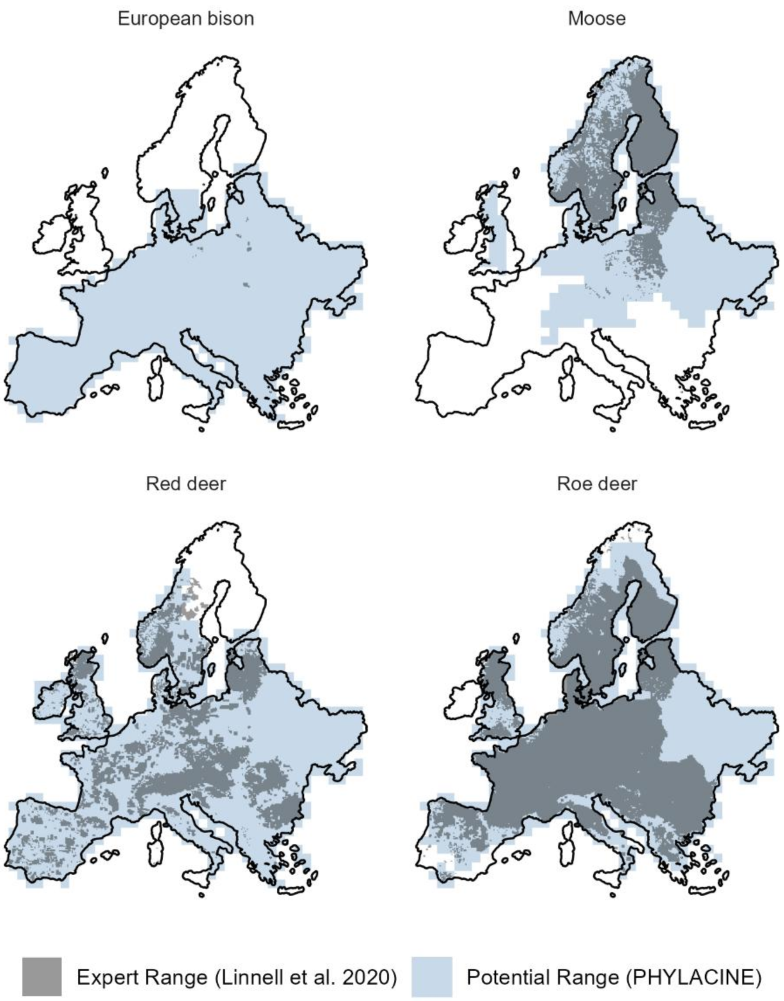

Supplementary Fig. 5*: Distribution maps used for identifying current and potential range extents of species. For defining current range extents, we combined expert range maps from Linnell et al. 2020 (paper: https://doi.org/10.1016/j.biocon.2020.108500; data source: https://doi.org/10.17605/OSF.IO/N5P2U) with animal home ranges derived from our tracking datasets. Potential range extents were derived from the database PHYLACINE 1.2 (https://doi.org/10.5061/dryad.bp26v20).*

Supplementary Figure 6

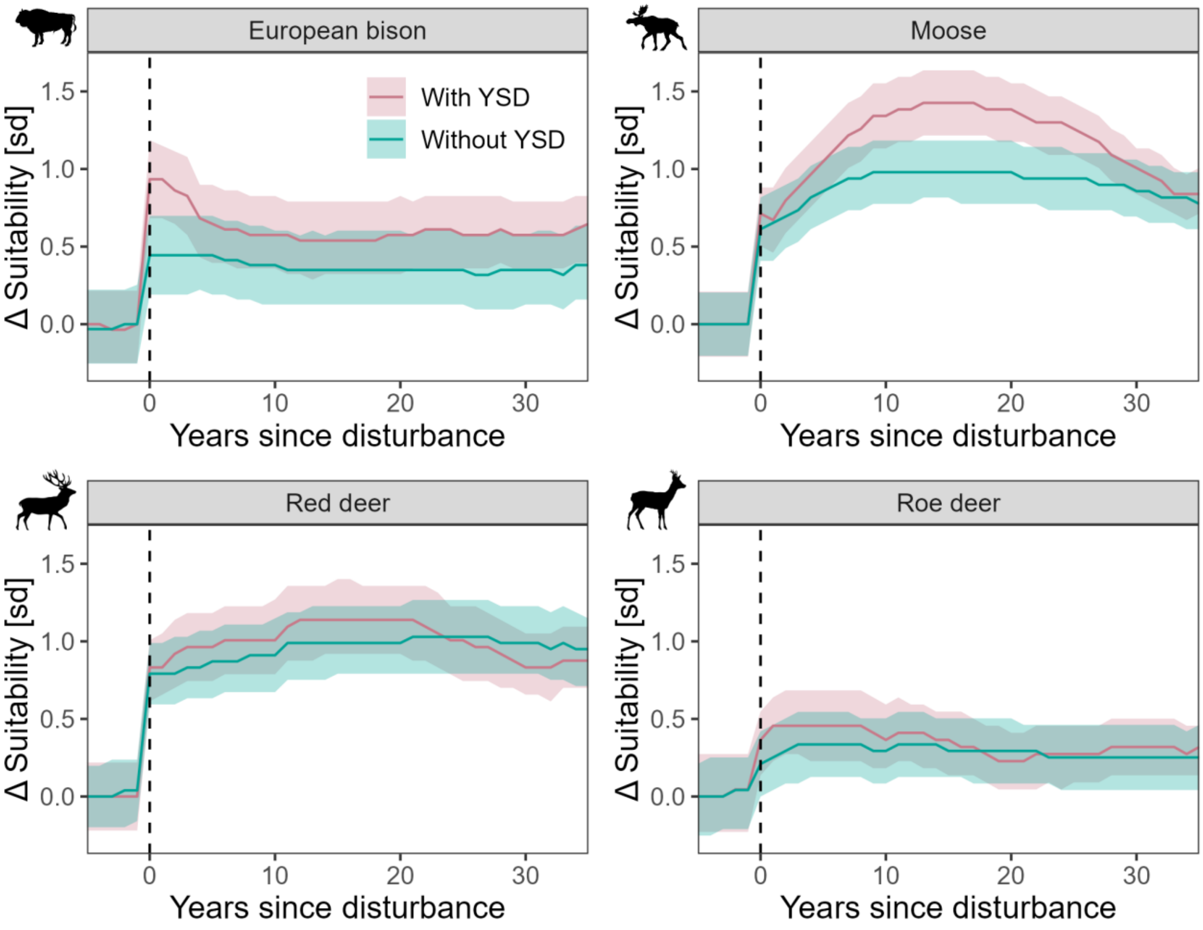

Supplementary Fig. 6*: Comparison of disturbance responses obtained from habitat selection models built with and without years since disturbance (YSD) as a predictor. Disturbance responses are shown as relative changes in standardized habitat suitability compared to the five-year average prior to a disturbance event, where standardization is based on the distribution of forest habitat suitability in the year 2000 Colored lines indicate median suitability, and the colored ribbons the 40th to 60th percentile ranges per model. Models built without the YSD variable were used to assess forest disturbance impacts on habitat suitability across the species' range extents in order to avoid a truncation bias due to a lack of information on forest disturbance happening prior to 1986 (see Methods section in the main text for details).*

Supplementary Note 1

Variables derived from the satellite vegetation index Tasseled Cap greenness (i.e., average greenness and greenness seasonality) and variables summarizing the composition of landscapes at the scale of

species' home ranges (i.e., tree cover, managed/unmanaged grassland cover) had the strongest impacts on the relative suitability of both disturbed and undisturbed forest areas (relative effect sizes based on partial dependence plots of up to 0.5-2.0 standard deviations for greenness variables, and 0.4-1.6 standard deviations for landscape composition variables across species). All species showed generally positive responses to higher average greenness, likely reflecting a general preference for more productive habitats with higher forage availability[1] (Extended data Figs. 5-8).

For all species, the availability of tree cover played an important role. Roe deer, moose, and – to a lesser degree – red deer selected forest areas (i.e., including undisturbed and disturbed areas) less strongly when their availability was high (Extended data Figs. 6-8). In addition, red deer selected recently disturbed areas (up to ca. 20 years post-disturbance) less strongly in open landscapes with low tree cover (Extended data Fig. 7). In contrast to other species, European bison showed a strong selection of forest areas, and particularly recently disturbed areas, in landscapes with high available tree cover (i.e., densely forested landscapes; Extended data Fig. 5). These patterns indicate that the fine-scale selection of disturbed areas is strongly influenced by broader-scale selection patterns (i.e., the placement of home ranges within the wider landscape), which determine whether disturbed areas are available as foraging resources within the home range[2]. Our results indicate considerable plasticity in the use of forests by our study species[3] and highlight the importance functional responses in habitat selection[4] (i.e., adjustments of selection based on habitat availability).

In addition to tree cover, the availability of alternative foraging habitats (i.e., grasslands and croplands) were important in determining habitat selection by all species. Patterns for European bison may reflect a strongly opportunistic and facultative use of disturbed forest areas as foraging habitats, showing the strongest selection for recently disturbed areas in landscapes with a low availability of grasslands and high tree cover (i.e., a lack of potentially more suitable alternative foraging habitats; Extended data Fig.5). Similarly, roe deer, red deer, and moose showed an increased selection for recently disturbed areas when the availability of grasslands and/or croplands was very low. While for moose and red deer, this effect was mostly linked to the availability of natural grasslands, for roe deer and European bison the availability of managed grasslands had a larger relative effect. This could suggest that these species can utilize grasslands and croplands for satisfying some forage demands (e.g., for grasses or forbs) and compensate a lack of open habitats through an increased use of disturbed forest areas.

All species showed a decreased selection of forest areas associated with high levels of human pressure (i.e., high values of the variables human population density and roads and railways; Extended data Figs. 5-8), likely reflecting a broad-scale avoidance of highly transformed habitats[5,6] (e.g., urban areas). European bison, red deer and roe deer showed a relative avoidance of forests located in highly rugged terrain, while moose did not occur in mountainous areas featuring high ruggedness (Extended data Figs. 5-8). The avoidance of highly rugged areas can be explained by high movement costs[7]. Considering climatic gradients, recently disturbed areas were selected less strongly in regions with high summer temperatures by European bison (Extended Data Fig.5), and in regions with low winter temperatures by red and roe deer (Extended Data Figs. 7-8). These patterns may reflect poorer forage availability inside disturbed areas in colder climates, also affected by snow cover, as well a reduced selection of open habitats and stronger dependence on vegetation cover because of a higher exposure to thermal extremes[8].

Supplementary Note 2

We clustered animals into environmental clusters per species based on a sample of tracking locations of up to 1000 points per animal. As clustering variables, we used a subset of our environmental predictors that characterize land cover, climate, topography, and human pressure (forest cover, mean temperature of the coldest quarter, terrain ruggedness, and human population density). To further reduce dimensionality, we applied a principal component analysis (PCA) and used the first two components as input variables for clustering. These PC components explained 97% of the observed variance in clustering variables for European bison, 99% for moose, 99% for red deer, and 98% for roe deer. Then, we applied size-constrained clustering using the R package *scclust*[9]. We defined the minimum cluster size as ⅛ of animals available per species and used the first two components of our PCA analysis for calculating distances between data points (i.e., animals). The algorithm implemented in the *scclust* package clusters data points using a graph-based approach to minimize within-cluster dissimilarities while ensuring that each cluster contains at least a certain number of points.

Supplementary Note 3

We used out-of-bag (OOB) predictions for tuning random forest hyperparameters, which helps to avoid re-fitting models multiple times for each parameter setting[10]. OOB predictions are calculated during model training as predictions by a tree on samples that were not used during its training. We used the area under the receiver operating characteristic curve (AUC) as a metric for measuring predictive performance. To weigh predictive performance in disturbed and undisturbed forest areas equally, we

used a balanced sample containing the same number of locations per class for validation. In addition, we calculated separate AUC values per environmental cluster (see Methods section in the main text and Supplementary Note 2). To ensure the stability of performance scores, we repeated the sampling of validation points five times per environmental cluster before aggregating AUC values across clusters.

We tuned two hyperparameters that are considered among the most influential for random forest performance[10]: the number of variables to randomly sample as candidates at each split (*mtry*) and the minimum node size, which controls the smallest number of observations required for a node to be split further during tree construction. For *mtry*, we tested values between 2 and 18 (increments of 2). For the minimum node size, we tested powers of two between 2 and 1024. We tuned parameters sequentially, first choosing the optimal *mtry* value before tuning the minimum node size parameter. To avoid overfitting models, we applied the one-standard-error rule for selecting optimal hyperparameter settings[11]. Specifically, we chose parameter values corresponding to the least complex model (i.e., lowest *mtry* and highest minimum node size) for which performance was within one standard error of the maximum performance score recorded per parameter (Supplementary Fig. 4). For an overview of selected hyperparameters, see Table Supplementary Table 1.

Supplementary Table 1

Supplementary Table 1*: Selected hyperparameters.*

Species	Variables selected at each split	Minimum node size
European bison	6	16
Moose	4	16
Red deer	4	16
Roe deer	4	32

References

1. Rempfler, T. *et al.* Remote sensing reveals the role of forage quality and quantity for summer habitat use in red deer. *Mov Ecol* **12**, 80 (2024).

2. Retez, G. *et al.* Habitat preferences of European bison in contemporary European landscapes. *Journal of Applied Ecology* **63**, e70233 (2026).

3. Spitzer, R. *et al.* Fifty years of European ungulate dietary studies: a synthesis. *Oikos* **129**, 1668–1680 (2020).

4. Mysterud, A. & Ims, R. A. Functional Responses in Habitat Use: Availability Influences Relative Use in Trade-Off Situations. *Ecology* **79**, 1435–1441 (1998).

5. Müller, A., Dahm, M., Bøcher, P. K., Root-Bernstein, M. & Svenning, J.-C. Large herbivores in novel ecosystems - Habitat selection by red deer (Cervus elaphus) in a former brown-coal mining area. *PLOS ONE* **12**, e0177431 (2017).

6. Eldegard, K., Lyngved, J. T. & Hjeljord, O. Coping in a human-dominated landscape: trade-off between foraging and keeping away from roads by moose (Alces alces). *Eur J Wildl Res* **58**, 969–979 (2012).

7. Killeen, J. *et al.* Habitat selection during ungulate dispersal and exploratory movement at broad and fine scale with implications for conservation management. *Mov Ecol* **2**, 15 (2014).

8. Mysterud, A. & Østbye, E. Cover as a Habitat Element for Temperate Ungulates: Effects on Habitat Selection and Demography. *Wildlife Society Bulletin (1973-2006)* **27**, 385–394 (1999).

9. Savje, F., Higgins, M. & Sekhon, J. scclust: Size-Constrained Clustering. (2024).

10. Probst, P., Wright, M. N. & Boulesteix, A.-L. Hyperparameters and tuning strategies for random forest. *WIREs Data Mining and Knowledge Discovery* **9**, e1301 (2019).

11. Hastie, T., Friedman, J. & Tibshirani, R. Model Assessment and Selection. in *The Elements of Statistical Learning: Data Mining, Inference, and Prediction* (eds Hastie, T., Friedman, J. & Tibshirani, R.) 193–224 (Springer, New York, NY, 2001). doi:10.1007/978-0-387-21606-5_7.
