# Supplementary material for: Increasing forest disturbance enhances habitat suitability for Europe’s large herbivores
Source: Nat Ecol Evol. 2026 Jun 26;10(7):1273–86. doi: 10.1038/s41559-026-03096-0 (PMC13345911; doi:10.1038/s41559-026-03096-0)
Supplement: Supplementary file 2 — Reporting Summary [file 41559_2026_3096_MOESM2_ESM.pdf]

nature portfolio

Corresponding author(s): Julian Oeser

Last updated by author(s): Mar 19, 2026

Reporting Summary

Nature Portfolio wishes to improve the reproducibility of the work that we publish. This form provides structure for consistency and transparency in reporting. For further information on Nature Portfolio policies, see our Editorial Policies and the Editorial Policy Checklist.

Statistics

For all statistical analyses, confirm that the following items are present in the figure legend, table legend, main text, or Methods section.

n/a	Confirmed	
☐	☒	The exact sample size (*n*) for each experimental group/condition, given as a discrete number and unit of measurement
☐	☒	A statement on whether measurements were taken from distinct samples or whether the same sample was measured repeatedly
☒	☐	The statistical test(s) used AND whether they are one- or two-sided<br>*Only common tests should be described solely by name; describe more complex techniques in the Methods section.*
☐	☒	A description of all covariates tested
☒	☐	A description of any assumptions or corrections, such as tests of normality and adjustment for multiple comparisons
☐	☒	A full description of the statistical parameters including central tendency (e.g. means) or other basic estimates (e.g. regression coefficient) AND variation (e.g. standard deviation) or associated estimates of uncertainty (e.g. confidence intervals)
☒	☐	For null hypothesis testing, the test statistic (e.g. *F*, *t*, *r*) with confidence intervals, effect sizes, degrees of freedom and *P* value noted<br>*Give P values as exact values whenever suitable.*
☒	☐	For Bayesian analysis, information on the choice of priors and Markov chain Monte Carlo settings
☒	☐	For hierarchical and complex designs, identification of the appropriate level for tests and full reporting of outcomes
☒	☐	Estimates of effect sizes (e.g. Cohen's *d*, Pearson's *r*), indicating how they were calculated

Our web collection on statistics for biologists contains articles on many of the points above.

Software and code

Policy information about availability of computer code

Data collection	No software was used.
Data analysis	Data analysis was performed in R (4.5.1). Habitat selection models were built with the package randomForest (4.7-1.2).

For manuscripts utilizing custom algorithms or software that are central to the research but not yet described in published literature, software must be made available to editors and reviewers. We strongly encourage code deposition in a community repository (e.g. GitHub). See the Nature Portfolio guidelines for submitting code & software for further information.

Data

Policy information about availability of data

All manuscripts must include a data availability statement. This statement should provide the following information, where applicable:

- Accession codes, unique identifiers, or web links for publicly available datasets
- A description of any restrictions on data availability
- For clinical datasets or third party data, please ensure that the statement adheres to our policy

Data used for building habitat selection models are available in a Figshare data repository: https://doi.org/10.6084/m9.figshare.30296536

Research involving human participants, their data, or biological material

Policy information about studies with human participants or human data. See also policy information about sex, gender (identity/presentation), and sexual orientation and race, ethnicity and racism.

Reporting on sex and gender	*Use the terms sex (biological attribute) and gender (shaped by social and cultural circumstances) carefully in order to avoid confusing both terms. Indicate if findings apply to only one sex or gender; describe whether sex and gender were considered in study design; whether sex and/or gender was determined based on self-reporting or assigned and methods used. Provide in the source data disaggregated sex and gender data, where this information has been collected, and if consent has been obtained for sharing of individual-level data; provide overall numbers in this Reporting Summary. Please state if this information has not been collected. Report sex- and gender-based analyses where performed, justify reasons for lack of sex- and gender-based analysis.*
Reporting on race, ethnicity, or other socially relevant groupings	*Please specify the socially constructed or socially relevant categorization variable(s) used in your manuscript and explain why they were used. Please note that such variables should not be used as proxies for other socially constructed/relevant variables (for example, race or ethnicity should not be used as a proxy for socioeconomic status). Provide clear definitions of the relevant terms used, how they were provided (by the participants/respondents, the researchers, or third parties), and the method(s) used to classify people into the different categories (e.g. self-report, census or administrative data, social media data, etc.) Please provide details about how you controlled for confounding variables in your analyses.*
Population characteristics	*Describe the covariate-relevant population characteristics of the human research participants (e.g. age, genotypic information, past and current diagnosis and treatment categories). If you filled out the behavioural & social sciences study design questions and have nothing to add here, write "See above."*
Recruitment	*Describe how participants were recruited. Outline any potential self-selection bias or other biases that may be present and how these are likely to impact results.*
Ethics oversight	*Identify the organization(s) that approved the study protocol.*

Note that full information on the approval of the study protocol must also be provided in the manuscript.

Field-specific reporting

Please select the one below that is the best fit for your research. If you are not sure, read the appropriate sections before making your selection.

☐ Life sciences ☐ Behavioural & social sciences ☒ Ecological, evolutionary & environmental sciences

For a reference copy of the document with all sections, see nature.com/documents/nr-reporting-summary-flat.pdf

Ecological, evolutionary & environmental sciences study design

All studies must disclose on these points even when the disclosure is negative.

Study description	This is a continental-scale, multi-species, and long-term assessment of how forest disturbances impact large herbivore habitat selection and suitability across Europe. It links animal tracking data (3,069 individuals) with satellite-derived time series of forest disturbance, climate, and human pressure variables. Animal tracking datasets were used to build habitat selection models per species. Then, these models were used to predict habitat suitability across a stratified sample of disturbed and undisturbed forest areas across Europe to infer species responses to forest disturbances as well as changes in forest habitat suitability across time.
Research sample	GPS tracking data from 228 European bison (Bison bonasus; 175 female/53 male), 768 moose (Alces alces; 592 female / 176 male), 918 red deer (Cervus elaphus; 685 female / 233 male), 1143 roe deer (713 female / 430 male)
Sampling strategy	The study utilized presence-background datasets to model habitat selection. To avoid bias, data were down-sampled at the animal level to ensure an equal number of locations across environmental clusters in the data. To calculate the required sample sizes for estimating habitat suitability in disturbed and undisturbed forest areas per hexagonal grid cell across Europe, we used a sample size formula for estimating a population mean with a relative margin of error.
Data collection	Tracking datasets were collected by the co-authors and by members of the EUROMAMMALS network (https://euromammals.org/).
Timing and spatial scale	Study extent: continental scale across Europe, covering the current and potential ranges of the four species.<br>Prediction scales: predictions of habitat selection / suitability were made at a spatial resolution of 30m grid cells, and sample pixels of disturbed and undisturbed forest pixels were aggregated at the level of 100 km-wide (8,660km²) hexagons.<br>Temporal scale: Tracking data were collected between 1997 and 2023.<br>Forest disturbance maps covered 1986-2023<br>Habitat suitability predictions covered the period 2000 to 2023.

Data exclusions	None of the available animal tracking datasets were excluded a priori from the analysis. Tracking datasets per animal were sub-sampled to reduce spatiotemporal autocorrelation and balance datasets across environmental clusters in the data. Tracks (consecutive GPS observations) with fewer than 30 observations were removed from the analysis to allow the calculation of home ranges (available habitats).
Reproducibility	Datasets for building habitat selection models are made available through a Figshare data repository. Throughout the development of the analytical workflow, habitat selection models were re-run several time to ensure the consistency of results.
Randomization	This is a large-scale, correlational study using remote sensing and animal tracking data; experimental groups were not allocated, so randomization of organisms/samples is not applicable
Blinding	This is a large-scale, correlational study using remote sensing and animal tracking data; blinding of researchers to samples or measurements is not applicable

Did the study involve field work? ☐ Yes ☒ No

Reporting for specific materials, systems and methods

We require information from authors about some types of materials, experimental systems and methods used in many studies. Here, indicate whether each material, system or method listed is relevant to your study. If you are not sure if a list item applies to your research, read the appropriate section before selecting a response.

Materials & experimental systems

n/a	Involved in the study
☒	☐ Antibodies
☒	☐ Eukaryotic cell lines
☒	☐ Palaeontology and archaeology
☐	☒ Animals and other organisms
☒	☐ Clinical data
☒	☐ Dual use research of concern
☒	☐ Plants

Methods

n/a	Involved in the study
☒	☐ ChIP-seq
☒	☐ Flow cytometry
☒	☐ MRI-based neuroimaging

Animals and other research organisms

Policy information about studies involving animals; ARRIVE guidelines recommended for reporting animal research, and Sex and Gender in Research

Laboratory animals	No laboratory animals were used for this study.
Wild animals	Animal tracking datasets used in this study comprise previously collected datasets aggregated across many study areas. Data from 228 European bison (Bison bonasus; 175 female/53 male), 768 moose (Alces alces; 592 female / 176 male), 918 red deer (Cervus elaphus; 685 female / 233 male), 1143 roe deer (713 female / 430 male) were collected using GPS collars. All captured animals were released after tagging.
Reporting on sex	For all species, tracking data from more females than males was available: 228 European bison (Bison bonasus; 175 female/53 male), 768 moose (Alces alces; 592 female / 176 male), 918 red deer (Cervus elaphus; 685 female / 233 male), 1143 roe deer (713 female / 430 male). Sex was accounted for in the habitat selection models by adding sex as a predictor to the random forest models. Sex-wise predictions of habitat selection models indicated that responses to forest disturbance did not clearly differ between sexes.
Field-collected samples	No laboratory work was done for this study.
Ethics oversight	All animal capture and tagging procedures were performed in strict compliance with the relevant national and institutional guidelines and legislations. The collection of animal tracking data was approved by the respective permit-issuing bodies for each study site across Europe.

Note that full information on the approval of the study protocol must also be provided in the manuscript.

Plants

Seed stocks	*Report on the source of all seed stocks or other plant material used. If applicable, state the seed stock centre and catalogue number. If plant specimens were collected from the field, describe the collection location, date and sampling procedures.*
Novel plant genotypes	*Describe the methods by which all novel plant genotypes were produced. This includes those generated by transgenic approaches, gene editing, chemical/radiation-based mutagenesis and hybridization. For transgenic lines, describe the transformation method, the number of independent lines analyzed and the generation upon which experiments were performed. For gene-edited lines, describe the editor used, the endogenous sequence targeted for editing, the targeting guide RNA sequence (if applicable) and how the editor was applied.*
Authentication	*Describe any authentication procedures for each seed stock used or novel genotype generated. Describe any experiments used to assess the effect of a mutation and, where applicable, how potential secondary effects (e.g. second site T-DNA insertions, mosiacism, off-target gene editing) were examined.*
