# Supplementary material for: Increasing forest disturbance enhances habitat suitability for Europe’s large herbivores
Source: Nat Ecol Evol. 2026 Jun 26;10(7):1273–86. doi: 10.1038/s41559-026-03096-0 (PMC13345911; doi:10.1038/s41559-026-03096-0)
Supplement: Supplementary file 3 — Peer Review File [file 41559_2026_3096_MOESM3_ESM.pdf]

nature portfolio

Peer Review File

Increasing forest disturbance enhances habitat suitability for Europe’s large herbivores

Corresponding Author: Dr Julian Oeser

Version 0:

Decision Letter:

19th November 2025

*Please ensure you delete the link to your author homepage in this e-mail if you wish to forward it to your co-authors.

Dear Dr Oeser,

Thank you for your patience while your manuscript entitled "Increasing forest disturbance enhances habitat suitability for Europe’s large herbivores" was under review. As you will see from the reports below, the reviewers find the work of interest but have some concerns. Therefore, we would like to see a revised manuscript along with a response to the reviewers' criticisms.

We are committed to providing a fair and constructive peer-review process. Please do not hesitate to contact me if you have any questions or there are requests from the reviewers that you believe are technically impossible or unlikely to yield a meaningful outcome.

When revising your manuscript:

* Please provide the manuscript in Microsoft Word format and highlight all changes in the same file or a copy.

* Please include the Figshare or other data and code repository links in the data and code availability statements in the main manuscript file.
* Include a “Response to reviewers” document detailing, point-by-point, how you addressed each reviewer comment. If no action was taken to address a point, you must provide a compelling argument. This response will be sent back to the reviewers along with the revised manuscript.

* If you have not done so already please begin to revise your manuscript so that it conforms to our Article format instructions at http://www.nature.com/natecolevol/info/final-submission. Refer also to any guidelines provided in this letter.

* Include a revised version of any required reporting checklist. It will be available to referees (and, potentially, statisticians) to aid in their evaluation if the manuscript goes back for peer review. A revised checklist is essential for re-review of the paper.

* Extended Data Figures - please ensure that any supplementary figures and tables that are crucial to the manuscript’s conclusions are converted into Extended Data figures and tables to increase visibility of these data. Extended Data figures and tables are online-only (present in the online PDF and full-text HTML versions of the paper), peer-reviewed display items that provide essential background to the article but are not included in the main article due to space constraints. A maximum of ten Extended Data display items (figures and tables) is permitted.

We hope to receive your revised manuscript within three months. If you cannot send it within this time, please let us know. We will be happy to consider your revision so long as nothing similar has been accepted for publication at Nature Ecology & Evolution or published elsewhere. Once ready, please use the link below to submit your revised manuscript and related files:

Link Redacted

Note: This URL links to your confidential home page and associated information about manuscripts you may have submitted, or that you are reviewing for us. If you wish to forward this email to co-authors, please delete the link to your

homepage.

Nature Ecology & Evolution is committed to improving transparency in authorship. As part of our efforts in this direction, we are now requesting that all authors identified as ‘corresponding author’ on published papers create and link their Open Researcher and Contributor Identifier (ORCID) with their account on the Manuscript Tracking System (MTS), prior to acceptance. ORCID helps the scientific community achieve unambiguous attribution of all scholarly contributions. You can create and link your ORCID from the home page of the MTS by clicking on ‘Modify my Springer Nature account’. For more information please visit please visit www.springernature.com/orcid.

We look forward to seeing the revised manuscript and thank you for the opportunity to review your work.

[redacted]

****

Reviewer expertise:

Reviewer #1: forest disturbance, remote sensing

Reviewer #2 and co-reviewers: mammalian community dynamics, wildlife monitoring

Reviewers' comments:

Reviewer #1 (Remarks to the Author):

Review for manuscript entitled: “Increasing forest disturbance enhances habitat suitability for Europe’s large herbivores” submitted Nature Ecology and Evolution.

In the manuscript the authors analyze forest disturbance effects on an extensive animal tracking dataset of four large herbivore species across Europe. They provide evidence that forest disturbance enhances habitat suitability for Europe’s large herbivores, an interesting finding. Aside from the modeling framework (on which I cannot comment, since I am not an animal movement ecologist) and thus would refer to an expert on the applicability of their methodology. I found several relatively major issues that need to be addressed before the paper can be published in Nature Ecology and Evolution. In summary, I think this is a worthwhile (enormous?!) effort with interesting findings that would be of interest to Nature Ecology and Evolution provided that the authors address the comments below and pending an expert review on the statistical methods performed.

Major comments:
- Increasing forest disturbances: A lot of the motivation of this paper relies on the premise that forest disturbances are increasing, however there is not a lot of recent citations that cover the current study area (Europe) that show this trend and why this is happening. Proof of this and corresponding citations might be available and it would be good for the authors to include this or build out this expected trend with proof, otherwise some of the stated outcomes of this work seem a little weak.
- Mechanics of forest disturbances on large herbivore response: In the introduction and elsewhere the authors should discuss (and provide citations) on the possible effects and mechanics of forest disturbances and the expected large herbivore response.
- Underlying effects: Would it be possible that there are other (ancillary reasons) that animals are evading undisturbed areas, for instance harvest might occur on the more accessible (and productive?) areas (evading rugged terrain) which might be the case for the herbivores as well. Would that generate the same results? Is there a way for the authors to address this?
- Separation of disturbance types: In the paper the authors mainly discuss “forest disturbances” as one type, however they analyzed different forest disturbance types that have different effects; discussion of the separate effects of these disturbances and why would improve the current manuscript, the understanding of the revealed patterns, and the applicability of the work.
- Intermediate disturbance hypothesis: This work lends itself well as supporting the intermediate disturbance hypothesis, therefore it would be interesting to add to this manuscript the percentages of occurrence of animals in disturbed and undisturbed areas. I assume that it is also important to have at least some undisturbed areas available. This is discussed in one of the last paragraphs in the Discussion, but seems a bit of an afterthought. As the paper is written now, it seems that more disturbances would always lead to a higher carrying capacity, but I assume that is only upto a certain level of disturbance. Inclusion of this concept might place the results of current MS in a larger context.

Minor comments:

L77: “Our findings suggest that higher levels of disturbance increases carrying capacity and concentrate large herbivores in disturbed areas.”-> Awkward How would “higher levels of disturbance concentrate large herbivores in disturbed areas”? I do not understand, rephrase or provide more context.

L78: “Both may amplify conflicts with forestry, but could also contribute to restoring large herbivores and their ecological

roles in forests.” This needs more context, I assume that you mean: “Given increased disturbance in the future these aspects may amplify .. etc”. But this is not what is written here.

L80: Consider adding “long term” in between “considering” and “disturbance”.

L103: “Our understanding of the impact of forest disturbance on large herbivores remains limited due to a lack of systematic assessments covering multiple species, wide environmental gradients, and long time periods.” I am not totally sure, but there is probably some research done before; this statement should have a bit more depth or citations that underscore this statement.

Fig 1: “relative changes in habitat suitability compared to the five year average prior to a disturbance event (in units of standard deviations).” This is where my statistical understanding is maybe a bit lacking (I am not an animal movement ecologist), but I do not understand the units “standard deviations”, normally, sd is indicating variability around a mean value, do a deviation from “normal”, which is indicated by anomalies. Maybe the sd term is a normal term that comes out of this modeling framework, please check.

L132: Using selection strength as an indicator of relative habitat suitability27 -> I am not sure if this is correct, and I am not a wildlife movement ecologist, therefore I recommend asking an expert here.

Figure 2: I assume that the disturbance agents are not equally represented in the dataset, for the last column in the figure provide the number of locations by disturbance agent. Also, it would be good to know the spatial resolution of the dNBR dataset here (add in the caption)?

Figure 3: The paper as written (might implicitly) assume that 2000 is the undisturbed situation, however the situation in 2000 is probably a culmination of disturbances that happened prior to 2000, how is this taken into account in Figure 3? Also, where is the “current” situation of the roe deer in the bottom left panel? Also I wonder what data is available is there geolocated animal data for the entire 2000-2023 period?

L243: “Our findings suggest that species’ habitat selection of disturbed forest areas is species-specific and primarily governed by differences in feeding ecology” Up to now there is no mention of “feeding ecology” in the MS, but in the following paragraphs you do a good job or relating this to individual animal feeding strategies. You might consider mentioning the mechanisms that disturbances cause that might alter forage (are there specific examples of this in the literature) earlier in the manuscript? Maybe some of this in the introduction?
L282 – 285: Refer to results here, what is the actual contribution of carnivore effects compared to other variables, can you quantify this? Same with human pressure?

Reviewer #2 (Remarks to the Author):

This study uses an impressively large dataset of GPS tracking data from >3,000 individuals of 4 large herbivore species (European bison, moose, red deer, roe deer) across Europe to assess the species responses to forest disturbances estimated by satellite remote sensing over recent decades. The authors used clustering and Random Forest models to estimate habitat selection and compare the strength of selection between samples of "disturbed and undisturbed forest areas", subsequently predicting changes in habitat suitability attributable to forest disturbance across Europe for the period 2000-2023. They report increased selection of disturbed areas by the large herbivores for 30+ years post-disturbance, and suggest these responses are driven by food selection and the characteristics of areas, indicative of trade-offs between resource selection and risk avoidance (e.g., predators, humans).

We commend the authors for assembling this unique dataset that represents considerable collective field effort, and for conducting a complex analysis across large spatial and temporal scales on a question of general relevance. Despite some concerns (detailed below), we appreciate the strengths of this manuscript and believe it will be of broad interest, particularly to researchers in the animal tracking community and those focused on large mammal ecology in Europe.

We had several comments, questions or concerns which we detail below in the hopes they can help strengthen the manuscript:

MAIN CONCERNS:

1. One shortcoming of the current manuscript (in our opinion) is that the specific research questions are not effectively set up in the Introduction, so the study comes across more as an opportunistic exploratory analysis motivated by (impressive) data availability than a study driven by specific ecological or management hypotheses. For instance:

a) the general focus on increasing forest disturbance has potential global relevance, but the manuscript seems to be largely missing specific context or explanation of forest disturbances in Europe, which we suspect vary within the region and differ from dynamics in other parts of the world (e.g. tropical regions, North America?). Are these primarily previously disturbed/managed (secondary/young) forests that are being newly disturbed, or rather old-growth forests? What is driving the increase in disturbance? How do the implied risks compare with other regions (e.g. lower predator abundance, extent/management of human hunting or conflict, severity of climate change impacts)? Without some additional context, the

framing seems a bit oversimplified and it is hard to assess the general relevance that is implied. (Further, we suggest clearer definitions of key terms such as "forested area" and "undisturbed" to help convey such context.)

b) Similarly, the focus on 4 large herbivore species that are well-known for using early successional vegetation for forage may lessen the impact of the results. Is it unexpected that these early successional browsers/grazers select younger and more heterogeneous forests? Perhaps the more interesting questions would dig deeper into more nuanced hypotheses such as those around forest patch size/configuration/age, thresholds of cumulative disturbance, or post-disturbance land-use management. In many other regions undergoing high levels of forest disturbances there are large herbivores that are likely more dependent on mature forests (e.g. woodland caribou, tapir, etc) and/or subject to very different risks (e.g., Ripple et al. 2015. Collapse of the world's largest herbivores. Science Advances 1:e1400103). Either a more focused framing of the study, or perhaps some broader/nuanced discussion, might help place it more clearly in a global context for international readers.

c) The Introduction mentions several variables of interest, including body size, foraging strategy, seasonal variation, disturbance characteristics, environmental gradients, but no hypotheses or predictions are presented to set up tests of these factors or demonstrate how this study builds on previous knowledge for these relatively well-studied species and regions. Rather, the interpretations appear more post-hoc (i.e., weaker inference).

2. The authors focus on a behavioural indicator (habitat selection) and make the assumption (mentioned on line 504) that their measure of habitat selection reflects fitness benefits for the focal species. Conclusions around increased carrying capacity (line 78) and "substantial positive and long-lating effects" (line 243) may require more critical evaluation, since the links between habitat use and fitness or demographic response are not always straightforward (as in concepts of attractive sinks or ecological traps, e.g. Robertson & Hutto RL 2006 Ecology 87:1075–1085). There are many studies emphasizing that the relationship between selection and fitness is complex and may only be proportional under a restrictive set of assumptions (e.g., Matthiopoulos et al., 2015, 2020). We further question whether it is reasonable to compare selection strengths between different years if they represent different decisions made by individuals at different times with different available habitats and (likely) different population densities? Density-dependence in habitat selection will be especially relevant at these large spatiotemporal scales.
It seems that large herbivore species such as the 4 examined here are generally well-studied and their population abundances are often monitored for hunting management. Can the authors further support their focus on habitat selection in relation to their conclusions around population-level consequences?

Matthiopoulos, J., Fieberg, J., & Aarts, G. (2020). Species-Habitat Associations: Spatial data, predictive models, and ecological insights. https://doi.org/10.24926/2020.081320
Matthiopoulos, J., Fieberg, J., Aarts, G., Beyer, H. L., Morales, J. M., & Haydon, D. T. (2015). Establishing the link between habitat selection and animal population dynamics. Ecological Monographs, 85(3), 413–436. https://doi.org/10.1890/14-2244.1

3. We are confused around the treatment of individual heterogeneity in habitat selection, which can be an important source of variation. It seems that individual-level information (except for sex) only enters the random forest models in the generation of background 0's within home ranges. The subsampling procedure (lines 428-430) would also lead to a data imbalance at the individual level which may be problematic if there is unmodeled individual heterogeneity.

4. We have some confusion/concern around the model predictions, specifically:

a) No assessment of model adequacy is presented for the final models (AUC is only presented in the supplement S2 in the context of tuning random forests). And comparing figures 4 and 5 suggests some significant extrapolation of predictions beyond the ranges of animal tracking for most species (and perhaps some uneven sampling across forest cover and disturbance, although hard to discern). This should be more transparently acknowledged/discussed.

b) Can the authors further justify the rationale/validity of holding all environmental variables at 2023 levels and predicting suitability based only on forest disturbance for 2000-2023?

c) The habitat selection models were built at the 3rd order of habitat selection (i.e., within home range) but the predictions seem to more at the 2nd-order of selection (i.e., home range location, 100 km-wide hexagons), although this is not clearly stated. Can the authors clarify/justify this apparent discrepancy in scale?
Furthermore, we wonder about the use of predictors at the home range scale (Extended Table 1), which seems at odds with subsequent interpretation of results based on foraging decisions within home ranges. If the buffer radius was almost as large as the home range, the sampled points will have (nearly) identical values and thus little variation for assessing within-home-range foraging behaviour (not to mention the high spatial autocorrelation among points within home ranges). In general, we felt the moving window averaging method needed to be more clearly explained.

ADDITIONAL COMMENTS:

- line 76 "Across Europe ..." sentence seems out of place, not well connected to flow of abstract

- the unique spatial and temporal scale of the data could be more strongly emphasized in the abstract, as on line 110 (in place of generic statements such as navigating trade-offs between resources and risk)

- acknowledging the "Methods last" format of the journal, it would still be helpful to make brief mention of the general approach to modelling earlier in the paper, as many statements are hard to interpret without some methodological context

- Fig 1: please explain/justify why 40th to 60th percentile range used to show responses

- Fig 2: please explain the bootstrapping details for obtaining 95% CI

- paragraph starting line 191 - why are there no references to figures/tables to support these results?

- Fig 3: why is current range not shown for roe deer?

- Fig 4: should the caption say 2000-2023?

- lines 263-265: This could explain why the peaks of bison and moose selection differ, but then why is bison selection significantly elevated even after 37 years, but moose selection is in decline? Further, woody species (like Rubus) are not typically found one year post disturbance; herbaceous species (graminoids and forbs) usually establish first. We are concerned that inferences are over-stepping the strength of evidence.

- line 335 - missing period between sentences

- line 347 - delete "be"

- the Methods imply both 5 available points to 1 used, and balance between presence/background, please clarify

- line 402: justification for buffer size? is 7 hours long enough to mitigate autocorrelation?

- line 426: why were background points restricted to forest areas?

- lines 449-450: mismatch in temporal scale between annual disturbance data and monthly resolution of analysis. Could some locations in undisturbed forests be classified as recent disturbance if the disturbance occurred at a later month? Some months misclassified?

- lines 456-459: justification for choices of -100 and 100? Results could be sensitive to this especially since a lot of "undisturbed" forest is probably still disturbed from the perspective of focal species.

- lines 476-478: scale of animal home range? The method is not clear. Did the authors create a buffer around used and available points with a radius equal to home range?

- line 480: typo - "large habitat" (missing "ungulate")

- line 513: would be helpful to briefly explain PDPs

- lines 548-549: Predictions were made for both sexes, but which results are shown in e.g. Figure 4?

- line 565: this sentence is confusing as written: "in undisturbed forest areas, the only variable that was allowed to vary across years was the area of recently disturbed forest."

- lines 566-569: undisturbed as of 2023 or undisturbed throughout the entire period of disturbance data? If the former, why weren't other disturbance metrics varied? If the latter, wouldn't the area of recently disturbed forest always be zero? Please clarify the specific meaning of terms like "forested" or "undisturbed" (time period, spatial scale, etc.).

- Supplement S1: clustering method not clear, including use of PCA vs. individual environmental variables. Please explain "97% of the observed variance" in what? the environmental attributes of the tracking locations? What is the overall purpose of clustering?

- Why are the maps of predators not shown anywhere? We are concerned that these may overestimate predation risk (given low abundance / uncertain occupancy?). The emphasis on the "weak negative effect ... in some cases" (line 184) may be stretching the strength of evidence presented?

Reviewer #3 (Remarks to the Author):

I co-reviewed the manuscript in collaboration with one of the referees who provided a report.

Reviewer #4 (Remarks to the Author):

I co-reviewed the manuscript in collaboration with one of the referees who provided a report.

Version 1:

Decision Letter:

12th March 2026

Dear Julian,

Thank you for submitting your revised manuscript "Increasing forest disturbance enhances habitat suitability for Europe’s large herbivores" (NATECOLEVOL-25103544A). Based on the reviewers' comments (see the reports below), we will be happy in principle to publish it in Nature Ecology & Evolution, pending minor revisions and compliance with our editorial and formatting guidelines.

We are now performing detailed checks on your paper and will send you a checklist detailing our editorial and formatting requirements in about a week. Please do not upload the final materials and make any revisions until you receive this additional information from us.

Please do not hesitate to contact me if you have any questions.

[redacted]

**

Reviewer #1 (Remarks to the Author):

The authors have adequately addressed my concerns. I commend the authors for such a thorough and thoughtful response.

Reviewer #2 (Remarks to the Author):

I read carefully through the authors' responses to all reviewer comments, and through the revised manuscript. In my opinion the authors have done an excellent job of responding to comments and revising the manuscript, which is now clearer and stronger overall. I am satisfied with the responses/revisions and think the manuscript will make an excellent contribution to the literature.

I only have the following very minor comments:

- line 80: "increasing disturbance rates improved habitat suitability" - I don't think it is the increasing rates per se, but rather the amount of disturbance that is driving the change in suitability?

- paragraph starting line 109: suggest also briefly highlighting the importance of large herbivores in addition to their ecological role (e.g. cultural & economic values). And perhaps same in concluding sentence (L428-430)

- line 228: not clear here what is meant by "potential range extents" - does this mean within historic range?

- line 297: add "the" or "a" to "...foraging preferences are [the] main factor driving..."

- line 362: I recommend changing "show" to "suggest" (or similar) since these are model projections, derived while holding other variables at 2023 levels, rather than more direct observations of actual changes in habitat suitability (which is implied by the current wording). Similarly, add "Projected improvements" to start next sentence on line 365.

Open Access This Peer Review File is licensed under a Creative Commons Attribution 4.0 International License, which permits use, sharing, adaptation, distribution and reproduction in any medium or format, as long as you give appropriate credit to the original author(s) and the source, provide a link to the Creative Commons license, and indicate if changes were made.
In cases where reviewers are anonymous, credit should be given to 'Anonymous Referee' and the source.
The images or other third party material in this Peer Review File are included in the article's Creative Commons license, unless indicated otherwise in a credit line to the material. If material is not included in the article's Creative Commons license and your intended use is not permitted by statutory regulation or exceeds the permitted use, you will need to obtain permission directly from the copyright holder.
To view a copy of this license, visit https://creativecommons.org/licenses/by/4.0/

Reviewer #1

**#1**	The authors have adequately addressed my concerns. I commend the authors for such a thorough and thoughtful response.	Thank you very much for your constructive review, we highly appreciate it.

Reviewer #2

**#1**	I read carefully through the authors' responses to all reviewer comments, and through the revised manuscript. In my opinion the authors have done an excellent job of responding to comments and revising the manuscript, which is now clearer and stronger overall. I am satisfied with the responses/revisions and think the manuscript will make an excellent contribution to the literature.<br><br>I only have the following very minor comments:	Thank you very much for your efforts in reviewing our paper, the constructive and thoughtful reviews were very helpful for improving our manuscript.<br><br>We have incorporated your minor suggestions and provide responses to your individual comments below.
**#2**	- line 80: "increasing disturbance rates improved habitat suitability" - I don't think it is the increasing rates per se, but rather the amount of disturbance that is driving the change in suitability?	Good point. We agree that this wording may be a bit "indirect". Thus, we changed it to: *"[...] that the recent increase in forest disturbances improved habitat suitability for our study species across Europe [...]".*
**#3**	- paragraph starting line 109: suggest also briefly highlighting the importance of large herbivores in addition to their ecological role (e.g. cultural & economic values). And perhaps same in concluding sentence (L428-430)	Thank you for the comment. We have added a statement according to your suggestion to our Introduction (Line 100-101):<br>*"Large herbivores play key roles in shaping forest ecosystems and are also of considerable cultural and economic importance[11–13]. "*
**#4**	- line 228: not clear here what is meant by "potential range extents" - does this mean within historic range?	Great comment. We agree that the meaning of potential range extents could be explained better. In the context of our work, potential range extents do not refer to historical ranges, but counterfactual natural ranges, i.e., estimates of where species would occur without human influence, which can be used as a baseline for restoration. To clarify this, we added an

		explanation at the first mention of this term in the Results section:<br>*"[...] we created monthly predictions of habitat suitability between 2000 and 2023 across representative samples of disturbed and undisturbed forest areas within the species' current and potential range extents (i.e., estimates of where species would occur without human influence) in Europe."*<br><br>In addition, we added a sentence to our Methods section (Line 578ff.):<br>*"Potential range extents correspond to counterfactual natural ranges, i.e., areas in which species would occur without human influence."*
**#5**	- line 297: add "the" or "a" to "...foraging preferences are [the] main factor driving..."	Done.
**#6**	- line 362: I recommend changing "show" to "suggest" (or similar) since these are model projections, derived while holding other variables at 2023 levels, rather than more direct observations of actual changes in habitat suitability (which is implied by the current wording). Similarly, add "Projected improvements" to start next sentence on line 365.	Thank you for the helpful suggestions. We chose "indicate" instead of "show", and rephrased to "Projected improvements" as per your suggestion.

Responses to the reviewers

Comments by reviewer #1

#	Comment	Response
**1**	In the manuscript the authors analyze forest disturbance effects on an extensive animal tracking dataset of four large herbivore species across Europe. They provide evidence that forest disturbance enhances habitat suitability for Europe's large herbivores, an interesting finding. Aside from the modeling framework (on which I cannot comment, since I am not an animal movement ecologist) and thus would refer to an expert on the applicability of their methodology. I found several relatively major issues that need to be addressed before the paper can be published in Nature Ecology and Evolution. In summary, I think this is a worthwhile (enormous?!) effort with interesting findings that would be of interest to Nature Ecology and Evolution provided that the authors address the comments below and pending an expert review on the statistical methods performed.	Thank you very much for your thorough and constructive review of our study. We truly appreciate the time and effort spent on evaluating our work carefully.<br><br>We have revised our manuscript based on your comments and provide point-by-point responses below. Your comments were very valuable, particularly in the context of further strengthening the conceptual framing of our study. Where necessary, we have added additional analyses to make our findings more robust against sources of bias and provide additional validation of our results.<br><br>Relevant changes made to the manuscript are quoted in our responses, with line numbers in square brackets referring to the revised version of the manuscript.
**2**	Increasing forest disturbances: A lot of the motivation of this paper relies on the premise that forest disturbances are increasing, however there is not a lot of recent citations that cover the current study area (Europe) that show this trend and why this is happening. Proof of this and corresponding citations might be available, and it would be good for the authors to include this or build out this expected trend with proof, otherwise some of the stated outcomes of this work seem a little weak.	Thank you for the comment. We have edited the relevant section in the introduction to provide clearer examples. In addition, we provide additional references to studies documenting the recent increase in forest disturbances in Europe (and elsewhere).<br><br>*Over the last decades, intensifying forest use and higher rates of natural disturbances, attributed primarily to climatic extremes[4,5], have caused a drastic increase in the extent of forest disturbances in some regions[6,7]. For example, canopy mortality doubled in Europe's temperate forests between 1984-2016[8], and the forest area disturbed by fires globally was 2.2 times higher in 2023-2024 than the long-term average since 2001[9].* [L91-95]<br><br>Below is an overview of the newly added references:

		McDowell NG, Allen CD, Anderson-Teixeira K, et al (2020) Pervasive shifts in forest dynamics in a changing world. Science 368:eaaz9463. https://doi.org/10.1126/science.aaz9463<br><br>Patacca M, Lindner M, Lucas-Borja ME, et al (2023) Significant increase in natural disturbance impacts on European forests since 1950. Glob Change Biol 29:1359–1376. https://doi.org/10.1111/gcb.16531<br><br>Potapov P, Tyukavina A, Turubanova S, et al (2025) Unprecedentedly high global forest disturbance due to fire in 2023 and 2024. Proc Natl Acad Sci 122:e2505418122. https://doi.org/10.1073/pnas.2505418122<br><br>Senf C, Buras A, Zang CS, et al (2020) Excess forest mortality is consistently linked to drought across Europe. Nat Commun 11:6200. https://doi.org/10.1038/s41467-020-19924-1<br><br>Senf C, Pflugmacher D, Zhiqiang Y, et al (2018) Canopy mortality has doubled in Europe's temperate forests over the last three decades. Nat Commun 9:4978. https://doi.org/10.1038/s41467-018-07539-6<br><br>Senf C, Seidl R (2021) Storm and fire disturbances in Europe: Distribution and trends. Glob Change Biol 27:3605–3619. https://doi.org/10.1111/gcb.15679
**3**	Mechanics of forest disturbances on large herbivore response: In the introduction and elsewhere the authors should discuss (and provide citations) on the possible effects and mechanics of forest disturbances and the expected large herbivore response.	We agree that providing context to the underlying mechanisms is important and this was perhaps a bit too short in our prior manuscript version.<br><br>We now discuss the mechanisms of disturbance impacts on large herbivore habitat suitability in more detail in the second paragraph of the introduction. Specifically, we have revised this section to provide a more detailed explanation of potential mechanisms and derive expectations based on available literature.<br><br>*Forest disturbances may strongly impact large herbivore habitat suitability, since they directly affect the fundamental trade-off faced by many large herbivores between accessing forage and cover* [11]*. By killing canopy trees, forest disturbances increase light availability in the understory, encouraging the growth of early successional plants, and resulting in higher forage availability for many species*[12,13]*. Due to the seasonal variation in*

		foraging preferences[14], the suitability of disturbed areas for large herbivores may vary considerably throughout the year[15]. Disturbances can intermittently reduce the availability of vegetation cover, and create open habitats in which animals are exposed to predation, human activities, thermal extremes, and snow[16–18]. Yet, depending on disturbance characteristics (e.g., disturbance patch size and remaining deadwood) and vegetation recovery, disturbed areas can provide habitats offering both forage and cover[19]. Thus, large herbivore responses to forest disturbance can be expected to strongly depend on species' feeding ecology and disturbance characteristics, and further show considerable seasonal variation.<br>[L97-108]
**4**	Underlying effects: Would it be possible that there are other (ancillary reasons) that animals are evading undisturbed areas, for instance harvest might occur on the more accessible (and productive?) areas (evading rugged terrain) which might be the case for the herbivores as well. Would that generate the same results? Is there a way for the authors to address this?	Thank you for the thoughtful comment. We agree that being aware of potential confounding factors is critical.<br><br>We are controlling for the effects of other factors influencing habitat suitability (e.g., terrain ruggedness, climate, and human pressure) specifically through including these variables as control factors in our models (i.e., predictor variables in our habitat selection models).<br><br>In addition, we would like to note that due to the time series-nature of our analysis, our models allow us to directly assess the change of habitat suitability caused by disturbances (i.e., how does habitat selection / suitability change in response to forest disturbance happening at a given site). In other words, the spatial bias problems you describe that would be a concern for pure space-for-time comparisons are less of a concern in our case. Thus, we believe our study design should be robust against confounding effects related to shared preferences for accessible or productive terrain.
**5**	Separation of disturbance types: In the paper the authors mainly discuss "forest disturbances" as one type, however they analyzed different forest disturbance types that have different effects; discussion of the separate effects of these disturbances and why would improve the current manuscript, the	Based on our available datasets, we did test for varying effects of disturbance agents, but mostly did not find clear differences between disturbance types. Nevertheless, we fully agree that a more nuanced discussion of

	understanding of the revealed patterns, and the applicability of the work.	potential variation of impacts across different disturbance types is useful.<br><br>Thus, we have expanded and revised our results section to talk more about disturbance agents, and we added a new Supplementary Figure (Figure S1) to add more information on the relative frequency of disturbance agents in our dataset.<br><br>*The occurrence of disturbance agents varied strongly in our dataset, with harvests being the most common agent and fire disturbances being particularly rare (37-79% vs. 0.1%-1.4% of all disturbances within the tracking data extents per species; see Supplementary Figure S1)*<br>[L188-191]<br><br>In addition, we edited the Discussion section to discuss potential differences between disturbance types more thoroughly. In this context, we also highlight that more fine-scale analyses integrating information on post-disturbance management (e.g., salvage logging), or studies focusing on disturbance in unmanaged forest ecosystems would be required to gain deeper insights into the potentially varying effects of different disturbance agents.<br><br>*Disturbances in our dataset were dominated by harvests and bark beetle/wind disturbances, accounting for more than 90% of disturbance events across species. We observed relatively little variation in habitat selection across disturbance agents, such as the relative avoidance of wildfire-related disturbances by red deer, matching previous findings[52], and bark beetle/wind disturbances by European bison. However, we caution that our dataset was not well suited for characterizing varying impacts of disturbance agents due to frequent post-disturbance management in European forests[53]. Since standing and lying deadwood resulting from natural disturbances is commonly removed, agent-specific impacts may be masked by management and thus underestimated by our analysis. Post-disturbance management interventions such as salvage logging or tree planting can influence forage availability, vegetation cover, and movement barriers*

		created by deadwood[54]. *To better understand the effects of post-disturbance management and disentangle impacts of disturbance agents and associated characteristics, studies focusing on unmanaged disturbances (e.g., in protected areas) or integrating fine-scale datasets on post-disturbance management measures are needed*[15].<br>[L317-329]
**6**	Intermediate disturbance hypothesis: This work lends itself well as supporting the intermediate disturbance hypothesis, therefore it would be interesting to add to this manuscript the percentages of occurrence of animals in disturbed and undisturbed areas. I assume that it is also important to have at least some undisturbed areas available. This is discussed in one of the last paragraphs in the Discussion, but seems a bit of an afterthought. As the paper is written now, it seems that more disturbances would always lead to a higher carrying capacity, but I assume that is only upto a certain level of disturbance. Inclusion of this concept might place the results of current MS in a larger context.	Thank you for this thoughtful comment. We agree that this issue requires a more thorough discussion in our paper.<br><br>We analyzed effects related to disturbance levels in the landscape by including a predictor quantifying the amount of recently disturbed areas at a scale of species' home range sizes. Thus, our predictions of habitat suitability trends account for potential negative effects of high disturbance levels. To make the implications of our findings clearer and better highlight potential limits of disturbance-related benefits to large herbivores, we have made minor adjustments to the relevant part of the Discussion section.<br><br>*While our results indicate that current levels of forest disturbance in Europe are beneficial for all four study species, it is important to consider potential negative effects if disturbance rates continue to increase due to climate change (e.g., due to increased insect outbreaks, fires or windthrows) or intensified forest use. All species responded negatively to increasing disturbance patch size and high levels of disturbed areas inside their home ranges. These patterns suggest that further increases in disturbance levels, particularly in the form of large disturbance patches, could eventually result in net-negative impacts on habitat suitability for large herbivores.*<br>[L392-398]<br><br>In addition, we revised our manuscript in multiple places to discuss this issue in more detail. For instance, we added a sentence to the introduction aiming to make readers aware of potential limits associated with disturbance benefits for large herbivores.<br><br>*Increasing disturbance rates may benefit large herbivores that capitalize on early-*

		successional vegetation[15], but extensive forest disturbance has also been identified as a threat to some large herbivore populations[24,25]<br>[L120-122]<br><br>Further, we added a sentence to our Discussion section highlighting that other large herbivores not covered by our assessment may be negatively impacted by increasing disturbance levels.<br><br>*Our analysis covered the largest native strict herbivores with potential natural ranges spanning much of Europe, reflecting a large gradient in dietary niches[14]. Despite considerable intraspecific variation and declines in habitat suitability after disturbance in some cases, we found overall positive responses by all species, in line with previous local assessments [12,44]. Yet, we caution that some large herbivores with a strong dependence on mature forests may respond negatively to forest disturbances (e.g., reindeer Rangifer tarandus, depending on undisturbed forest for lichens as key food items during winter[45])*<br>[L299-305]<br><br>While we agree that the intermediate disturbance hypothesis is an interesting concept in this regard, we refrained from explicitly referencing this theory in our interpretations to avoid confusing readers, since in its original form, the theory focuses on explaining the relationship between disturbance and species richness (i.e., a community-level concept).
**7**	L77: "Our findings suggest that higher levels of disturbance increases carrying capacity and concentrate large herbivores in disturbed areas."-> Awkward How would "higher levels of disturbance concentrate large herbivores in disturbed areas"? I do not understand, rephrase or provide more context.	We rephrased the sentence to improve its clarity. We now highlight the strongly attracting effect of disturbances on large herbivores shown by our habitat selection models, causing a spatial concentration of large herbivores within disturbed areas.<br><br>*Our findings indicate that forest disturbances can have strong attraction effects for large herbivores and that increasing disturbance rates improved habitat suitability for our study species across Europe.*<br>[L78-80]

**8**	L78: "Both may amplify conflicts with forestry, but could also contribute to restoring large herbivores and their ecological roles in forests." This needs more context, I assume that you mean: "Given increased disturbance in the future these aspects may amplify .. etc". But this is not what is written here.	Very good point. We agree that more context is needed here. We rephrased the sentence according to your suggestion.<br>*Given expected future increases in disturbance, resulting habitat improvements could amplify conflicts with forestry, but also contribute to restoring large herbivores and their ecological functions in forests.*<br>[L82-84]
**9**	L80: Consider adding "long term" in between "considering" and "disturbance".	Thank you, we followed your suggestion and added "long-term" to this sentence.
**10**	L103: "Our understanding of the impact of forest disturbance on large herbivores remains limited due to a lack of systematic assessments covering multiple species, wide environmental gradients, and long time periods." ⊒ I am not totally sure, but there is probably some research done before; this statement should have a bit more depth or citations that underscore this statement.	We fully agree that this statement should be more nuanced. We have revised the sentence to acknowledge prior research and added supporting citations to underscore this gap.<br>*While several studies have examined the effects of forest disturbance on large herbivore species at local or regional scales[15,19,21], our understanding remains limited due to a lack of systematic assessments covering broad spatial extents, multiple species, and long time periods.*<br>[L116-118]
**11**	Fig 1: "relative changes in habitat suitability compared to the five year average prior to a disturbance event (in units of standard deviations)." □ This is where my statistical understanding is maybe a bit lacking (I am not an animal movement ecologist), but I do not understand the units "standard deviations", normally, sd is indicating variability around a mean value, do a deviation from "normal", which is indicated by anomalies. Maybe the sd term is a normal term that comes out of this modeling framework, please check.	We agree that this wording was unclear and could be misleading.<br>In our analysis, "standard deviations" do not refer to statistical variability or anomalies around a normal distribution. Instead, habitat suitability values were standardized to facilitate comparison across species and time. Thus, the units in Fig. 1 and elsewhere represent changes in standardized habitat suitability relative to a reference distribution.<br>Specifically, habitat suitability predictions were standardized using the distribution of predicted suitability values across forest areas within species' current range extents (in the year 2000). Disturbance responses therefore quantify how strongly suitability in disturbed areas deviates from this reference, expressed in units of standard deviations of suitability. This enables robust cross-species comparisons, since the absolute ranges and variance of habitat suitability values may vary from one species to the next due to their dependence on background sampling.

		We have revised the figure caption and Methods text to clarify this explicitly.<br><br>Methods section:<br><br>*To facilitate comparisons across species, we standardized habitat suitability predictions across species, since the absolute values model predictions typically depend on the definition of habitat availability[37]. Specifically, we used the mean and standard deviation of suitability values across forest areas within species' current range extents in the year 2000. Changes are therefore expressed in units of standard deviations relative to forest habitat suitability in the year 2000. We applied the same standardization for all model predictions (i.e., for calculating partial dependence plots, disturbance responses, and suitability changes across Europe).*<br>[L578-L584]<br><br>Figure caption:<br><br>*To facilitate comparisons across species, disturbance responses are shown as relative changes in standardized habitat suitability compared to the five-year average prior to a disturbance event, where standardization is based on the distribution of forest habitat suitability in the year 2000.*<br>[L160-L163]
**12**	L132: Using selection strength as an indicator of relative habitat suitability27 -> I am not sure if this is correct, and I am not a wildlife movement ecologist, therefore I recommend asking an expert here.	We agree that this interpretation requires careful justification and clear acknowledgment of its assumptions.<br><br>Interpreting selection strength as relative habitat suitability is common in habitat-selection theory. We describe the assumptions underlying our interpretation of modeling results in the Methods section.<br><br>We have revised the Methods section, in which we now more clearly define our assumptions, cite established literature on this issue, and clarify limitations of our models (i.e., not accounting for density-dependent selection effects).<br><br>*Given the use-availability design underlying our models, the predictions by our models indicate relative intensities of habitat selection[108]. Further, assuming habitat*

<table>
<tr><td></td><td></td><td>selection reflects informed decisions of animals to maximize their fitness, model predictions can be interpreted as expected fitness payoffs[37]. We thus follow the common interpretation of model predictions as indicators of relative habitat suitability, but acknowledge that our models do not account for changes in habitat selection due to varying population densities[37,109].
[L573-L578]</td></tr>
<tr><td>13</td><td>Figure 2: I assume that the disturbance agents are not equally represented in the dataset, for the last column in the figure provide the number of locations by disturbance agent. Also, it would be good to know the spatial resolution of the dNBR dataset here (add in the caption)?</td><td>Thank you for the helpful comment and for raising this issue.

We have added sentences to the Results and Discussion sections to be more explicit about the relative frequency of disturbance agents. In addition, we included a Supplementary Figure (Figure S1) giving a detailed overview of the proportion of disturbance agents in our dataset.

Results section:

The occurrence of disturbance agents varied strongly in our dataset, with harvests being the most common agent and fire disturbances being particularly rare (37-79% vs. 0.1%-1.4% of all disturbances within the tracking data extents per species; see Supplementary Figure S1).
[L188-191]

Discussion section:

Disturbances in our dataset were dominated by harvests and bark beetle/wind disturbances, accounting for more than 90% of disturbance events across species.
[L317-318]

We also revised the figure caption to add information on the spatial scale of the disturbance severity variable (ΔNBR), which was calculated by averaging 30-m pixels at the scale of disturbance patches.

Disturbance severity reflects the difference in the Normalized Burn Ratio (NBR) averaged at the scale of disturbance patches.
[L201-L203]</td></tr>
</table>

**14**	Figure 3: The paper as written (might implicitly) assume that 2000 is the undisturbed situation, however the situation in 2000 is probably a culmination of disturbances that happened prior to 2000, how is this taken into account in Figure 3?	Thank you for raising this important issue. We agree that this was indeed a potential source of bias in our original analysis.<br><br>We would like to point out that our disturbance maps cover the period of 1986-2023, so the year 2000 was not considered to be an undisturbed state. We limited the prediction period to 2000-2023 to match the availability of tracking data (1997-2023 across species) and other environmental predictors used in our models. Our disturbance histories, however, go further back, to the mid 1980s.<br><br>Nevertheless, we shared your concern about potential bias stemming from including the variable "years since disturbance" in our models, since this could introduce a truncation bias due to lack of information on disturbances happening before 1986, given the long-lasting effects of disturbances.<br><br>Thus, we re-ran our models excluding the 'year since disturbance' variable, and adding four satellite-based metrics as predictors. This allowed us to build models which, instead of relying on the year since disturbance variable, primarily use the time series of spectral metrics to detect forest disturbances and their effects on vegetation dynamics such as disturbance, recovery, and maturation (see Oeser et al. 2021 for an application of this approach).<br><br>Therefore, these models should be robust to truncation bias associated with a lack of explicit spatial information on the occurrence of disturbance happening before our prediction period. In our revised version of the manuscript, we now report habitat suitability trends as predicted by these models. The resulting trends are consistent with our previous results, but show slightly lower suitability increases for all species. We have revised the Methods and Results sections to reflect these changes.<br><br>Methods section:<br><br>*To assess changes in habitat suitability caused by recent disturbance dynamics in Europe, we carried out a second model prediction across the current and potential*

		range extents of species. For this prediction, we used separately trained models not containing information on the years since disturbance as a predictor variable. We excluded this variable to avoid a truncation bias due to a lack of information on disturbances happening before 1986. Instead, we added four additional predictors based on spectral-temporal metrics derived from Tasseled Cap transformations of Landsat reflectance (median wetness and brightness, and inter-decile ranges of wetness and brightness). Thus, these models primarily leverage satellite-based metrics to characterize habitat suitability dynamics associated with forest disturbance, recovery, and maturation [15]*. We applied the same tuning and cross-validation approach described above, which again indicated excellent model transferability across environmental clusters (mean Continuous Boyce Index from cross-validation: 0.98, 0.95, 0.94, and 0.88, for European bison, moose, red deer, and roe deer, respectively). Disturbance responses obtained from models built without the "years since disturbance" variable were similar to those obtained from our original models (Supplementary Figure S8).*<br>[L640-653]<br><br>Results section:<br><br>*We carried out a second, counterfactual prediction to assess how recent forest disturbance dynamics have contributed to habitat suitability dynamics for each study species. Specifically, we projected models across the species' current and potential range extents for the period 2000-2023 and kept all variables unrelated to forest disturbance dynamics (e.g., climate, land cover, human pressure, carnivore presence) at 2023 conditions. We used this approach to isolate and quantify the contribution of forest disturbance on habitat suitability dynamics. Models used for these projections were built without information on time since disturbance in order to avoid truncation bias due to a lack of information on forest disturbances prior to 1986.*

		According to model projections, forest disturbances improved habitat suitability for all four large herbivore species between 2000-2023 across their current and potential ranges (Fig. 3). Disturbance-induced improvements in habitat suitability across the species' current ranges were highest for moose (0.75 standard deviations within disturbed areas and 0.22 standard deviations across all forest areas), followed by red deer (0.88 and 0.15), European bison (0.49 and 0.06), and roe deer (0.24 and 0.04). [L226-238]<br><br>**<u>References</u>**<br>Oeser J, Heurich M, Senf C, et al (2021) Satellite-based habitat monitoring reveals long-term dynamics of deer habitat in response to forest disturbances. Ecol Appl 31:e2269. https://doi.org/10.1002/eap.2269
**15**	Also, where is the "current" situation of the roe deer in the bottom left panel?	Thank you for highlighting this. We added a sentence to the Figure caption explaining why only one trend line is visible for roe deer.<br><br>*For roe deer, lines and points are strongly overlapping due to the current and potential range extents being almost identical.* [L251-252]
**16**	Also I wonder what data is available is there geolocated animal data for the entire 2000-2023 period?	Yes. There was GPS tracking data available for the entire 2000-2023 period, although for some species, individual years might not be covered. Please see Extended Data Fig. 2 displaying our data availability across time, which we revised to improve clarity.
**17**	L243: "Our findings suggest that species' habitat selection of disturbed forest areas is species-specific and primarily governed by differences in feeding ecology" ⊇ Up to nowthere is no mention of "feeding ecology" in the MS, but in the following paragraphs you do a good job or relating this to individual animal feeding strategies. You might consider mentioning the mechanisms that disturbances cause that might alter forage (are there specific examples of this in the literature) earlier in the manuscript? Maybe some of this in the introduction?	We agree that the potential importance of feeding ecologies for explaining differences across species could have been highlighted better in our Introduction. We revised the relevant part of the Introduction to better highlight this point and relate it to underlying mechanisms driving forage availability.<br><br>*By killing canopy trees, forest disturbances increase light availability in the understory, encouraging the growth of early successional plants, and resulting in higher forage availability for many species*[12,13]*. Due to the seasonal variation in foraging preferences*[14]*, the suitability of disturbed areas for large*

		herbivores may vary considerably throughout the year[15]. Disturbances can intermittently reduce the availability of vegetation cover, and create open habitats in which animals are exposed to predation, human activities, thermal extremes, and snow[16–18]. Yet, depending on disturbance characteristics (e.g., disturbance patch size and remaining deadwood) and vegetation recovery, disturbed areas can provide habitats offering both forage and cover[19]. Thus, large herbivore responses to forest disturbance can be expected to strongly depend on species' feeding ecology and disturbance characteristics, and further show considerable seasonal variation. [L99-108]
**18**	L282 – 285: Refer to results here, what is the actual contribution of carnivore effects compared to other variables, can you quantify this? Same with human pressure?	We agree that this needed more explanation and revised this part of the Discussion section to provide numbers which allow comparing the relative strength of effects of carnivore variables vs. other environmental variables in our models.<br><br>*We found only weak associations between broad-scale distribution maps for large carnivores and habitat selection by our study species (i.e., relative effects on habitat suitability of up to 0.3 vs. up to 2.0 standard deviations for other variables)* [L343-345]<br><br>More generally, we have altered our approach for evaluating partial dependence plots (PDPs) from one-dimensional PDPs per variable to two-dimensional PDPs across bins of the year since disturbance variable and all other predictors. This allows us to provide a more complete picture of the relative influence of environmental influence at different stages of forest recovery after disturbance. Based on these two-dimensional PDPs, we quantify the impact of variables as their relative effect on predicted suitability within each bin of the "years since disturbance" variable. Since a detailed discussion of all environmental variable effects would have gone beyond the scope of the main text, we have added a Supplementary Note (S2), in which we provide a detailed description and interpretation of

		environmental variable effects (e.g., human pressure, climate, etc.).<br><br>Considering the effects of carnivore variables, we have revised our Discussion section towards a more cautious interpretation (see also our response to comment #32 by reviewer #2).

Comments by reviewer #2

#	Comment	Response
**1**	This study uses an impressively large dataset of GPS tracking data from >3,000 individuals of 4 large herbivore species (European bison, moose, red deer, roe deer) across Europe to assess the species responses to forest disturbances estimated by satellite remote sensing over recent decades. The authors used clustering and Random Forest models to estimate habitat selection and compare the strength of selection between samples of "disturbed and undisturbed forest areas", subsequently predicting changes in habitat suitability attributable to forest disturbance across Europe for the period 2000-2023. They report increased selection of disturbed areas by the large herbivores for 30+ years post-disturbance, and suggest these responses are driven by food selection and the characteristics of areas, indicative of trade-offs between resource selection and risk avoidance (e.g., predators, humans).<br><br>We commend the authors for assembling this unique dataset that represents considerable collective field effort, and for conducting a complex analysis across large spatial and temporal scales on a question of general relevance. Despite some concerns (detailed below), we appreciate the strengths of this manuscript and believe it will be of broad interest, particularly to researchers in the animal tracking community and those focused on large mammal ecology in Europe.	Thank you very much for this detailed, positive, and constructive review of our study. We greatly appreciate the recognition of the substantial collaborative effort associated with our study.<br><br>We have carefully considered the concerns raised below and revised the manuscript accordingly to improve clarity, conceptual framing, and methodological transparency. Where necessary, we have added analyses to make our findings more robust against sources of bias and provide additional validation of our results. We believe these revisions have significantly strengthened the manuscript.<br><br>Relevant changes made to the manuscript are quoted in our responses, with line numbers in square brackets referring to the revised version of the manuscript.
**2**	One shortcoming of the current manuscript (in our opinion) is that the specific research	We fully agree that the specific study context requires a more detailed introduction. We

	questions are not effectively set up in the Introduction, so the study comes across more as an opportunistic exploratory analysis motivated by (impressive) data availability than a study driven by specific ecological or management hypotheses. For instance:<br><br>a) the general focus on increasing forest disturbance has potential global relevance, but the manuscript seems to be largely missing specific context or explanation of forest disturbances in Europe, which we suspect vary within the region and differ from dynamics in other parts of the world (e.g. tropical regions, North America?). Are these primarily previously disturbed/managed (secondary/young) forests that are being newly disturbed, or rather old-growth forests? What is driving the increase in disturbance? How do the implied risks compare with other regions (e.g. lower predator abundance, extent/management of human hunting or conflict, severity of climate change impacts)? Without some additional context, the framing seems a bit oversimplified and it is hard to assess the general relevance that is implied. (Further, we suggest clearer definitions of key terms such as "forested area" and "undisturbed" to help convey such context.)	have revised our Introduction section to provide more information on European forests, their management, as well as disturbance dynamics and their underlying drivers.<br><br>*We focused on four native large herbivore species with wide potential natural ranges (European bison Bison bonasus, moose Alces alces, red deer Cervus elaphus, and roe deer Capreolus capreolus), reflecting gradients in terms of foraging strategies[14]. European forests are heavily managed with few intact old-growth forests[29] and a majority of disturbances are caused by human forest use[30]. Forest disturbances in Europe have increased markedly over the last decades[7,8,27], with increasing natural disturbances and intensifying forest use interacting as drivers[30].* [L127-133]<br><br>Regarding clearer definitions of “disturbed areas” and “undisturbed areas”, we revised our Results section and have added definitions of relevant terms to the short method overview we provide at the beginning of this section.<br><br>*These forest disturbance maps capture stand-replacing forest disturbance events at 30m spatial resolution annually between 1986-2023. In the context of our analysis, we considered all areas with a detected disturbance event as disturbed forest, while all other areas were considered undisturbed forest.*<br>[L149-152]
**3**	Similarly, the focus on 4 large herbivore species that are well-known for using early successional vegetation for forage may lessen the impact of the results. Is it unexpected that these early successional browsers/grazers select younger and more heterogeneous forests? Perhaps the more interesting questions would dig deeper into more nuanced hypotheses such as those around forest patch size/configuration/age, thresholds of cumulative disturbance, or post-disturbance land-use management. In many other regions undergoing high levels of forest disturbances there are large herbivores that are likely more dependent on mature forests	Thank you for your insightful comment.<br><br>We chose our study species to represent the native assemblage of large herbivores whose potential natural ranges cover large parts of Europe. Our study species represent the four largest native strict herbivore species whose potential natural ranges are not restricted to relatively small parts of the continent (as is the case for herbivore species such as reindeer, ibex, and chamois).<br><br>We agree that digging deeper into more nuanced hypotheses around disturbance characteristics and post-disturbance development would be interesting, and we did

	(e.g. woodland caribou, tapir, etc) and/or subject to very different risks (e.g., Ripple et al. 2015. Collapse of the world's largest herbivores. Science Advances 1:e1400103). Either a more focused framing of the study, or perhaps some broader/nuanced discussion, might help place it more clearly in a global context for international readers.	cover the effects of disturbance patch sizes and cumulative disturbance in our analysis. Yet, since our study is the first to characterize large herbivore responses to forest disturbances based on a representative multi-species dataset at a continental and multi-decadal scale, our primary goal was to summarize the overall response patterns and quantify the impacts of recent disturbance dynamics on habitat conditions for large herbivores in Europe. This should lay the ground for more species-specific, or regionally focused work in future analyses, but we feel this would – if done adequately – expand the current scope and length of the manuscripts very substantially.<br><br>While we agree that considering post-disturbance management effects would be highly interesting, suitable spatially-explicit datasets on post-disturbance management practices are unfortunately not available at the spatial and temporal scales of our analysis.<br><br>We have further revised the framing and discussion to better articulate the relevance of our findings, and to place them more clearly in a broader, global context. We would like to note that while our results conform well with what could be expected based on the general ecology of herbivores and our species more specifically, ours is the first study that shows these relationships at macro-ecological scale, across a wide range of populations, environmental conditions, and human-pressure conditions.<br><br>We have added a sentence to the Discussion section mentioning potential negative impacts on large herbivores that depend more strongly on mature forest stands.<br><br>*Yet, we caution that some large herbivores with a strong dependence on mature forests may respond negatively to forest disturbances (e.g., reindeer Rangifer tarandus, depending on undisturbed forest for lichens as key food items during winter[45])*<br>[L302-305]<br><br>We believe that due to the strong variation in responses across different ecological contexts (also highlighted by our study), the

		transferability of individual local studies could be strongly limited. For example, one of the animals referenced in your comment, tapirs, have also been found to select for early successional forest stands created by disturbance (de Luca & Pardini 2017). Thus, we believe that more broad-scale and multi-species assessments are required for obtaining robust data on how forest disturbances impact large herbivores in other regions, which could serve as direct comparisons with our study. Given the lack of systematic assessments from other regions, we thus added sentences discussing relevant research, but also highlighting this research gap.<br><br>*Local studies from North America found varying responses across species, disturbance types, and seasons*[46–48]*, while forest disturbance impacts on large herbivores in other forested biomes remain understudied*[49,50]*. Thus, more broad-scale and multi-species assessments are needed to better understand how large herbivores respond to forest disturbances in other regions.* [L305-308]<br><br>**<u>References</u>**<br><br>de Luca JR, Pardini R (2017) Use of early and late successional forest patches by the endangered Lowland tapir Tapirus terrestris (Perissodactyla: Tapiridae). Mamm Biol 86:107–114. https://doi.org/10.1016/j.mambio.2017.08.001
**4**	c) The Introduction mentions several variables of interest, including body size, foraging strategy, seasonal variation, disturbance characteristics, environmental gradients, but no hypotheses or predictions are presented to set up tests of these factors or demonstrate how this study builds on previous knowledge for these relatively well-studied species and regions. Rather, the interpretations appear more post-hoc (i.e., weaker inference).	Thank you for this thoughtful comment. We agree that bringing in a priori predictions into the Introduction is useful. We have revised it accordingly, connecting our discussion of underlying mechanisms with (general) predictions about<br><br>*By killing canopy trees, forest disturbances increase light availability in the understory, encouraging the growth of early successional plants, and resulting in higher forage availability for many species*[12,13]*. Due to the seasonal variation in foraging preferences*[14]*, the suitability of disturbed areas for large herbivores may vary considerably throughout*

		the year[15]. Disturbances can intermittently reduce the availability of vegetation cover, and create open habitats in which animals are exposed to predation, human activities, thermal extremes, and snow[16–18]. Yet, depending on disturbance characteristics (e.g., disturbance patch size and remaining deadwood) and vegetation recovery, disturbed areas can provide habitats offering both forage and cover[19]. Thus, large herbivore responses to forest disturbance can be expected to strongly depend on species' feeding ecology and disturbance characteristics, and further show considerable seasonal variation.<br>[L99-108]<br><br>We decided to keep these predictions at a general level to conform to the word limit of the Introduction.
**5**	The authors focus on a behavioural indicator (habitat selection) and make the assumption (mentioned on line 504) that their measure of habitat selection reflects fitness benefits for the focal species. Conclusions around increased carrying capacity (line 78) and "substantial positive and long-lasting effects" (line 243) may require more critical evaluation, since the links between habitat use and fitness or demographic response are not always straightforward (as in concepts of attractive sinks or ecological traps, e.g. Robertson & Hutto RL 2006 Ecology 87:1075–1085). There are many studies emphasizing that the relationship between selection and fitness is complex and may only be proportional under a restrictive set of assumptions (e.g., Matthiopoulos et al., 2015, 2020). We further question whether it is reasonable to compare selection strengths between different years if they represent different decisions made by individuals at different times with different available habitats and (likely) different population densities? Density-dependence in habitat selection will be especially relevant at these large spatiotemporal scales.<br>It seems that large herbivore species such as the 4 examined here are generally well-studied and their population abundances are often monitored for hunting management.	Thank you for raising these important conceptual points. We fully agree that the relationship between habitat selection, fitness, and demographic outcomes is often complex and that habitat selection cannot be interpreted as a direct proxy for fitness. Our intention was not to equate habitat selection with fitness, but rather to use selection strength as an indicator of relative habitat suitability, under the clearly stated assumption that habitat selection reflects informed decisions by animals to improve their fitness (i.e., habitat selection is an indicator of fitness payoffs as expected by the animals, not a direct measure of true fitness outcomes; Northrup et al. 2022).<br><br>To address your points, we have revised the manuscript in several ways.<br><br>First, we added a sentence to the Methods section acknowledging that we could not account for density-dependent processes.<br><br>*We thus follow the common interpretation of model predictions as indicators of relative habitat suitability, but acknowledge that our models do not account for changes in habitat selection due to varying population densities[37,109].*<br>*[L576-578]*<br><br>Second, we have revised statements that could be read as implying a direct demographic or

	Can the authors further support their focus on habitat selection in relation to their conclusions around population-level consequences?<br><br>Matthiopoulos, J., Fieberg, J., & Aarts, G. (2020). Species-Habitat Associations: Spatial data, predictive models, and ecological insights. https://doi.org/10.24926/2020.081320<br>Matthiopoulos, J., Fieberg, J., Aarts, G., Beyer, H. L., Morales, J. M., & Haydon, D. T. (2015). Establishing the link between habitat selection and animal population dynamics. Ecological Monographs, 85(3), 413–436. https://doi.org/10.1890/14-2244.1	fitness response. In particular, we now avoid phrasing that suggests guaranteed increases in carrying capacity and instead frame such outcomes as potential consequences, contingent on other processes (e.g. density dependence, management, mortality risks).<br><br>In the discussion, we now also reference recently published data on population trend estimates derived from hunting bags for large herbivore species in Europe. In this context, we highlight the need for further research that links results from studies such as ours to data on population trends in order to investigate the role of different environmental dynamics.<br><br>*Previous studies have established links between forest disturbance dynamics and large herbivore performance or population trends[44,65]. Habitat suitability improvements caused by forest disturbances across Europe may result in higher potential carrying capacities and even population increases, although demographic responses will further depend on density-dependent processes, management, and mortality risks. Recently published data on country-level trends in hunting bags for European large herbivores (including moose, red deer and roe deer) suggest overall increasing population trends over the last decades, albeit with considerable variation across regions and species[31]. More research integrating information on potential drivers such as climate change, land use dynamics, and forest disturbance dynamics is needed to understand their relative importance for large herbivore population dynamics.*<br>[L370-379]<br><br>Third, we revised the sentences quoted in your comment:<br><br>*Our findings indicate that forest disturbances can have strong attraction effects for large herbivores and that increasing disturbance rates improved habitat suitability for our study species across Europe.*<br>[L78-80]<br><br>*Providing the first broad-scale and long-term assessment of forest disturbance effects on large herbivore habitat selection, we reveal*

		overall positive and long-lasting effects on habitat suitability for the four study species. [L273-275]<br><br>Fourth, regarding comparisons of selection strength across years, we clarify that we do not separately analyze habitat selection for different years, but instead build single models of habitat selection per species and then compare habitat suitability changes predicted by our models. Thus, our comparisons reflect changes in the suitability of forest habitats associated with disturbance dynamics (as mapped by the underlying remote sensing data), rather than temporal changes in habitat selection across time. Nevertheless, as mentioned above, we now explicitly acknowledge that unmodeled density dependence could influence selection patterns.<br><br>Finally, we focused on habitat selection since our primary goal was not to relate forest disturbances to population dynamics, but to leverage our unique continental-scale and multi-species dataset to understand responses in habitat selection and habitat suitability associated with forest disturbance dynamics.<br><br>**References**<br><br>Northrup JM, Vander Wal E, Bonar M, et al (2022) Conceptual and methodological advances in habitat-selection modeling: guidelines for ecology and evolution. Ecol Appl 32:e02470. https://doi.org/10.1002/eap.2470
**6**	We are confused around the treatment of individual heterogeneity in habitat selection, which can be an important source of variation. It seems that individual-level information (except for sex) only enters the random forest models in the generation of background 0's within home ranges. The subsampling procedure (lines 428-430) would also lead to a data imbalance at the individual level which may be problematic if there is unmodeled individual heterogeneity.	We agree that individual heterogeneity can be an important source of variation in habitat selection and appreciate the opportunity to clarify our approach and rationale for how we deal with this issue in our study.<br><br>Our primary objective was to leverage the massive tracking datasets containing large numbers of individual animals to infer species-level habitat selection patterns across broad spatial and environmental gradients. We chose a machine learning framework instead of a more "traditional" approach based on resource selection functions built with, e.g., mixed effect models designed to control for

		individual-level variation, for the following reasons:<br><br>Given the large datasets covering wide environmental gradients we had available, our main modeling challenge was to capture complex non-linear responses to forest disturbance and their variation with several environmental factors. At the same time, the large number of individuals in our datasets strongly reduces the impact of individual-level variation on habitat selection models, since any given individual contributes only small amounts of data to our models.<br><br>While it is theoretically possible to account for individual-level variation through random effects in approaches such as generalized additive mixed effects models (GAMMs), we would like to point out that this would require fitting random slope effects for each individual (since random intercepts alone do not sufficiently account for varying selection across individuals; Muff et al. 2020). Fitting GAMMs with ca. 230-1100 individuals, a few million training points (tracking locations + background points), and ca. 20 predictors, including random slopes would imply enormous computational costs and would likely result in model convergence issues. Machine learning approaches like random forests, on the other hand, provide an effective method for such problems due to their power to uncover complex relationships and interactions in very large datasets.<br><br>Furthermore, we carefully considered potential sources of bias, including individual-level variation, during the selection of training data for model fitting:<br><br>First, to account for individual heterogeneity in habitat availability, we generated background (available) locations within individual- and track-specific home ranges, thereby conditioning habitat selection on the environments each individual experienced. This approach implicitly accounts for individual differences in space use, movement constraints, and landscape context.<br><br>Second, we down-sampled tracking locations at the animal level within environmental

		clusters to balance representation across broad environmental contexts and avoid dominance of regions or individuals with exceptionally dense data. This procedure equalizes the contribution of environmental clusters while at the same time balancing across individuals within environmental clusters. We prioritized balancing across environmental clusters since we considered this to be the most important source of bias in our dataset (given the strong sampling bias across environmental gradients on the one hand, and the large number of individuals on the other).<br><br>We now clarify this design choice and its implications in the Methods section.<br><br>*We balanced the presence-background datasets across environmental clusters to avoid biasing models towards environmental conditions with more available data. Since our dataset comprised large numbers of individuals per species, but the number of individuals and tracking locations varied strongly across environmental clusters, we considered this environmental bias the primary source of bias for our models. Therefore, we down-sampled data at the animal level so that the number of locations was the same across clusters. This allowed us to keep data from as many individuals as possible, thus enhancing species-level representativeness[82], while balancing the contribution of different environmental clusters and individual animals within them [83,84].*<br>[L483-490]<br><br>**<u>References</u>**<br><br>*Muff S, Signer J, Fieberg J (2020) Accounting for individual-specific variation in habitat-selection studies: Efficient estimation of mixed-effects models using Bayesian or frequentist computation. J Anim Ecol 89:80–92.* [*https://doi.org/10.1111/1365-2656.13087*](https://doi.org/10.1111/1365-2656.13087)
**7**	We have some confusion/concern around the model predictions, specifically:<br><br>a) No assessment of model adequacy is presented for the final models (AUC is only presented in the supplement S2 in the context	Thank you for your helpful comment, we also think it is a good idea to report validation scores for our final models.<br><br>Since AUC values are not informative of model performance in absolute terms in the

	of tuning random forests). And comparing figures 4 and 5 suggests some significant extrapolation of predictions beyond the ranges of animal tracking for most species (and perhaps some uneven sampling across forest cover and disturbance, although hard to discern). This should be more transparently acknowledged/discussed.	context of presence-only habitat selection models (due to their dependence on prevalence and background sampling extent defined by the analyst) and should only be used for relative comparisons such as during model tuning, we chose to validate our models based on the Continuous Boyce Index (CBI), designed for validating presence-only models. We cross-validated models based on the environmental clusters of individuals we derived, thus explicitly testing the transferability of models.<br><br>We added a sentence to our Methods section in which we report CBI scores obtained from these cross-validations of our final models. These scores indicated excellent predictive performance and transferability of our models.<br><br>*After hyperparameter tuning, we assessed the transferability of our models based on a cross-validation with environmental clusters as folds[106], which indicated excellent predictive performance and transferability of our models (mean Continuous Boyce Index 0.99, 0.98, 0.91, and 0.89 for European bison, moose, red deer, and roe deer, respectively).* [L566-570]<br><br>For our analysis of habitat suitability trends across the species ranges, we re-ran models built without the "years since disturbance" variable in order to avoid a truncation bias due to a lack of information on disturbances happening prior to 1986 (see our response to comment #14 by reviewer #1). Hence, we performed separate model tunings and cross-validations for these models, which we also report in the Methods section.<br><br>*We applied the same tuning and cross-validation approach described above, which again indicated excellent model transferability across environmental clusters (mean Continuous Boyce Index from cross-validation: 0.98, 0.95, 0.94, and 0.88, for European bison, moose, red deer, and roe deer, respectively).*<br>[L648-651]<br><br>Regarding potential issues associated with model projections across the species' potential range extents, we agree that this limitation

		should be more clearly acknowledged. We added a sentence to the Discussion highlighting the issue of model extrapolation:<br><br>*Finally, projecting our models across the potential range extents of species required considerable extrapolation in some cases, particularly for European bison. Although we found highly similar trends in habitat suitability between current and potential range extents and cross-validations suggested very good transferability of our models, we caution that such model extrapolations are associated with higher levels of uncertainty*[69]*.* [L415-419]
**7**	Can the authors further justify the rationale/validity of holding all environmental variables at 2023 levels and predicting suitability based only on forest disturbance for 2000-2023?	The goal of this counterfactual prediction was to isolate the effects of forest disturbances on habitat suitability dynamics. If other predictors (i.e., human pressure variables, land cover, and carnivore distributions), necessary as control variables when training our models, would not be held constant, the contribution of forest disturbance impacts on habitat suitability trends could not be easily quantified. We hope this clarifies this methodological choice.
**8**	The habitat selection models were built at the 3rd order of habitat selection (i.e., within home range) but the predictions seem to more at the 2nd-order of selection (i.e., home range location, 100 km-wide hexagons), although this is not clearly stated. Can the authors clarify/justify this apparent discrepancy in scale?<br>Furthermore, we wonder about the use of predictors at the home range scale (Extended Table 1), which seems at odds with subsequent interpretation of results based on foraging decisions within home ranges. If the buffer radius was almost as large as the home range, the sampled points will have (nearly) identical values and thus little variation for assessing within-home-range foraging behaviour (not to mention the high spatial autocorrelation among points within home ranges). In general, we felt the moving window averaging method needed to be more clearly explained.	We agree that these scale-related issues required clearer explanation. We have revised the Methods section and figure captions to explicitly clarify both points.<br><br>Our habitat-selection models were indeed fitted at the third order of selection, comparing used GPS locations to available locations sampled within individual-specific home ranges. We then spatially aggregate model predictions to summarize broad-scale changes in habitat suitability across Europe. Our maps in Figure 4, however, do not represent modeled home-range placement decisions (i.e., second order selection), but rather the spatial distribution of predicted within-home-range suitability under different forest conditions. For aggregating trends at the scale of species current and potential range extents per year (Figure 3), we weighted the contribution of grid cells by the frequency of disturbed/undisturbed forest areas, thus taking into account varying frequency of forest cover and disturbance.

		We added sentences to the captions of Figures 3 and 4 to clarify this.<br><br>Figure 3:<br>*Predictions of fine-scale habitat suitability were derived for a stratified sample of disturbed and undisturbed forest pixels covering the current and potential range extents of species.*<br>[L245-246]<br><br>Figure 4:<br>*Predictions for fine-scale habitat suitability changes are summarized at the scale of 8,860km² hexagons to visualize regional variation.*<br>[L264-265]<br><br>Regarding our use of multi-scale predictors, we agree that our original description did not sufficiently explain the rationale for including predictors at different spatial scales. Our intent was to capture both fine-scale foraging conditions and broader contextual effects (e.g. overall disturbance availability, landscape composition, human pressure) that influence within-home-range habitat use. Adding variables which summarize habitat availability at broader spatial scales (e.g., the scale of animal home ranges) is a well-established approach to enable models to learn how habitat selection varies with environmental gradients represented by different individuals and regions in broad-scale tracking datasets (so-called functional responses in habitat selection; Matthiopoulos et al. 2011; Paton and Matthiopoulos 2016).<br><br>Values of these variables will vary within individuals since they are derived based on spatial moving windows (e.g., forest / cropland cover will be different at the edges vs. the center of an individual's home range). In addition, they are primarily included to introduce information characterizing gradients *across* individuals (representing different landscape contexts in our dataset). We chose

		species' home range sizes to define window extents, since this provides an ecologically meaningful summary of habitat availability which introduces information on a broader-scale selection process (i.e., home range placement) into our fine-scale habitat selection models.<br><br>We have revised our Methods section to clarify our rationale.<br><br>*Adding home-range scale predictor variables allowed our models to learn how habitat selection varied with habitat availability reflecting broader-scale selection processes (i.e., home-range placement). Considering variation of habitat selection due to varying resource availability across different landscape contexts (so-called functional responses in habitat selection[85]) are particularly important to consider in analyses covering large environmental gradients[84,86].*<br>[L500-505]<br><br>**<u>References</u>**<br><br>Matthiopoulos J, Hebblewhite M, Aarts G, Fieberg J (2011) Generalized functional responses for species distributions. Ecology 92:583–589. https://doi.org/10.1890/10-0751.1<br><br>Paton RS, Matthiopoulos J (2016) Defining the scale of habitat availability for models of habitat selection. Ecology 97:1113–1122. https://doi.org/10.1890/14-2241.1
**9**	line 76 "Across Europe ..." sentence seems out of place, not well connected to flow of abstract	We have revised our Abstract, including the sentence referenced in your comment. Now, the sentence reads:<br><br>*Based on model projections across the species' range extents, we found positive but regionally varying effects of forest disturbances on habitat suitability between 2000-2023.*<br>[L77-78]
**10**	the unique spatial and temporal scale of the data could be more strongly emphasized in the abstract, as on line 110 (in place of generic statements such as navigating trade-offs between resources and risk)	We have revised our Abstract. In the second sentence, we now highlight the unique scale of our analysis.<br><br>*We combined animal tracking data comprising 3,069 individuals of four large herbivores in Europe (European bison,*

		moose, red deer, roe deer) with satellite-based forest disturbance maps to perform the first continental, multi-decadal assessment of large herbivore responses to forest disturbance. [L70-73]<br><br>In addition, we have removed the statement regarding resource-risk tradeoffs to focus on reporting the most interesting and relevant results originating from our analysis.<br><br>*Despite strong intraspecific variation, all species showed an increased selection of disturbed areas for 35+ years post-disturbance. Selection patterns closely reflected species-specific foraging strategies. All species selected more strongly for smaller disturbance patches and selection was influenced by the availability of alternative foraging habitats (i.e., grasslands and croplands).* [L73-77]
**11**	acknowledging the "Methods last" format of the journal, it would still be helpful to make brief mention of the general approach to modelling earlier in the paper, as many statements are hard to interpret without some methodological context	We agree that providing more information at the beginning of the Results section is helpful. Thus, we have extended our summary of methods and underlying datasets in this section.<br><br>*We used the largest animal tracking dataset ever compiled for large herbivores (3,069 tracked animals, 19.3 million locations) to build habitat selection models for European bison, moose, red deer, and roe deer based on the random forest algorithm. We characterized habitat dynamics based on datasets derived from satellite image time series, including annual, high-resolution maps of forest disturbances[28] and spectral vegetation indices[15]. These forest disturbance maps capture stand-replacing forest disturbance events at 30m spatial resolution annually between 1986-2023. In the context of our analysis, we considered all areas with a detected disturbance event as disturbed forest, while all other areas were considered undisturbed forest. Using selection strength as an indicator of relative habitat suitability[37], we created monthly predictions of habitat suitability between 2000 and 2023 across representative samples of disturbed and*

		undisturbed forest areas within the species' current and potential range extents in Europe. To assess the species-specific responses to forest disturbances over time, we summarized habitat suitability dynamics after disturbances, focusing on the current range extents of species. To aid comparability across species, we quantified suitability changes as relative changes after disturbance and expressed effects in standard deviations of forest habitat suitability.<br><br>[L145-158]
**12**	Fig 1: please explain/justify why 40th to 60th percentile range used to show responses	Thank you for raising this issue. Your comment made us reconsider our approach to visualizing and communicating variation in disturbance responses in our dataset.<br><br>While our previous approach highlighted the central tendency of responses, we agree that providing a complete picture reflecting the full distributions of responses is informative. Thus, we have made adjustments to Figure 1, now showing three percentile intervals (40-60th, 25-75th, and 10-90th percentile). We have changed the Figure caption accordingly.<br><br>*A: Long-term disturbance responses of habitat suitability, with the black line indicating median suitability and the grey ribbons the 10-90th, 25-75th, and 40-60th percentile ranges, respectively.*<br>[L163-165]<br><br>In addition, we added an Extended data Figure (3), which summarizes how often disturbed areas were predicted to feature positive/negative suitability values in comparison to pre-disturbance conditions.<br><br>To better represent the considerable variation in disturbance responses characterized by our models, we have added statements summarizing these findings to the Results and Discussion sections.<br><br>*Despite overall positive responses, our models predicted decreased habitat suitability compared to pre-disturbance conditions in 36%, 29%, 17%, and 13% of cases for roe deer, European bison, red deer, and moose, respectively (Extended data Fig. 3).*<br>[L176-179]

		Despite considerable intraspecific variation and declines in habitat suitability after disturbance in some cases, we found overall positive responses by all species, in line with previous local assessments [12,44].<br>[L300-302]
**13**	Fig 2: please explain the bootstrapping details for obtaining 95% CI	Please note that we revised our approach for evaluating partial dependence plots (PDPs). We changed one-dimensional PDPs per variable to two-dimensional PDPs across bins of the year since disturbance variable and all other predictors.<br><br>This allows us to provide a more complete picture of the relative influence of the environmental variables at different stages of forest recovery after disturbance (see also our response to comment #18 by reviewer #1).
**14**	paragraph starting line 191 - why are there no references to figures/tables to support these results?	We have revised our Results section and have moved the summary and interpretation of environmental variable effects (i.e., variables relating to climate, human pressure, topography, and land cover) to a newly created Supplementary Note (S2), since a detailed discussion of all environmental variable effects would have gone beyond the scope of the main text. In this Supplementary Note, we provide a detailed discussion of environmental variables and their effects, including references to the associated Extended data Figures (5-8), as well as to relevant literature supporting our interpretations.
**15**	Fig 3: why is current range not shown for roe deer?	Thank you for pointing this out. We added a sentence to the Figure caption explaining why only one trend line is visible for roe deer.<br><br>*For roe deer, lines and points are strongly overlapping due to the current and potential range extents being almost identical.*<br>[L251-252]
**16**	Fig 4: should the caption say 2000-2023?	Yes, indeed. Thank you for spotting the typo.
**17**	lines 263-265: This could explain why the peaks of bison and moose selection differ, but then why is bison selection significantly elevated even after 37 years, but moose selection is in decline? Further, woody species (like Rubus) are not typically found one year post disturbance; herbaceous species	Thank you for pointing out this imprecision in the discussion of our results. You are completely right that the establishment of Rubus in disturbed areas may take a few years. We changed the sentence to reflect this, interpreting the increased selection for around five years after the disturbance as a signal of a

	(graminoids and forbs) usually establish first. We are concerned that inferences are over-stepping the strength of evidence.	preference for grazing on graminoids and forbs and browsing on pioneer woody species, as documented by diet analyses of European bison. In addition, we provide a potential explanation for the extended selection of disturbed areas by the species for more than 35 years, which may reflect a preference for open and lower forest stands as highlighted by previous studies.<br><br>*The response of European bison, with a clear peak in habitat suitability in the first few years after a disturbance event, match well with its preference for grazing and browsing on pioneer woody species such as Rubus ssp.*[39]*, while the continued selection beyond this initial peak may reflect a preference for relatively low and open forest stands*[42,43]*.*<br>[L293-296]
**18**	line 335 - missing period between sentences	Thank you, we fixed the typo.
**19**	line 347 - delete "be"	We fixed the typo. Thank you for spotting it.
**20**	the Methods imply both 5 available points to 1 used, and balance between presence/background, please clarify	Thank you for the helpful comment. Your comment pointed out a potential source of misunderstanding in our Methods description.<br><br>We used down-sampled random forests (Valavi et al., 2022), which are designed to alleviate potential issues associated with class imbalance in random forests built with presence-background datasets. In this approach, a random subsample of background points is used within each tree of the model (in our case, 300 trees were fitted). Thereby, the number of presence and background points are balanced at the level of each tree, while the complete set of background samples is still used during model training.<br><br>We have revised our description of down-sampled random forests to make this distinction clearer.<br><br>*Since class imbalances can negatively affect random forest model performance, we used down-sampled random forests*[105]*. In this approach, a subsample of background locations is used to balance both classes (presence/background) within each tree of the model, while the full set of background points is used during model training.*<br>[L561-564]

**21**	line 402: justification for buffer size? is 7 hours long enough to mitigate autocorrelation?	We based our selection of minimum sampling intervals based on the following factors. First, we tested the autocorrelation of step lengths in our dataset as an indicator for spatiotemporal autocorrelation. Based on this analysis, we observed a strong decline in autocorrelation beyond time lags of 2-4 hours. Then, we chose seven hours as a conservative threshold to avoid biases associated with intervals aligning with 24-hour circadian behavioral rhythms.<br><br>We have revised our Methods section to provide a clearer justification of our approach.<br><br>*Second, we sub-sampled the tracking data to reduce autocorrelation between locations[75]. We tested the correlation of step lengths for different time lags based on animals with high sampling rates (i.e., <2 hours). To ensure independence of observations and avoid biases associated with 24-hour behavioral rhythms[76], we chose a conservative 7-hour minimum sampling interval, exceeding the ca. 2-hour decay in autocorrelation observed in our data (see Supplementary Note S4 for more details).*<br>[L454-459]<br><br>In addition, we added Supplementary Note S2 in which we summarize results of our test regarding step length autocorrelations for all species.
**22**	line 426: why were background points restricted to forest areas?	Thank you for pointing this inconsistency out. This passage stemmed from earlier versions of the manuscript. In our current analysis, we did not restrict background sampling to forest areas but sampled them across the full track-wise home ranges. We revised the section accordingly, removing this statement.<br><br>*Within these buffered home ranges, we sampled five random locations per tracking location as background points, assigning the recording time of tracking locations to the background points[15].*<br>[L479-481]
**23**	lines 449-450: mismatch in temporal scale between annual disturbance data and monthly resolution of analysis. Could some locations in undisturbed forests be classified as recent	Thank you for highlighting this issue. You are correct that there is indeed a temporal mismatch between the annual resolution of our disturbance maps and the monthly

	disturbance if the disturbance occurred at a later month? Some months misclassified?	resolution at which we analyze habitat selection.<br><br>We added a sentence to our Discussion to highlight this as a limitation.<br><br>*Since forest disturbance maps were only available at an annual temporal resolution, we could not eliminate uncertainty from cases in which disturbances happened in the months after a tracking location was recorded.* [L409-411]
**24**	lines 456-459: justification for choices of -100 and 100? Results could be sensitive to this especially since a lot of "undisturbed" forest is probably still disturbed from the perspective of focal species.	Thank you for raising this issue. We fully understand your concern regarding the sensitivity of our results to this choice.<br><br>Since we shared your concern, we re-ran our analysis to investigate how changes to these values would affect our results. Specifically, we were concerned that high values for the “years since disturbance” variable in undisturbed areas (i.e., not experiencing disturbances between 1986-2023) could bias suitability estimates in these areas. Thus, we chose to keep the maximum years since disturbance at its original value (37 years). In addition, we replaced the binary forest/non-forest variable with a variable containing three categories: non-forest, undisturbed forest (not experiencing a disturbance between 1986-2023), and disturbed forest (experiencing a disturbance between 1986-2023). Including this variable gives the random forest model more flexibility, enabling it to fit interactions between the categories and other predictors for characterizing varying habitat selection in disturbed vs. undisturbed forests. Importantly, the results obtained from these new runs of models were almost identical to the results from our earlier version of the manuscript.<br><br>The robustness of our results to choices around the “years since disturbance” variable is further testified by the additional analysis we performed in the context of projecting habitat suitability changes across the species’ range extents. Specifically, we re-ran models built without the “years since disturbance” variable in order to avoid a truncation bias due to a lack of information on disturbances happening prior to 1986 (see our response to comment #14 by reviewer #1 for more

		details). Disturbance responses obtained from these models were highly similar to those predicted by models including the "year since disturbance" predictor.<br><br>We explain the lack of sensitivity of our models to these changes with the large amounts of data we had available in combination with the high flexibility of random forests. We revised our Methods and Results sections to reflect all changes associated with our adjustments made to the habitat selection models.<br><br>Finally, regarding the characterization of differences in "undisturbed forest areas" / mature forest stands: we included the spectral-temporal metrics derived from Landsat satellite images partly for this purpose, since they also allow characterizing variation of forest structure in mature stands (Oeser et al. 2020). We added a sentence to our Methods section to explain this.<br><br>*Landsat-based spectral-temporal metrics are further useful to capture variation in forest structure in mature forest stands[92].*<br>[L528-529]<br><br>**References**<br><br>Oeser J, Heurich M, Senf C, et al (2020) Habitat metrics based on multi-temporal Landsat imagery for mapping large mammal habitat. Remote Sens Ecol Conserv 6:52–69. https://doi.org/10.1002/rse2.122
**25**	lines 476-478: scale of animal home range? The method is not clear. Did the authors create a buffer around used and available points with a radius equal to home range?	Thank you for pointing out the ambiguity. We explain the approach in the first paragraph of this section.<br><br>*We derived home-range scale predictors by applying moving window averaging to the raster datasets[84], using window sizes reflecting our median home range sizes per species.*<br>[L498-500]<br><br>To better clarify the meaning of moving window averaging in the context of land cover variables, we rephrased the sentence.<br><br>*We calculated the availability of land-cover types at the scale of animal home ranges to characterize landscape composition (i.e., the*

		relative proportion of land cover types in neighborhoods corresponding to the species' home range sizes), including the availability of alternative foraging habitats outside forests (i.e., croplands and grasslands).<br>[L540-544]
**26**	line 480: typo - "large habitat" (missing "ungulate")	Thank you for spotting the typo.<br><br>*We included distribution maps indicating the (permanent) presence of grey wolf, Eurasian lynx, and brown bear to capture potential effects of predator presence on habitat selection.*<br>[L545-546]
**27**	line 513: would be helpful to briefly explain PDPs	We agree and added a sentence explaining PDPs.<br><br>*PDPs visualize the marginal effect of a predictor variable by showing how model predictions change as one or more variables vary while averaging out other predictors*[110]*.*<br>[L588-589]
**28**	lines 548-549: Predictions were made for both sexes, but which results are shown in e.g. Figure 4?	To infer species-level responses, we averaged the two predictions made separately for both sexes. We added a sentence to clarify this.<br><br>*We made predictions for all months between 2000 and 2023, including predictions for both sexes, which we averaged to infer species-level responses.*<br>[L630-631]
**29**	line 565: this sentence is confusing as written: "in undisturbed forest areas, the only variable that was allowed to vary across years was the area of recently disturbed forest."	We agree that the wording here was potentially confusing. We have edited the sentence to make it clearer why the area of recently disturbed forest was the only variable inside undisturbed areas affected by disturbance dynamics, thus being allowed to vary across time in our predictions.<br><br>*To isolate the effect of forest disturbances on habitat suitability, we used a counterfactual prediction approach. Specifically, we kept all predictor variables unrelated to forest disturbances static (with a target year of 2023) and predicted suitability trends across all sample pixels between 2000 and 2023. This meant that in undisturbed areas (i.e., with no detected disturbance between 1986-2023), the only variable that varied across time was the area of recently disturbed forest,*

		describing the number of pixels which were disturbed ≤10 years ago within neighborhoods reflecting the species' home range sizes. In areas with detected disturbances, all variables describing forest disturbance dynamics varied annually across the prediction time frame (patch size, disturbance agent, disturbance severity, and the Landsat-based spectral-temporal metrics), while all others were kept static.<br>[L654-662]
**30**	lines 566-569: undisturbed as of 2023 or undisturbed throughout the entire period of disturbance data? If the former, why weren't other disturbance metrics varied? If the latter, wouldn't the area of recently disturbed forest always be zero? Please clarify the specific meaning of terms like "forested" or "undisturbed" (time period, spatial scale, etc.).	Thank you for your helpful comment. Please see our answer to the comment above for the changes made to this section. We rephrased both sentences to make it clear in which areas the different variables varied in our predictions and how we define undisturbed areas in this context.
**31**	Supplement S1: clustering method not clear, including use of PCA vs. individual environmental variables. Please explain "97% of the observed variance" in what? the environmental attributes of the tracking locations? What is the overall purpose of clustering?	We agree that the description of the clustering procedure was not sufficiently clear.<br><br>Regarding the overall purpose of clustering: clustering was used to account for the strong environmental sampling bias present in our dataset during model training and validation. Specifically, we used clustering to group and subsample tracking locations from environmentally similar strata, thus ensuring that habitat-selection models were not disproportionately influenced by overrepresented environmental conditions. In addition, it allowed for testing the transferability of models across environmental clusters during model tuning and validation based on cross-validation.<br><br>*In a first processing step, we grouped animals into species-wise environmental clusters. This was done to allow balancing datasets during model training and validation and thereby account for environmental sampling bias present in our dataset.*<br>[L442-444]<br><br>Regarding the meaning of variance explained in this context: A principal component analysis (PCA) was applied to the full set of clustering variables (i.e., forest cover, terrain ruggedness, winter temperature, and human population density) extracted at GPS

		locations. The first two principal components, explaining 97-99% of the variance in these environmental attributes, were retained for clustering. This variance refers to the multivariate environmental space occupied by the tracking locations, not to animal movement or habitat selection. We revised the respective sentence in Supplement S1 to make this clearer.<br><br>*These PC components explained 97% of the observed variance in clustering variables for European bison, 99% for moose, 99% for red deer, and 98% for roe deer*<br>[Supplementary Note S3]
**32**	Why are the maps of predators not shown anywhere? We are concerned that these may overestimate predation risk (given low abundance / uncertain occupancy?). The emphasis on the "weak negative effect ... in some cases" (line 184) may be stretching the strength of evidence presented?	Thank you for raising this point.<br><br>To be more transparent about the distribution maps used for our analysis, we added Supplementary Figure 5, which shows the distribution maps used as predictors of carnivore presence. We only included areas classified as “permanent presence” in the surveys to remove areas with uncertain or sporadic occupancy. To also show the expert range maps and potential distribution maps used to identify range extents for the four large herbivore species targeted in our analysis, we added Supplementary Figure S7.<br><br>We further agree that the interpretation of carnivore effects based on our models requires a more cautious framing, given the broad-scale nature of the predictors and their overall weak effects in our models. Thus, we revised our manuscript, including the Discussion section, to provide a more cautious interpretation, better reference our findings to relevant literature, and better highlight the need for further research to obtain more robust insights into carnivore effects.<br><br>*Due to the continental and multi-decadal extent of our analysis, we could not assess fine-scale effects of some drivers which are likely important in mediating the suitability of disturbed areas for large herbivores. We found only weak associations between broad-scale distribution maps for large carnivores and habitat selection by our study species (i.e., relative effects on habitat suitability of up to 0.3 vs. up to 2.0 standard deviations for*

		other variables). Our models indicated negative effects of carnivore presence on large herbivore habitat suitability, with the strongest relative effects of bear presence on moose, wolf presence on red deer, and lynx presence on roe deer. These are plausible patterns given the expected dominant predator-prey relationships between these pairs of species[55–57]. According to our models, carnivore effects were weaker in recently disturbed areas and strongest ca.5-25 years after disturbance, which may point towards increased predation risk and decreased selection associated with densely-vegetated re-growing stands[58]. Available studies suggest variable impacts of carnivore presence on large herbivore use of disturbed forest areas[58–60], which may further interact with effects of hunting[61]. Thus, studies integrating fine-scale datasets from carnivores and herbivores across different ecological contexts are needed to gain more robust insights into the effects of predation risk. [L342-354]
